# Tetrapodal Anion Transporters †

**DOI:** 10.3390/molecules25215179

**Published:** 2020-11-06

**Authors:** Alexander M. Gilchrist, Lijun Chen, Xin Wu, William Lewis, Ethan N.W. Howe, Lauren K. Macreadie, Philip A. Gale

**Affiliations:** 1 School of Chemistry (F11), The University of Sydney, Sydney 2006, Australia; alex.gilchrist@sydney.edu.au (A.M.G.); lijun.chen@sydney.edu.au (L.C.); xin.wu@sydney.edu.au (X.W.); w.lewis@sydney.edu.au (W.L.); ethan.n.howe@gsk.com (E.N.W.H.); lauren.macreadie@sydney.edu.au (L.K.M.); 2 Sydney Analytical, The University of Sydney, Sydney 2006, Australia; 3 The University of Sydney Nano Institute (Sydney Nano), The University of Sydney, Sydney 2006, Australia

**Keywords:** supramolecular chemistry, lipid bilayer, anion transport, anion-selective transport

## Abstract

Synthetic anion transporters that facilitate chloride transport are promising candidates for channelopathy treatments. However, most anion transporters exhibit an undesired side effect of facilitating proton transport via interacting with fatty acids present in the membrane. To address the limitation, we here report the use of a new tetrapodal scaffold to maximize the selective interaction with spherical chloride over binding the carboxylate headgroup of fatty acids. One of the new transporters demonstrated a high selectivity for chloride uniport over fatty acid-induced proton transport while being >10 times more active in chloride uniport than strapped calixpyrroles that were previously the only class of compounds known to possess similar selectivity properties.

## 1. Introduction

The development of selective anion transporters with potential application in the treatment of diseases, such as cystic fibrosis and cancer, continues to attract significant interest and research efforts [1,2,3,4]. In cystic fibrosis, synthetic transporters have the potential to act as “channel replacement therapies” by replacing the function of faulty cystic fibrosis transmembrane conductance regulator channels and ameliorating the disease symptoms [5]. Synthetic transporters have been shown to facilitate the transport of chloride across epithelial cell membranes in cystic fibrosis cells with additivity to channel-targeting drugs [6].

In other cell lines, chloride transport has been shown to trigger apoptosis [7,8,9]. Compounds capable of transporting both protons and chloride have been shown to interfere with autophagy by changing lysosomal pH, resulting in impairment of lysosomal activity [10,11,12]. 

**Figure 1:**
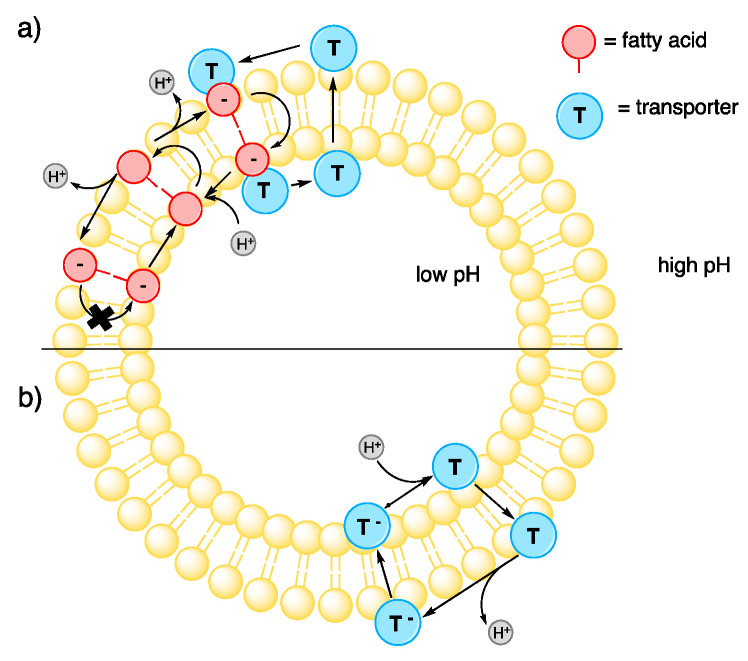
(**a**) Transporter-facilitated fatty acid flip-flop pathway and (**b**) the receptor acting as a weak-acid protonophore, which can both result in the dissipation of a pH gradient across a lipid bilayer.

We have been interested in developing selective transporters that are capable of transporting chloride but not perturbing pH gradients within cells. These compounds may find application as therapeutics or as tools to study membrane transport processes [13]. We found that in addition to anionophores functioning as weak-acid protonophores (Figure 1b), the transport of fatty acid carboxylates across lipid bilayers, and their subsequent protonation and diffusion back across the membrane, is an important pathway contributing to pH gradient dissipation by anion transporters (Figure 1a) [14]. However, we found that receptors with a higher degree of encapsulation around their anion-binding site tend to show higher selectivity for chloride vs. proton transport [13]. This can result in moderately selective chloride transport, even in the presence of fatty acids [13,15]. Tren-based tris-urea and -thiourea tripodal anion transporters that can bind chloride ions by six hydrogen bonds have been shown to be an effective and selective family of anion transporters [16,17,18,19]. Inspired by previous results, we here report the design and synthesis of a class of (thio)urea anion receptors/transporters based on a new tetrapodal scaffold, which allows chloride to be bound by four (thio)urea motifs stabilized by eight hydrogen bonds. Gratifyingly, despite the weaker anion binding affinity of the tetrapodal receptors compared to analogous tripodal compounds, a significantly improved selectivity for chloride uniport over fatty acid-induced proton transport has been found for tetrapodal thiourea with bulky *tert*-butyl substituents. This compound also exhibits a potent chloride uniport activity being >10 times more efficient than previously reported strapped calixpyrroles, which were the only class of compounds known to exhibit similar chloride uniport selectivity properties [15].

## 2. Results and Discussion

### 2.1. Synthesis

**Figure 2:**
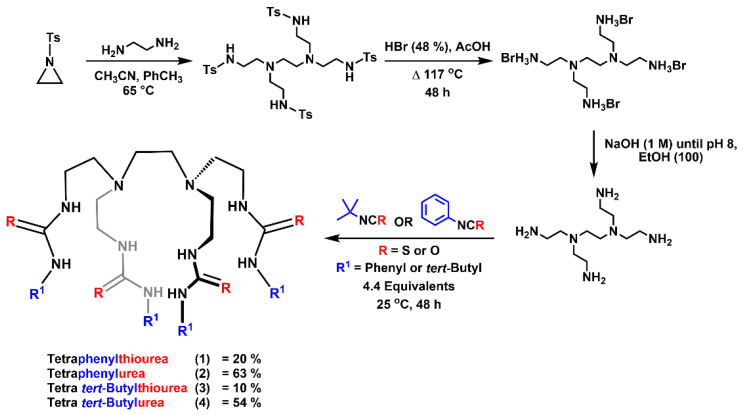
The synthetic scheme of receptors **1**–**4**, where (R) denotes either a urea group (O) or a thiourea group (S) and ′R^1′^ represents the pendant phenyl or *tert*-Butyl functional group.

Receptors **1**–**4** (Figure 2) were synthesized following a four-step synthetic pathway. First, following previously reported methods, ethylenediamine was reacted with 5.0 equivalents of *N*-tosylaziridine to form tetrakistosyl sulfonamide [20], which was subsequently deprotected to form the bromine salt [21]. The bromine salt was neutralized to liberate the free amine and reacted with 4.4 equivalents of the desired iso(thio)cyanate, in either dry dichloromethane or acetonitrile, to produce the tetrapodal transporters. The final product was purified through a series preparative thin layer chromatography separations or washings, see the electronic supplementary information (ESI) for full synthetic details.

### 2.2. Crystallography

**Figure 3:**
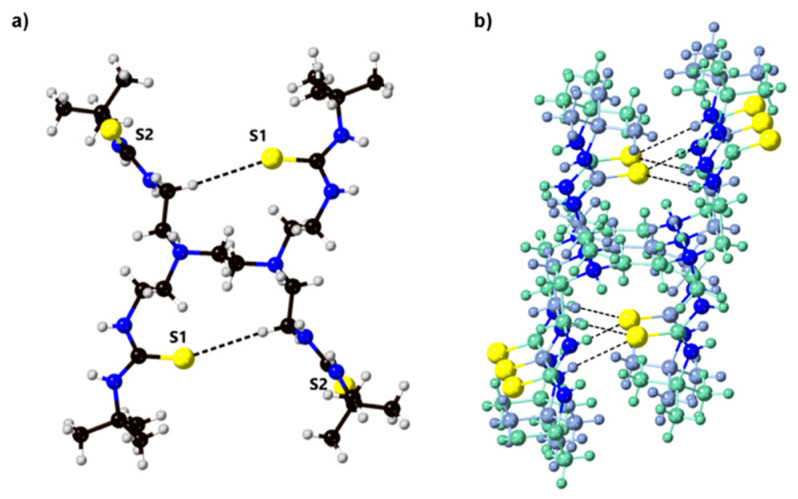
(**a**) X-ray crystal structure of **3** (black: carbon, grey: hydrogen, blue: nitrogen, yellow: sulfur) and (**b**) intermolecular hydrogen bonding between **3** forming columns of the receptors in the solid-state (each receptor is outlined in either teal or blue-grey, yellow: sulfur).

Single crystals of **3** and **4** were crystallized by slow evaporation of DMSO solutions of the receptors. Compound **4** crystallizes in the triclinic space group *P*-1 with one formula unit contained in the asymmetric unit. Close N-H⋯O hydrogen bonds (D⋯A 2.827(4)–3.075(3) Å) exist between adjacent units, forming $R\frac{1}{2}\left(6\right)$ motifs and propagate along the *b* axis, creating columns of transporters. These motifs also exist between close S-H⋯O hydrogen bonds ((D⋯A 3.370(11)–3.552(12) Å) within **3**, propagating along the a axis (Figure 3). The next closest interactions stabilizing the structure exist between the sulfur atoms and the alkyl hydrogen atoms. The C-H⋯S distance is 3.13 Å and exhibits a D-H⋯A angle of 171.1°, lying within the range expected for C-H⋯S hydrogen bonds [22].

### 2.3. Anion-Binding Studies

Proton NMR titrations were used to assess the ability of the receptors (**1**–**4**) to bind Cl^−^, HCO_3_^−^, SO_4_^2−^, or H_2_PO_4_^−^ and HP_2_O_7_^3−^ anions. The studies consisted of titrating the anion as the tetrabutylammonium salt (or in the case of bicarbonate, the tetraethylammonium salt) into the host solution consisting of the receptor with a constant concentration (5 mM) in a DMSO-d_6_/0.5% water solution. Binding constants (*K*_a_) of the receptor:anion complexes that formed during the studies were determined using the online web-applet BindFit [23]. 

Across the series, no binding was observed for nitrate. Binding constants could not be determined for dihydrogen phosphate due to complex equilibria in solution that could not be fitted adequately to a binding model. Similarly, previously reported tripodal systems and receptors with multiple hydrogen bonding sites displayed similar behavior, where proton transfer between the bound dihydrogen phosphate anion and the free dihydrogen phosphate in solution generates the monohydrogen phosphate complex [17,24]. Complex binding equilibria were observed for the less acidic urea receptors **2** and **4** in the presence of sulfate. In contrast, the more acidic thiourea receptors **1** (*K*_11_ 1000 M^−1^, *K*_21_ 56 M^−1^) and **3** (*K*_a_ > 10^4^ M^−1^) exhibited strong binding (Table 1).

The initial sigmoidal curve was seen in the sulfate binding isotherm of receptor **1** and, coupled with saturation at approximately one equivalence of the titrant, is indicative of 2:1 (two receptor **1** molecules:one sulfate anion) complexation at the initial stage followed by the formation of the 1:1 complex (one receptor **1**:one sulfate anion) (ESI Figure S53). The interactions of the receptors with pyrophosphate was also investigated. Receptor **4** was the only receptor to show an isotherm indicative of relatively simple binding equilibria, which could be fitted to a 1:1 model (one receptor **4**:one pyrophosphate anion) (Table 1, *K*_a_ 4160 M^−1^). The other receptors underwent fast and slow exchange processes in solution with pyrophosphate (ESI Figure S55, S61, and S67) [17,24]. Receptors **1** and **2** exhibited complex equilibria in the presence of the bicarbonate anion, which may be due to proton transfer, as was observed previously with the tris phenyl thiourea [18,19]. The higher-order 1:2 binding model (one receptor:two bicarbonate anions) proved to be a better fit for receptors **3** and **4** (Table 1), in contrast to the 1:1 binding seen in the tris *tert*-butyl thiourea bicarbonate titration [19]. 

For chloride, the same 1:2 binding mode (one receptor: two chloride anions) was seen when chloride was titrated with receptors **1** and **2**, as with the analogous tripodal receptors previously reported by Busschaert et al. and Jowett et al. [16,18,19]. Despite the presence of an additional (thio)urea motif, the tetrapodal receptors displayed substantially lower affinities for chloride than the analogous tripodal receptors. 

The lower chloride-binding affinities exhibited by the tetrapodal scaffold compared to the tripodal receptors were explored using ^1^H-NMR dilution studies (ESI S5.2). Dilution studies or concentration-dependent ^1^H-NMR was conducted, which indicates the intermolecular association. From the data collected, a dimerization constant (*K*_d_) of 11,000 M^−1^ ± 1000 could be calculated. The crystal structure of **3** also indicates the formation of aggregates formed via thiourea hydrogen bonding interactions. Therefore, the lower chloride-binding affinities can be attributed to the formation of intermolecular (thio)urea⋯(thio)urea hydrogen bonds in the tetrapodal receptors, which compete with anion binding [25].

### 2.4. Transport Studies

#### 2.4.1. Chloride/Nitrate Exchange Assay

**Figure 4:**
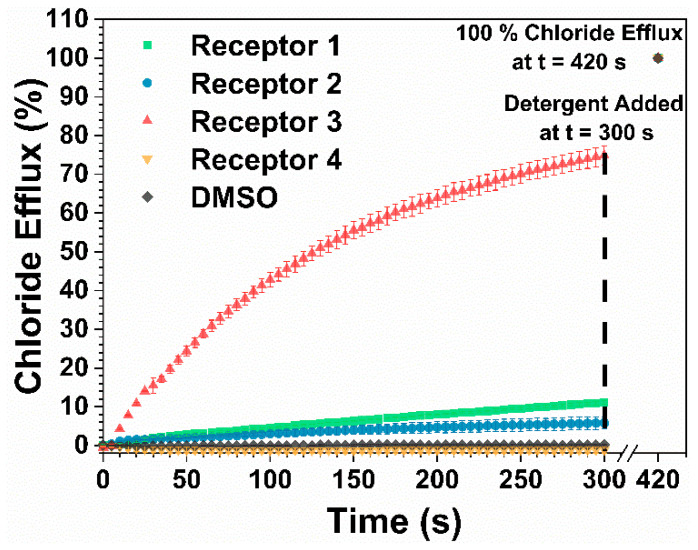
A comparison of the chloride efflux between receptors **1**–**4** when 1 mol%, with respect to the lipid concentration, was added to the medium.

The chloride/nitrate exchange assay was employed to study the ability of the receptors to facilitate the exchange of these anions across a lipid bilayer. Following the procedure previously reported by Jowett et al., unilamellar vesicles (200 nm) were prepared from 1-palmitoyl-2-oleoyl-sn-glycero-3-phosphocholine (POPC) and preloaded with an internal solution of sodium chloride (489 mM) in an external solution of sodium nitrate (489 mM), each buffered with a sodium phosphate salt buffer (5 mM) to pH 7.2 (see methods and materials Section 3.4.2) [26]. The chloride efflux promoted upon the addition of the receptors to the system was then measured by a chloride ion selective electrode (ISE). The efflux data were then fitted to the Hill equation to determine the concentration of the receptor that will promote half of the maximum observed chloride efflux (EC_50_) at 270 s. Initial tests were performed on each receptor at 1 mol% (Figure 4) and 10 mol% compared to the lipid concentration. 

Due to the relative inactivity of receptors **1** and **4**, no further tests were conducted on these compounds, and Hill analysis was not performed. Receptors **2** and **3** were found to have EC_50_ values of 3.92 and 0.408 mol%, respectively (Table 2).

The significant increase in the EC_50_ value of receptor **2** can be attributed to the lack of solubility of the receptor at higher concentrations. The initial rate constants at 1 mol% of the receptor (Table 2) support the findings that receptor **3** is the most active with an initial rate of 0.703 s^−1^, followed by receptors **1**, **2**, and **4** (ESI Table S5 and Figure S43). This data is also in agreement with cLogP values calculated using the ALOGPS 2.1 app [27,28]. The results show much higher lipophilicities of the thiourea receptors **1** and **3** (clogP = 4.11 and 3.32, respectively) than the urea receptors **1** and **4** (clogP = 3.09 and 1.65, respectively), which is consistent with the experimental trend of the anion transport activities. However, the cLogP values in the order of **1** > **3** > **2** > **4** does not entirely match the order of the reported anion transport activity (**3** > **1** > **2** > **4**). This is likely because the phenyl-containing receptors have lower deliverability (a higher tendency of aggregation, forming precipitates) when loaded as DMSO solutions due to the tendency of aromatic units to stack.

#### 2.4.2. Cationophore-Coupled Assay

**Figure 5:**
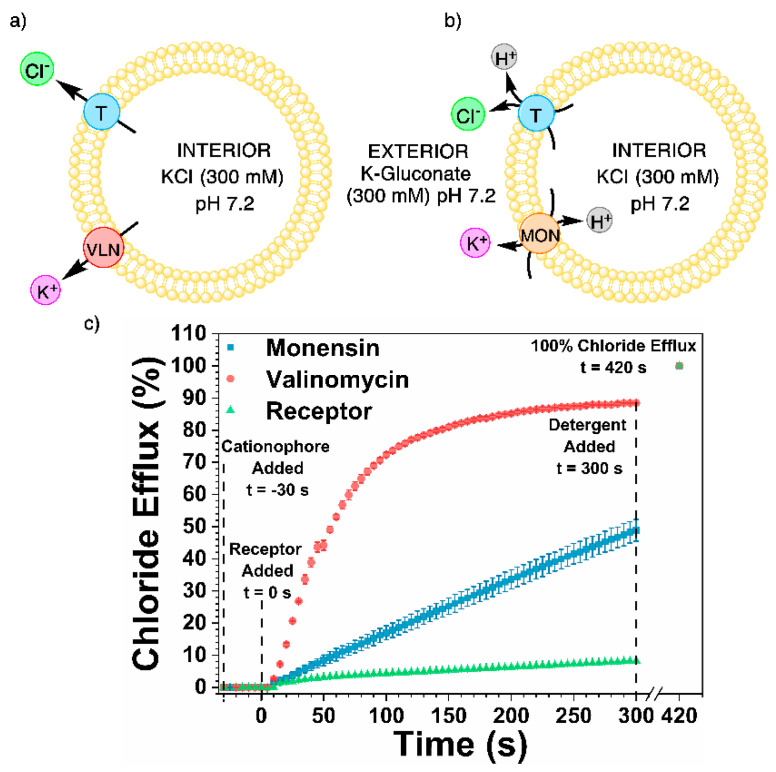
The cationophore-coupled assays used to determine the mechanism of transport facilitated by receptors **1**–**4** (T, blue). The internal (KCl, 300 mM) and external (K-Gluconate, 300 mM) solutions were buffered to pH 7.2 with 4-(2-hydroxyethyl)-1-piperazineethanesulfonic acid (HEPES) (5 mM). Two different conditions were tested, (**a**) the ion transport was seen in the presence of valinomycin (VLN, red), and (**b**) the ion flux in the presence of monensin (MON, orange). (**c**) The results of receptor **3** in the cationophore-coupled assay.

A cationophore-coupled assay was used to study the mechanism of anion transport. Vesicles (200 nm), preloaded with an internal solution of potassium chloride (300 mM), were suspended in an external solution of potassium gluconate (300 mM) and buffered to a pH of 7.2 with 4-(2-hydroxyethyl)-1-piperazineethanesulfonic acid (HEPES, 5 mM). Highly hydrophilic gluconate was used as the external anion to prevent anion exchange. The first assay was used to test electrogenic Cl^−^ uniport, facilitated by the anion transporters, where the natural cationophore valinomycin balances the electrical potential across the membrane caused by anion transport through K^+^ uniport (Figure 5a) [19,29]. The second assay was for electroneutral transport, Cl^−^/H^+^ symport facilitated by the anion transporters, where the natural cationophore monensin balances the electric potential over the membrane caused by Cl^−^/H^+^ cotransport via M^+^/H^+^ exchange (Figure 5b) [19,30]. 

Initial tests showed receptor **4** to be inactive, and further testing at higher concentrations was not possible due to solubility issues in DMSO. Receptor **2** exhibited only a slight increase in transport in both systems from the control receptor-only system. An increase in transport was also observed for the phenyl urea receptor **1**. The maximum chloride efflux achieved throughout the experiment was 19%. However, only a small disparity between the electrogenic and electroneutral modes of transport was seen, reinforced by the insignificant difference in the initial chloride efflux (Table 2 *k*_initial_ 0.070 and 0.028, s^−1^, respectively), showing that receptor **1** is not selective for chloride over proton transport.

Further analysis of these results can be performed by considering the electrogenic transport character from the initial transport rates. Calculating the ratio between the *k*_initial_ values of valinomycin and monensin provides an insight as to whether the electrogenic Cl^−^ uniport mechanism of transport is favored. If the ratio is < 1, then the electroneutral Cl^−^/H^+^ cotransport mechanism is more prevalent [18]. An electrogenic character that is ≈1 describes no selectivity of one mode of transport over the other, while an electrogenic character > 1 specifies electrogenic Cl^−^ uniport as the main transport mechanism [18]. An 11-fold increase in the transport rate facilitated by receptor **3** is seen in the initial rate of chloride transport. This is promoted by the electrogenic Cl^−^ uniport mechanism as compared to electroneutral H^+^/Cl^−^ cotransport (*k*_initial(MON)_ 0.168 compared to *k*_initial(VLN)_ 1.845 (s^−1^), Table 2). Therefore, the predominant mechanism of transport was electrogenic Cl^−^ uniport (Figure 5c). Jowett et al. also reported a comparable result for the structurally similar tren-based tripodal *tert*-butyl thiourea [18].

#### 2.4.3. 8-Hydroxypyrene-1,3,6-Trisulfonic Acid (HPTS) Transport Selectivity Assay

To further understand the selectivity of the anion transport mechanism between Cl^−^ and H^+^, HPTS vesicle transport studies were conducted according to three previously reported conditions [14,26]. The internal solution was 8-hydroxypyrene-1,3,6-trisulfonic acid (HPTS, 1 mM) and an *N*-methyl-d-glucamine salt chloride (NMDG-Cl, 100 mM), and the external solution was made from the same NMDG-Cl salt (100 mM). Both the internal and external solutions were buffered to pH 7.0 with HEPES (10 mM). A base pulse of NMDG-OH (5 mM) was added to create a pH gradient of pH~8 outside and pH 7 inside. The anion transporters facilitate Cl^−^/H^+^ cotransport (or OH^−^/Cl^−^ antiport) to dissipate the pH gradient, which can be measured by following the fluorescence change of the encapsulated HPTS. Concentration-dependent Hill analyses were performed to quantify the activities of the transporters in these assays.

**Figure 6:**
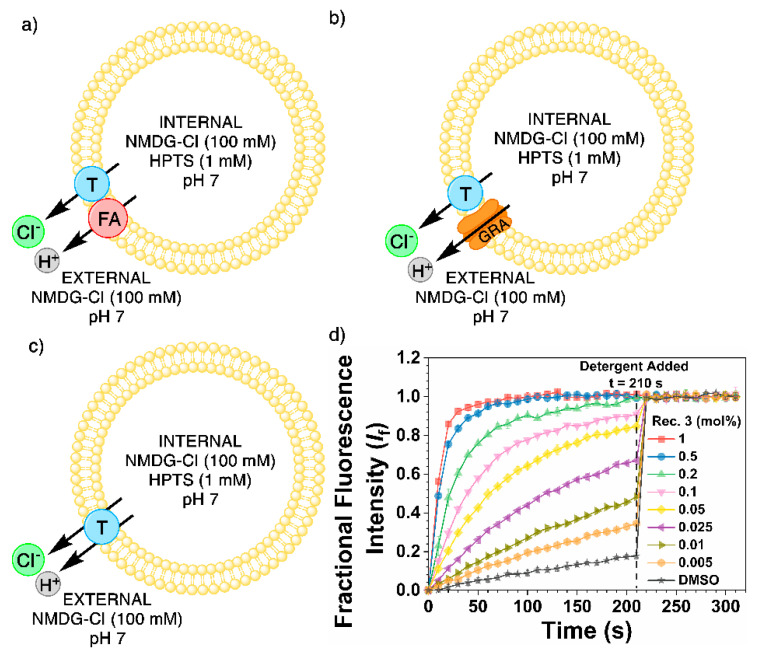
The three different tests utilized during the *N*-methyl-d-glucamine salt chloride (NMDG-Cl) 8-hydroxypyrene-1,3,6-trisulfonic acid (HPTS) transport selectivity assay. The vesicles (200 nm) had an internal solution of HPTS (1 mM) and NMDG-Cl (100 mM) and were in an external solution made from NMDG-Cl salt (100 mM). Both the internal and external solutions were buffered to a pH of 7.0 with HEPES (10 mM). (**a**) Receptors **1**–**4** (blue) were screened for the ability to undergo Cl^−^/H^+^ symport aided by fatty acids (red). (**b**) The vesicles were treated with gramicidin D (GRA, orange, 0.1 mol%) and tested for anion uniport. (**c**) Bovine serum albumin (BSA, 0.1 mol%) was used to sequester the fatty acids from the membrane to test receptor-facilitated Cl^−^/H^+^ symport. (**d**) The results of receptor **3** (Rec. **3**) during the NMDG-Cl transport assay in the presence of gramicidin D.

The receptors were screened for Cl^−^/H^+^ cotransport via potential fatty acid (FA) flip-flop in vesicles that contain ~1 mol% of free fatty acids (Figure 6a) and in vesicles treated with bovine serum albumin (BSA, 0.1%) to remove fatty acids from the membrane (Figure 6c) [14]. Finally, the receptors were screened for anion uniport with vesicles treated with the gramicidin D proton channel (GRA, 0.1 mol%) to accelerate H^+^ transport preventing this process from being rate-limiting [14]. Enhanced transport rate with gramicidin indicates Cl^−^ over H^+^ selectivity (Figure 6b).

In the HPTS assays, receptors **1**–**3** displayed reduced transport activity in BSA-treated vesicles compared with non-treated vesicles (Table 3, BSA). These results are indicative of a fatty acid-dependent H^+^ transport pathway where the anion transporter facilitates the transmembrane translocation of deprotonated fatty acids by binding to the carboxylate headgroup allowing fatty acids to complete an H^+^ transport cycle. No EC_50_ values could be determined for receptor **4** due to inactivity during all three of the transport conditions.

Significantly enhanced transport rates were observed for receptor **3** when the H^+^ channel gramicidin D was added, indicating that Cl^−^ uniport is much more efficient than Cl^−^/H^+^ symport facilitated by receptor **3** [18,31]. The selectivity of Cl^−^ uniport over Cl^−^/H^+^ symport (F_(selectivity)_) was quantified through the ratio of the EC_50_ values of the fatty acid test and the gramicidin D test. F > 1 indicates selectivity for Cl^−^ uniport over fatty acid-dependent Cl^−^/H^+^ symport. Remarkably, receptor **3** shows a high degree of selectivity toward Cl^−^ uniport with an F_(selectivity)_ value of 9.14 (Table 3). This is consistent with the result in the ISE assays, where receptor **3** largely facilitated electrogenic transport with the highest ratio between the initial rates of Cl^−^ uniport and Cl^−^/H^+^ symport determined by ISE (10.9, Table 2). Receptors **1** and **3** showed lower selectivity for Cl^−^ uniport in both ISE and HPTS assays, underscoring the importance of using bulky alkyl substituents, which presumably favors interactions with spherical Cl^−^ ions. When compared to the Cl^−^ uniport over Cl^−^/H^+^ symport selectivity determined in the same assay for the structurally similar tris *tert*-butyl thiourea (F_(selectivity)_ = 2.51), a 3.6 times improvement in Cl^−^ uniport selectivity was observed (Table 3) [18]. Although the transport activity of the tripodal receptor is higher than the tetrapodal receptor (presumably due to the lower deliverability of the large tetrapodal receptor), receptor **3** demonstrates an advantage of high Cl^−^ uniport selectivity seemingly due to its favorable interaction with spherical Cl^−^ ions over non-spherical, Y-shaped fatty acid anions [18]. Lending to the idea that a degree of chloride selectivity is achieved over fatty acid-dependent proton transport, the change in receptor design allows the disruption to the pH gradient to be minimized, rendering receptor **3** an excellent candidate for therapeutic and biophysical applications as a Cl^−^ ionophore where H^+^ transport should be avoided.

We also tested receptors **1**–**4** for sulfate and nitrate transport based on the HPTS NMDG-NO_3_ and NMDG_2_-SO_4_ assays, respectively. No sulfate transport activity was observed for any of the receptors presumably due to the highly hydrophilic nature of sulfate (ESI Figure S39–42) [18,32,33]. Vesicles containing NMDG-NO_3_ treated with gramicidin D were used to probe the receptors′ ability to facilitate nitrate transport. The collected data were fitted to the Hill equation to elucidate the EC_50_ values of nitrate transport. Thiourea receptors **1** and **3** showed higher activities of nitrate transport (0.11 and 0.033 mol%, respectively) than the less lipophilic receptors **2** and **4** (0.77 and 8.5 mol%, ESI Table S3). The EC_50_ values of receptors **1**–**3** in the HPTS NMDG-NO_3_ assay followed the general trend found in the chloride/nitrate exchange assay and the HPTS NMDG-Cl assay.

## 3. Materials and Methods 

### 3.1. General Experimental

#### 3.1.1. Chemicals and Consumables

All chemical reagents used in syntheses were obtained from commercial sources.

- N-tosylaziridine (CAS No: 3634-89-7) was purchased from Combi-Blocks, Inc, San Diego, CA, USA.
- Glacial acetic acid (CAS No: 64-19-7) was purchased from Thermo Fisher Scientific, North Ryde, NSW, Australia.
- 1-palmitoyl-2-oleoyl-sn-glycero-3-phosphocholine (POPC) (CAS No: 26853-31-6) was purchased from Avanti Polar Lipids, Inc, Alabaster, AL, USA.
- Monensin sodium salt (Mon) (CAS No: 22373-78-0) was purchased Cayman Chemical, Ann Arbor, MI, USA.

The following chemicals were all bought from Australia (Merk-Sigma, Castle Hill, NSW) or the United States of America (Merk-Sigma, St Louis, MO, USA).

- Bovine serum albumin (BSA) (CAS No: 9048-46-8)—USA.
- Valinomycin (Potassium ionophore I) (CAS No: 2001-95-8)—Australia.
- 4-(2-hydroxyethyl)-1-piperazineethanesulfonic acid (HEPES) (CAS No: 7365-45-9)—Australia.
- Gramicidin D (GRA) (CAS No: 1405-97-6)—Australia.
- *N*-methyl-*d*-glucamine (NMDG) (CAS No: 6284-40-8)—Australia.
- 8-Hydroxypyrene-1,3,6-Trisulfonic Acid (HPTS) (CAS No: 6358-69-6)—Australia.
- Ethylenediamine (CAS No: 107-15-3)—Australia.
- Hydrobromic acid (48%) (CAS No: 10035-10-6)—USA.
- Phenyl isocyanate (CAS No: 103-71-9)—Australia.
- Phenyl isothiocyanate (CAS No: 103-72-0)—Australia.
- *tert*-Butyl isocyanate (CAS No: 1609-86-5)—Australia.
- *tert*-Butyl isothiocyanate (CAS No: 590-42-1)—USA.

The purchased reagents did not undergo any further purification, and all solvents used in synthesis were anhydrous which were provided from the Innovative Technology PureSolv7 solvent purification system located in the University of Sydney in the School of Chemistry. The preparative thin layer chromatography (TLC) Silica Gel 60 F_245_ (1.0 mm) glass sheets (20 × 20 cm) used were also purchased from Merk-Sigma, Castle Hill, NSW, Australia.

#### 3.1.2. Investigation and Characterization

^1^H-NMR (300 or 400 MHz) and ^13^C-NMR (101 MHz) data were collected at room temperature on a Bruker Avance DRX300, a Bruker Avance DPX 400 or Bruker AVIII 500 MHz NMR spectrometer (Carnation, WA, USA) ^1^H-NMR (300 or 400 MHz) and ^13^C-NMR (101 MHz) data were collected at room temperature on a Bruker Avance DRX300, a Bruker Avance DPX 400 or Bruker AVIII 500 MHz NMR spectrometer. All chemical shifts (^1^H-NMR and ^13^C-NMR ppm) reported in (δ, ppm) relative to the residual deuterated solvent peaks of dimethyl sulfoxide, (CD)_3_SO (2.50, 39.7 ppm), acetone, C_3_D_6_O (2.05, 206.7 and 29.9 ppm) or methylene chloride, and CD_2_Cl_2_ (5.32, 54.0 ppm).

The chloride/nitrate and cationophore coupled exchange assay were conducted on the Fisherbrand™ Accumet™. Chloride Combination Electrode—Mercury-Free while the HPTS transport fluorescence data was recorded on an Agilent Cary Eclipse Fluorescence Spectrophotometer.

Mass spectrometry was performed at both low resolution (LS-MS) on a Bruker amazon SL mass spectrometer equipped with a quadrupole analyzer and high resolution (HR-MS) on a Bruker Solarix 2XR mass spectrometer. The technique used to record the mass spectrum experiments were negative electrospray ionization with spectrums recorded for both positive and negative electrospray ionization and relative intensity data recorded as *m*/*z*. Melting points were recorded on the METTLER TOLEDO MP50 melting point system, and data were reported as a range (°C).

### 3.2. Synthesis

#### 3.2.1. General Methods

All syntheses were performed at the University of Sydney in the School of Chemistry. All syntheses was performed under nitrogen at room temperature unless otherwise stated. Purification performed with preparative thin layer chromatography (TLC) used Sigma-Aldrich Silica Gel 60 F_245_ (1.0 mm) glass sheets (20 × 20 cm). The eluent mixtures used in preparative TLC purification have been reported in (*v*/*v*) ratios. 

#### 3.2.2. Synthesis of Previously Reported Compounds

Compound: *N*^1^,*N*^1^,*N*^2^,*N*^2^-Tetrakis(2-aminoethyl)-1,2-ethanediamine

The tetrakisamine HBr salt (1 g) was added to ethanol (100 mL) with stirring open to the air. Sodium hydroxide (1 M) was then added dropwise until the pH was approximately 8, which was allowed to stir for 1 h. The solution was then evaporated to form a solid mixture of sodium bromide (NaBr, white solid) and tetrakis amine (green oil). The solid mixture of NaBr and free amine was dried under vacuo for 5 h to ensure all residual water and ethanol had been removed. Characterization occurred during subsequent transporter reactions as this compound is unstable.

Yield: 0.42 g (100%); LR-MS (ESI^+^) [M + H]^+^: 233.1 *m*/*z*.

#### 3.2.3. Synthesis of Transporters **1**–**4**

Compound: 1,1′,1′′,1′′′-((Ethane-1,2-diylbis(azanetriyl))tetrakis(ethane-2,1-diyl))-tetrakis(3-phenylthiourea) (tetra PhS, **1**)

The tetraamine (0.25 g) was partially dissolved in dichloromethane (DCM, 40 mL) and allowed to stir for 20 min. An excess of phenyl isothiocyanate (571 µL, 4.4 equiv.) in DCM (10 mL) was added to the mixture dropwise and stirred for 10 min. The mixture was heated to r.t. and stirred for 48 h, after which the DCM was filtered off, washed with DCM (3 × 10 mL), and evaporated to complete dryness. The solidified green oil was then dissolved in chloroform (2 mL) and precipitated out with hexane to form a white solid that was filtered off, and dried under vacuo. Preparative TLC was then used first with chloroform:acetone (7:3) to separate the main product from the side products and then DCM:methanol (95:5) to purify the main product. The TLC silica was then stirred in chloroform:acetone (7:3) for 1 h, and the filtrate was collected, evaporated and dried in vacuo.

Yield: 0.84 g (66%); ^1^H NMR (400 MHz, DMSO-d_6_): δ 9.62 (s, 4H), 7.54 (s, 4H), 7.37 (d, *J* = 7.68 Hz, 8H), 7.32 (t, *J* = 7.81 Hz, 8H), 7.11 (t, *J* = 7.15 Hz, 4H), 3.53 (q, *J* = 5.75 Hz, 8H), 2.65 (t, *J* = 6.31 Hz, 8H), 2.57 (s, 4H), ^13^C NMR (101 MHz, CD_2_Cl_2_): δ 180.9 (s), 137.5 (s), 129.3 (s), 126.2 (s), 124.8 (s), 52.0 (s), 42.7 (s), 41.0 (s), 23.8 (s), LR-MS (ESI^+^): [M + H]^+^: 773.38 *m*/*z*, (ESI^−^) [M − H]^−^: 771.38 *m*/*z*, HR-MS (ESI^+^) calculated for C_38_H_48_N_10_S_4_ [M + H]^+^: 773.29798 *m*/*z*, found: 773.30190 *m*/*z*, MP: 102–106 °C.

Compound: 1,1′,1′′,1′′′-((Ethane-1,2-diylbis(azanetriyl))tetrakis(ethane-2,1-diyl))tetrakis(3-phenylourea) (tetra PhO, **2**)

The tetraamine (0.42 g) was partially dissolved in acetonitrile (50 mL). An excess of phenyl isocyanate (1 mL) in acetonitrile (50 mL) was added to the mixture dropwise and allowed to stir for 20 min. The mixture was heated to r.t. and allowed to stir for 72 h, after which the white precipitate was filtered off, washed with acetonitrile (3 × 10 mL), and evaporated to complete dryness. The precipitate was washed with distilled water (3 × 10 mL), hexane (3 × 10 mL), and then a mixture of ethyl acetate (25%) and hexane (75%) before being dried in vacuo.

Yield: 0.81 g (63%); ^1^H NMR (400 MHz, DMSO-d_6_): δ 8.052 (s, 4H), 7.36 (d, *J* = 7.84 Hz, 8H), 7.18 (t, *J* = 7.83 Hz, 8H), 6.87 (t, *J* = 7.32 Hz, 4H), 6.13 (t, *J* = 5.16 Hz, 4H), 3.18 (q, *J* = 5.92 Hz, 8H), 2.62 (s, 4H), 2.56 (t, *J* = 6.38 Hz, 8H), ^13^C NMR (126 MHz, DMSO-d_6_): δ 155.8 (s), 140.9 (s), 129.1 (s), 121.5 (s), 118.3 (s), 54.6 (s), 52.6 (s), 37.9 (s), LR-MS (ESI^+^): [M + H]^+^: 709.4 *m*/*z*, (ESI^−^) [M − H]^−^ and [M − 2H]^−^: 708.5 and 707.5 *m/z*, HR-MS (ESI^+^) calculated for C_38_H_48_N_10_O_4_ [M + H]^+^: 709.38935 *m*/*z*, found: 709.39328 *m*/*z*, MP: 214–217 °C.

Compound: 1,1′,1′′,1′′′-((Ethane-1,2-diylbis(azanetriyl))tetrakis(ethane-2,1-diyl))-tetrakis(3-(*tert*-butyl)thiourea) (tetra *t*-BuS, **3**)

The tetra free amine (0.25 g) was partially dissolved in DCM (40 mL) and allowed to stir for 20 min. An excess of *tert*-butyl isothiocyanate (502 µL) in DCM (10 mL) was added dropwise and stirred for 10 min. The mixture was heated to r.t. and stirred for 48 h, after which the DCM was filtered off, washed with DCM (3 × 10 mL), and evaporated to complete dryness. The solidified green oil was then dissolved in chloroform (2 mL) and precipitated with hexane to form a white solid that was filtered off, and dried under vacuo. Preparative TLC was then used first with chloroform:acetone (7:3) to separate the main product from the side products and then DCM:methanol (95:5) to purify the main product. The TLC silica was then stirred in chloroform:acetone (7:3) for 1 h, and the filtrate was collected, evaporated, and dried in vacuo.

Yield: 0.76 g (10%); ^1^H NMR (400 MHz, DMSO-d_6_): δ 7.17 (s, 4H), 7.07 (t, *J* = 4.91 Hz, 4H), 3.45 (q, *J* = 5.45 Hz, 8H), 2.61 (s, 4H), 2.58 (t, *J* = 6.53 Hz, 8H), 1.41 (s, 36H), ^13^C NMR (101 MHz, Acetone-d_6_): δ 181.8, 53.3, 52.7, 52.6, 52.5, 41.8, 29.4, 22.0, LR-MS (ESI^+^): [M + H]^+^: 693.49 *m*/*z*, (ESI^−^) [M + Cl]^−^: 727.49 *m*/*z*, HR-MS (ESI^+^) calculated for C_30_H_64_N_10_S_4_ [M + H]^+^: 693.42318 *m*/*z*, found: 693.47710 *m*/*z*, MP: 104–107 °C.

Compound: 1,1′,1′′,1′′′-((Ethane-1,2-diylbis(azanetriyl))tetrakis(ethane-2,1-diyl))-tetrakis(3-(*tert*-butyl)urea) (tetra *t*-BuO, **4**)

The tetra free amine (0.25 g) was partially dissolved in DCM (40 mL) and allowed to stir for 20 min. An excess of *tert*-butyl isocyanate (547 µL) in DCM (10 mL) was added dropwise and stirred for 10 min. The mixture was heated to r.t. and stirred for 48 h, after which the DCM was filtered off, washed with DCM (3 × 10 mL), and evaporated to complete dryness. The solidified green oil was then dissolved in chloroform (2 mL) and precipitated with hexane to form a white solid that was filtered off, and dried under vacuo. Preparative TLC was then used first with chloroform:acetone (7:3) to separate the main product from the side products and then DCM:methanol (95:5) to purify the main product. The TLC silica was then stirred in chloroform:acetone (7:3) for 1 h, and the filtrate was collected, evaporated and dried in vacuo.

Yield: 0.47 g (69%); ^1^H NMR (400 MHz, DMSO-d_6_): δ 5.72 (s, 4H), 5.69 (t, *J* = 5.49 Hz, 4H), 2.99 (q, *J* = 5.68 Hz, 8H), 2.46 (s, 4H), 2.41 (t, *J* = 6.03 Hz, 8H), 1.22 (s, 36H), ^13^C NMR (126 MHz, DMSO-d_6_): δ 158.1, 55.3, 53.2, 49.4, 37.8, 29.8, LR-MS (ESI^+^): [M + H]^+^: 629.6 *m*/*z*, (ESI^−^) [M − H]^−^, [M + Cl]^−^ and [M + Br]^−^: 627.6, 663.6 and 707.5 *m*/*z*, HR-MS (ESI^+^) calculated for C_30_H_64_N_10_O_4_ [M + H]^+^: 629.51456 *m*/*z*, found: 629.51848 *m*/*z*. MP: the compound decomposed before it melted.

### 3.3. ^1^H-NMR Binding Studies

#### 3.3.1. Anion-Binding Studies

^1^H-NMR titrations used in anion binding experiments were performed by pipetting aliquots of the guest anion from 0–4 equivalents into a receptor host solution. The receptor ′host′ stock solution (5 mM) was made by dissolving the dry receptor into DMSO-d_6_/0.5% water. Due to the TBA/TEA salts of the respective anions being hygroscopic they were first dried under vacuo for 24 h before used. The first concentrated ′guest′ solution (0.25 M) was made by dissolving the salt into the host solution and the second diluted guest solution (0.05 M) was made by diluting the first concentrated guest solution into more of the host solution. 

The host solution (0.5 mL) was added to a scrupulously clean and dry NMR tube, and an initial ^1^H-NMR spectrum was taken before the introduction of the guest anion. An aliquot of the dilute guest solution was then added to the NMR tube and mixed well to ensure a homogenous solution before another spectrum was taken. This was repeated with the dilute guest solution for a predetermined number of scans until two equivalents of guest. After this point, larger aliquots of the concentrated guest solution were added to four equivalents of guest. 

Each anion to be tested was tested twice per receptor. The spectra were calibrated to the deuterated solvent peak (DMSO-d_6_ = 2.50 ppm), and the shifts (ppm) of the protons that moved as the host and were binding to the guest were recorded. The data collected from the ^1^H-NMR titrations, the chemical shifts of the protons, were then subject to fitting to 1:1, 1:2, and 2:1 host:guest binding models using the online web-applet BindFit from http://supramolecular.org to produce the association constant (*K*_a_) [23].

#### 3.3.2. ^1^H-NMR Dilution Studies

DMSO-d_6_ (0.5 mL) solutions of the most active transporter, tetra *t*-BuS (**3**), were prepared to be tested via ^1^H-NMR for a dilution study. The transporter concentrations tested were 100 mM, 30 mM, 10 mM, 3 mM, 1 mM, 500 µM, 200 µM and 100 µM. Concentrations 100, 30, 10 and 1 mM were scanned sixteen times, 500 µM was scanned sixty-four times while the 200 µM and the 100 µM solutions were scanned two hundred and fifty-six times.

After collecting and correcting the ^1^H-NMR data to the DMSO-d_6_ solvent peak (2.50 ppm), the NH1 and NH2 peak positions were recorded in an excel document alongside the respective concentration they were collected at. This document was processed through the online web-applet BindFit from http://supramolecular.org to produce the dimerization constant (*K*_d_) using the Nelder-Mead method of fitting [23].

### 3.4. Transport Studies

#### 3.4.1. Vesicle Preparation

The paper “Supramolecular methods: the chloride/nitrate transmembrane exchange assay” by Laura A. Jowett and Philip A. Gale provided the method to prepare the synthetic vesicles [26]. A POPC stock solution (37.5 mM, 1 g) in chloroform (10 mL) was made, and a predetermined milliliter volume pipetted out. The volume of chloroform was slowly reduced to produce an even lipid film. The dried film was kept under vacuo for 12 h and rehydrated with an assay-specific internal solution in a ratio of 1:1 (1 mL of initial POPC stock solution to 1 mL of the desired internal solution). Rehydration took place by adding the desired internal solution to the dried lipid film and vortexing to produce a homogeneous lipid suspension. The suspension then underwent nine freeze-thaw (alternating from freezing in liquid nitrogen to warming to room temperature by thawing in water) cycles to produce the desired unilamellar vesicles. The suspension was allowed to sit at room temperature for 30 min before undergoing extrusion through a polycarbonate membrane (200 nm) twenty-five times. The resulting solution of lipids then underwent dialysis with the assay-specific external solution to remove any unencapsulated excess of the chosen internal solution.

#### 3.4.2. Chloride/Nitrate Exchange Assay

Vesicles were prepared using the method outlined in Section 3.4.1 Vesicle Preparation, with the internal solution being sodium chloride (489 mM) with a sodium phosphate salt buffer (anhydrous disodium hydrogen phosphate (5 mM) and sodium dihydrogen phosphate dihydrate (5 mM) buffered to pH 7.2). The chosen external solution was sodium nitrate (489 mM) with the same buffer described above. A stock solution of the receptor in DMSO (50 mM) was prepared and diluted for subsequent experiments, and the solution to be tested was prepared by diluting the prepared vesicle solution (1 mM) into the external solution (5 mL). The receptor solution (10 μL) was added to the test solution at 0 s, and any difference in chloride concentration was monitored. At 300 s, the vesicles in the solution were lysed with a Triton X-100 detergent solution (50 μL). At 420 s the 100% chloride efflux reading was taken to calibrate the chloride efflux obtained before this point.

#### 3.4.3. Cationophore-Coupled Assay

The method outlined in Section 3.4.1 Vesicle Preparation, was used to make synthetic vesicles with an internal solution of potassium chloride (300 mM) and an external solution of potassium gluconate (300 mM). Both the internal and external solutions were buffered to pH 7.2 with 4-(2-hydroxyethyl)-1-piperazineethanesulfonic acid (HEPES, 5 mM). To rid the unilamellar vesicles of any unencapsulated internal solution, and rather than undergoing dialysis, the vesicles underwent size-exclusion through a Sephadex G-25 column instead. The column was run with the external solution to afford a lipid stock solution (5 mL) of a known concentration. The lipid stock solution was diluted into the external solution to make the solution to be tested (1 mM, 5 mL). Two solutions of valinomycin and monensin in DMSO (0.1 mol% in relation to the lipid stock concentration) were made. Either the valinomycin or the monensin solution was added (10 μL) to the test solution—30 s before the start of the experiment; at 0 s, the transporter (10 μL) was added, and the chloride efflux was monitored until 240 s where the vesicles were lysed with a Triton X-100 detergent solution (50 μL) and the final chloride efflux reading was taken at 300 s.

#### 3.4.4. 8-Hydroxypyrene-1,3,6-Trisulfonic Acid (HPTS) Transport Selectivity Assay

The vesicles used in these experiments were prepared with the method found in Section 3.4.1 Vesicle Preparation and dialyzed with the method in Section 3.4.3 Cationophore Coupled Transport Assay. The internal solution was 8-hydroxypyrene-1,3,6-trisulfonic acid (HPTS, 1 mM) and an N-methyl-d-glucamine salt of the anion (X) to be studied (NMDG-X, 100 mM). The external solution was made from the same NMDG-X salt (100 mM), and both the internal and external solutions were buffered to a pH of 7.0 with HEPES (10 mM). The solution to be tested was made by diluting the lipid stock in the external solution (0.1 mM, 2.5 mL). Transport tests in normal vesicle conditions (in the presence of fatty acids (FA)) were started by adding an NMDG base pulse (500 nM, 25 μL) and then the transporter DMSO solution (5 μL) to the test solution at 0 s. At 280 s, the vesicles were lysed with Triton X-100 detergent solution (50 μL), and the final chloride efflux value was recorded at 320 s. The experiment can also be run in the presence of Gramicidin D (GRA, 0.1 mol%) in DMSO and is added at 0 s before the receptor is added to the test solution. The third condition of this experiment is performed when all fatty acids have been sequestered from the vesicle membranes by adding bovine serum albumin (BSA, 0.1 mol%) to the vesicles before the test starts.

#### 3.4.5. Hill Analysis

During both the Cl^−^/NO_3_^−^ transport assay and the cationophore transport assay, the chloride efflux (%, y) at 270 s was plotted against the calculated transporter concentration (mol%, x) and fit to the Hill equation (Equation (1)). The same was done during the transport studies, either 200 for NMDG-Cl or 260 s for NMDG-NO_3_, with y being the fractional fluorescence Intensity (*I*_f_).

(1) $$y=y0+\left(y1-y0\right)\frac{x^{n}}{\left(k^{n}+x^{n}\right)}$$

Equation (1): The Hill equation used to perform Hill analysis.

Where y1 is the maximum *I*_f_ or chloride efflux (%) value, y0 is the minimum chloride efflux (%) or *I*_f_ for a blank in DMSO, y is the chloride efflux or *I*_f_ at the specified time before detergent was added, and x is the concentration of the transporter (mol% in relation to the lipid concentration). Both *k* and *n* are variables to be found, where *k* is the concentration of the transporter, which promotes 50% chloride efflux (EC_50_, mol%), and *n* is the Hill coefficient.

#### 3.4.6. Initial Rate

All data analysis and fitting were performed in *OriginPro 9.7*. The initial rate of chloride efflux (*k*_initial_) was found first by fitting the chloride efflux as a percentage (%, y) against time (s, x) in an exponential decay equation (Equation (2)) until convergence.

(2) $$y=y0+A_{1}e^{\left(\frac{-x}{t_{1}}\right)}+A_{2}e^{\left(\frac{-x}{t_{2}}\right)}$$

Equation (2): The two-phase exponential decay equation was used to fit the chloride efflux data (%) against time (s) with time constants.

Where y0 is the offset from the curve, both *t*_1_ and *t*_2_ are time constants, and both *A*_1_ and *A*_2_ are amplitude contents. The fitted data were exported to Microsoft Excel, where a simple function (Equation (3)) was used to calculate the *k*_initial_ value.

(3) $$k_{initial}=\left(\frac{dy}{dx}\right)x=\;0=-\;\frac{A_{1}}{t_{1}}-\frac{A_{2}}{t_{2}}$$

Equation (3): The function used to calculate the *k*_initial_ value from the derived amplitude and time constants of each fitted data set.

Where there was poor convergence, seen in the divergence of the fitted R^2^ value from 1, the data set was fit using the initial regression equation (Equation (4)).

(4) y=a+bx

Equation (4): The linear regression equation used to fit data where slow transport was observed. Where y is the chloride efflux (%), x is time (s), and *k*_initial_ is given by the calculated slope *b*.

#### 3.4.7. The Partition Coefficient

The online ALOGPS 2.1 app was used to estimate the LogP values of the four tetrapodal transporters [27,28].

### 3.5. Single Crystal X-ray Diffraction

X-ray diffraction data were collected at 150 K on a SuperNova Dual, Cu, Atlas diffractometer at a wavelength of 1.54184 Å. The data collection and integration were performed within the XDS [34] software program. The solutions were obtained by direct methods using SHELXT [35] followed by successive refinements using full-matrix least-squares method against *F*^2^ using SHELXL-2018/3 [36]. The program OLEX2 [37] was used as a graphical SHELX interface.

## 4. Conclusions

The design and synthesis of anion transporters with improved selectivity are challenging but important in developing possible treatments for channelopathies such as cystic fibrosis. Reported here is the successful synthesis of novel tetrapodal anion transporters that have been subject to anion binding and transport studies to elucidate their properties. Although there has been an increase in the number of hydrogen bond donors going from the tripodal to the tetrapodal receptor scaffold, lower binding affinities to chloride, bicarbonate, sulfate, and dihydrogen phosphate were observed when compared to the tren-based receptors likely due to competitive receptor aggregation.

The results of the transport studies show that like the tripodal receptors, the tetrapodal receptors **1**–**3** followed the trend of being able to elicit some electroneutral Cl^−^/H^+^ symport due to a fatty acid-dependent H^+^ transport pathway. Nevertheless, the dominant mode of transport is electrogenic chloride uniport, as exhibited by receptors **1** and **3**. Although a decrease in transport activity was observed by changing the receptor scaffold from a tripod to a tetrapod, a marked increase in Cl^−^ over H^+^ selectivity was achieved. Therefore, through the increased encapsulation of the binding site, we have successfully reduced undesirable protonophoric activity through an increase in transport selectivity, which is made possible via increased encapsulation of spherical Cl^−^ ions and has improved the selectivity over transporting fatty acid anions by interacting with the carboxylate headgroups.

## Figures and Tables

**Table 1:** The complexation binding constants *K*_a_ (M**^−^**^1^) of receptors **1**–**4** to different anions determined in ^1^H-NMR binding studies performed in DMSO-d_6_/0.5% water. Anions were added as tetrabutylammonium salts (except in the case of bicarbonate which was added as the tetraethylammonium salt).

Receptor	*K*_a_ Cl^−^	*K*_a_ HCO_3_^−^	*K*_a_ SO_4_^2−^	*K*_a_ HP_2_O_7_^3−^
**1**	*K*_11_: 458 *K*_12_: 30	^1^	*K*_11_: 1000 *K*_21_: 56	^1^
**2**	*K*_11_: 447 *K*_12_: 28	^1^	^1^	^2^
**3**	*K*_11_: 241 *K*_12_: 16	*K*_11_: 1800 *K*_12_: 305	*K*_a_ > 10^4^	^3^
**4**	*K*_11_: 305 *K*_12_: 7	*K*_11_: 559 *K*_12_: 21	^1^	4160

The binding constants *K*_11_, *K*_12,_ and *K*_21_ are reported in the format *K*_receptor:anion_. ^1^ Complex binding and deprotonation of the receptor occurred. ^2^ Fast and slow exchange occurred. ^3^ The experimental data could not be fit to 1:1, 1:2, or 2:1 receptor:anion binding models.

**Table 2:** The initial transport rates of electrogenic and electroneutral transport of receptors **1**–**4**.

Receptor	Chloride/Nitrate Exchange Assay		Cationophore Coupled Assay *k*_initial_ (s^−1^)		Ratio ^3^	cLogP ^6^
	1 mol% *k*_initial_ (s^−1^) ^1^	EC_50_ (mol%) ^2^	*k*_initial(VLN)_^1^	*k*_initial(MON)_^1^		
**1**	0.074	n.d. ^4^	0.070	0.028	2.5	4.1
**2**	0.020	3.92	0.087	0.130	0.67	3.1
**3**	0.703	0.408	1.85	0.168	11	3.3
**4**	0.0	n.d. ^4^	n.d. ^5^	n.d. ^5^	n.d. ^4,5^	1.6

^1^ The initial Cl^−^ efflux when the receptors are added (*k*_initial_ (s^−1^)). ^2^ The EC_50_ values (mol%) of the receptors in the chloride/nitrate exchange assay. ^3^ The ratio of electrogenic transport character was performed via division of the valinomycin (VLN) test initial rate by the monensin (MON) test initial rate. ^4^ Not determined (n.d.) due to insufficient activity of receptor **4** (ESI Figure S19). ^5^ Inactivity of receptor **4** (ESI Figure S49) prevented further analysis. ^6^ The calculated partition coefficients were obtained using ALOGPS 2.1 via the Virtual Computational Chemistry Laboratory website [27,28].

**Table 3:** The EC_50_ and enhancement factors (F) of the tetrapodal transporters were calculated from experimental data collected from the HPTS NMDG-Cl transport selectivity assay and comparison tripodal transport values.

Tetrapodal Transporters	EC_50_ (mol%) ^1^			F_(selectivity)_^5^
	FA ^2^	Gra ^3^	BSA ^4^	
**1**	0.26	0.32	0.72	0.81
**2**	1.94	1.63	2.23	1.19
**3**	0.17	0.019	0.60	9.14
**4**	n.d. ^6^	n.d. ^6^	n.d. ^6^	n.d. ^6^
**Tripodal Transporters**				
Tris PhS ^7^	0.0081 ^8^	0.0021 ^8^	0.21 ^8^	3.86 ^8^
Tris PhO ^7^	0.78 ^8^	0.3 ^8^	9.8 ^8^	2.60 ^8^
Tris *t*-BuS ^7^	0.012 ^9^	0.0048 ^9^	0.29 ^9^	2.51 ^9^

^1^ The concentration of the transporter, which promotes 50% of the maximum chloride efflux (EC_50_, mol%) at 200 s into the experiment. ^2–4^ The EC_50_ values of the non-treated vesicles with fatty-acid impurities (FA) in the membrane, the vesicles treated with the proton channel gramicidin D (0.1 mol%, Gra), and the vesicles treated with bovine serum albumin (BSA). ^5^ The enhancement factor of Cl^−^ uniport over Cl^−^/H^+^ cotransport. ^6^ Not determined due to insufficient activity (n.d.). ^7^ Tripodal transporters have been abbreviated according to phenyl (Ph) or *tert*-Butyl (*t*-Bu) group and thiourea (S) or urea group (O). ^8^ Previously published data [18]. ^9^ Previously published data [19].

## Supplementary Materials

General procedures, synthesis of compounds **1**–**4**, characterization, crystallography, transport studies, and anion-binding experiments are available online.
