# Supplementary material for: Tetrapodal Anion Transporters
Source: Molecules. 2020 Nov 6;25(21):5179. doi: 10.3390/molecules25215179 (PMC7664440; doi:10.3390/molecules25215179)
Supplement: Supplementary file 1 [file molecules-25-05179-s001.pdf]

Supporting Information for
Tetrapodal Anion Transporters

Alexander M. Gilchrist, LiJun Chen, Xin Wu, William Lewis, Ethan N.W. Howe, Lauren K. Macreadie and Philip A. Gale*

Table of Contents:

S1. General Experimental: 2

S2. Transporter Synthesis and Characterisation: 3

S3. ^{1}H and ^{13}C-NMR Analysis of Tetrapodal Transporters: 5

S4. Transport Studies: 10

S4.1 General Experimental Procedures: 10

S4.2 Cl^{-}/NO_3^{-} Transport Exchange Assay: 11

S4.3 Cationophore Coupled Transport Assay: 14

S4.4 HPTS Transport Selectivity Assay: 16

S4.5 Structure-Activity Relationship: 35

S5. ^{1}H-NMR Binding Studies: 41

S5.1 Anion Binding Studies: 41

S5.2 ^{1}H-NMR Dilution Studies: 66

S6. Single crystal X-ray diffraction: 68

S7. References: 70

S1. General Experimental:

All syntheses were performed at the University of Sydney in the School of Chemistry. All chemical reagents used in synthesis were obtained from commercial sources. All solvents used in synthesis were anhydrous, which were provided from the Innovative Technology PureSolv7 solvent purification system. All syntheses were performed under nitrogen at room temperature unless otherwise stated. Purification performed with preparative thin layer chromatography (TLC) used Sigma-Aldrich Silica Gel 60 F_{245} (1.0 mm) glass sheets (20 × 20 cm). The eluent mixtures used in preparative TLC purification have been reported in (v/v) ratios.

^{1}H-NMR (300 or 400 MHz) and ^{13}C-NMR (101 MHz) data were collected at room temperature on a Bruker Avance DRX300, a Bruker Avance DPX 400, or Bruker AVIII 500 MHz NMR spectrometer. All chemical shifts (^{1}H-NMR and ^{13}C-NMR ppm) reported in (δ, ppm) relative to the residual deuterated solvent peaks of dimethyl sulfoxide, $(CD)_3SO$ (2.50, 39.7 ppm), acetone, C_3D_6O (2.05, 206.7 and 29.9 ppm) or methylene chloride, and CD_2Cl_2 (5.32, 54.0 ppm).

Multiplicities observed in the NMR experiments were reported as either singlet (s), doublet (d), triplet (t), quartet (q), doublet of doublets (dd), multiplet (m), or broad (br) with J coupling constants (*J*) reported in Hertz (Hz). ^{1}H-NMR titrations used in anion binding experiments were performed by pipetting aliquots of the guest anion from 0-4 equivalents into a receptor host solution. The guest anion was dissolved in DMSO-d_6/0.5% water as the tetrabutylammonium (TBA) salt or tetraethylammonium (TEA) salt while the host receptor was dissolved in DMSO-d_6/0.5% water.

The 1-palmitoyl-2-oleoyl-sn-glycero-3-phosphocholine (POPC) lipids used in transport studies were supplied by Avanti Polar Lipids. Fluorescence data were recorded on an Agilent Cary Eclipse Fluorescence Spectrophotometer. Mass spectrometry was performed at both low resolution (LS-MS) on a Bruker amazon SL mass spectrometer equipped with a quadrupole analyser and high resolution (HR-MS) on a Bruker Solarix 2XR mass spectrometer. The technique used to record the mass spectrum experiments were negative electrospray ionization (ESI) with spectrums recorded for both positive and negative ESI and relative intensity data recorded as m/z. Melting points were recorded on the METTLER TOLEDO MP50 melting point system, and data was reported as a range (°C). All transporters have been referred to in an abbreviated form with the prefix 'tetra' and the suffix according to the phenyl or *tert*-butyl functional group, 'Ph' or '*t*-Bu', and either the (thio)urea group 'S' or 'O'. The synthesized receptors have also been allocated the numbers **1**–**4**.

S2. Transporter Synthesis and Characterization:

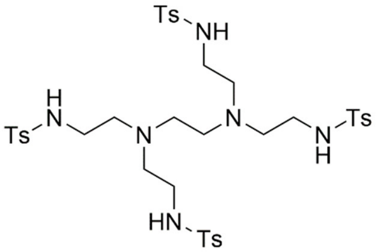

Figure S1. Structure of *N*,*N*',*N*'',*N*'''-[1,2-ethanediylbis(nitrilodi-2,1-ethanediyl)]tetrakis4-methylbenzenesulfonamide].

Following a previously reported method by Mansouji *et al.*, *N*-tosylaziridine (7 g, 0.03548 mol) was then dissolved in toluene (15 mL) and stirred with cooling.[1] A solution of ethylenediamine (426.5 mg, 422.3 μL, 0.00709 mol) and acetonitrile (15 mL) was added dropwise to the *N*-tosylaziridine solution for 30 minutes. The reaction mixture was allowed to stir for 3 hours at room temperature. The mixture was then heated to reflux at 65 °C for 48 hours after which it was allowed to cool. During cooling a white precipitate had formed which was collected and washed with acetonitrile (3 x 5 mL) and dried in *vacuo*.

Yield: 5.53 g (92%); **^{1}H-NMR** (400 MHz, DMSO-d_6): δ 7.65 (d, J = 8.2 Hz, 2H), 7.35 (m, J = 8.06 Hz, 3H), 2.64 (t, J = 6.5 Hz, 2H), 2.36 (s, 3H), 2.23 (t, J = 6.5 Hz, 2H), 2.10 (s, 1H), **LR-MS** (ESI$^+$) [M+H]$^+$: 849.11 *m/z*, (ESI$^-$) [M-2H]$^-$: 847.3 *m/z*.

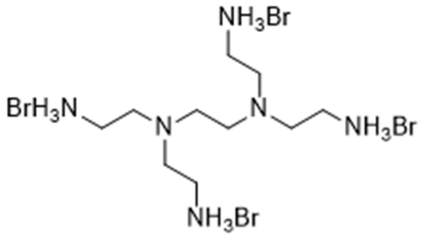

Figure S2. Structure of N^1,N^1,N^2,N^2-tetrakis(2-(bromo-λ^5-azaneyl)ethyl)ethane-1,2-diamine.

Following a previously reported method by Keypour *et al.*, tetrakistosylsulfonamide (4.0 g) was dissolved in a solution of aqueous hydrobromic acid (48%, 60 mL) and glacial acetic acid (40 mL).[2] The mixture was refluxed for 48 hours at 117 C after which it was allowed to cool before being placed in an ice bath. The resulting precipitate was washed with methanol (3 × 10 mL) and dried in *vacuo*.

Yield: 0.27 g (89%); **^{1}H NMR (**400 MHz, DMSO-d_6): δ 7.96 (s, 1H), 5.38 (br, 1H), 3.03 (s, 1H), **LR-MS** (ESI$^-$): [M+H]$^-$ and [M+Br]$^-$: 553.0 and 585.8 *m/z*.

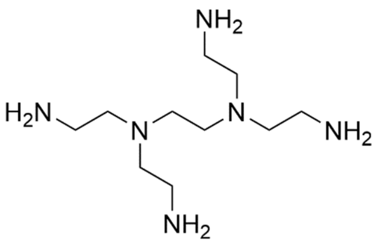

Figure S3. *N*[1],*N*[1],*N*[2],*N*[2]-tetrakis(2-aminoethyl)-1,2-ethanediamine

The tetrakisamine HBr salt (1 g) was added to ethanol (100 mL) with stirring open to the air. Sodium hydroxide (1 M) was then added dropwise until the pH was approximately 8 which was allowed to stir for 1 hour. The solution was then evaporated to form a solid mixture of sodium bromide (NaBr, white solid) and tetrakis free amine (green oil). The solid mixture of NaBr and free amine was dried under *vacuo* for 5 hours to ensure all residual water and ethanol had been removed. Characterization occurred during subsequent transporter reactions as this compound is unstable.

Yield: 0.42 g (100%); **LR-MS** (ESI^+) $[M+H]^+$: 233.1 *m/z*.

S3. ^{1}H and ^{13}C-NMR Analysis of Tetrapodal Transporters:

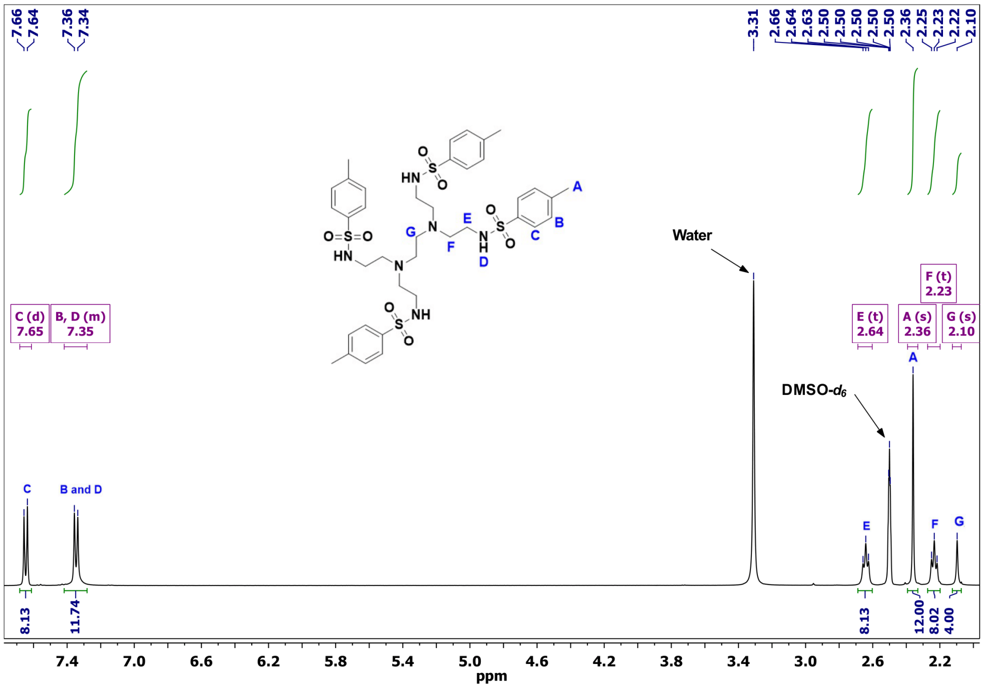

Figure S4: ^{1}H-NMR (400 MHz) spectrum of tetrakistosylsulfonamide in DMSO-d_6 at 298 K. The NH group, has undergone an upfield shift causing an overlap with proton B.

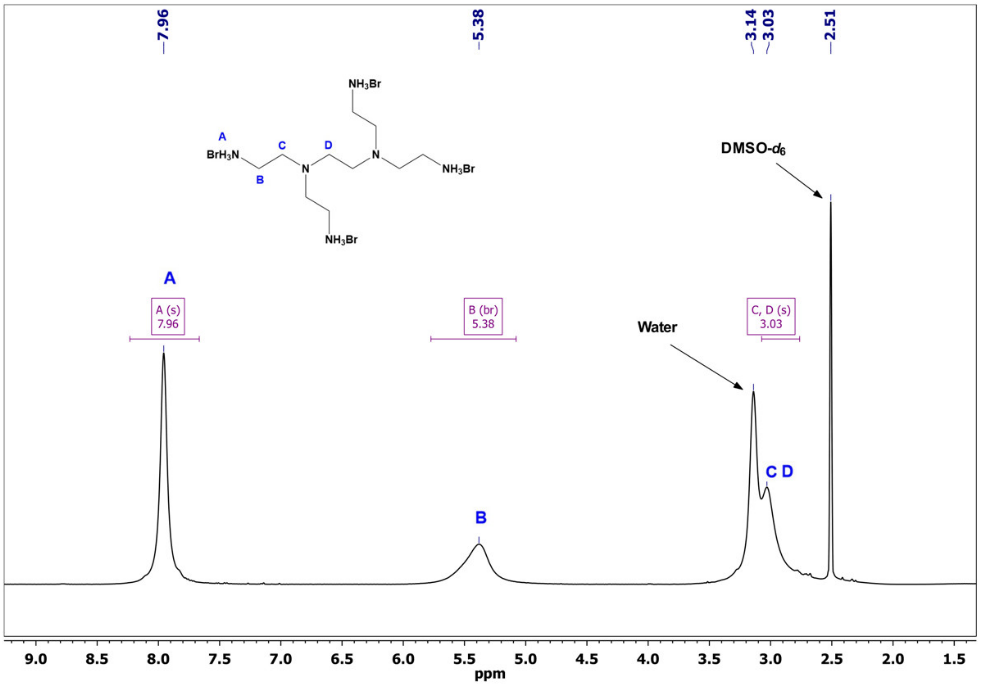

Figure S5: ^{1}H-NMR (400 MHz) spectrum of tetraamine HBr salt in DMSO-d_6 at 298 K. Here the two CH_2 (C and D) peaks have undergone broadening and overlapped with water.

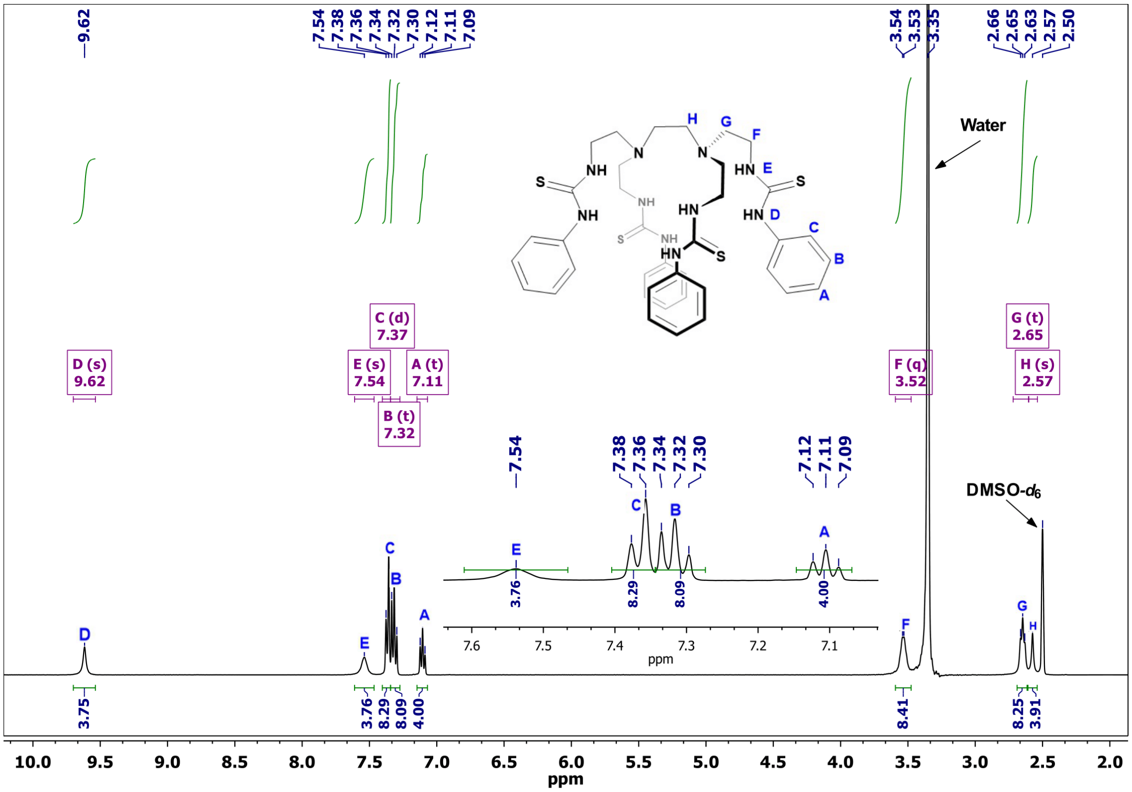

Figure S6: ^{1}H-NMR (400 MHz) spectrum of tetraphenylthiourea (**1**) in DMSO-d_6 at 298 K.

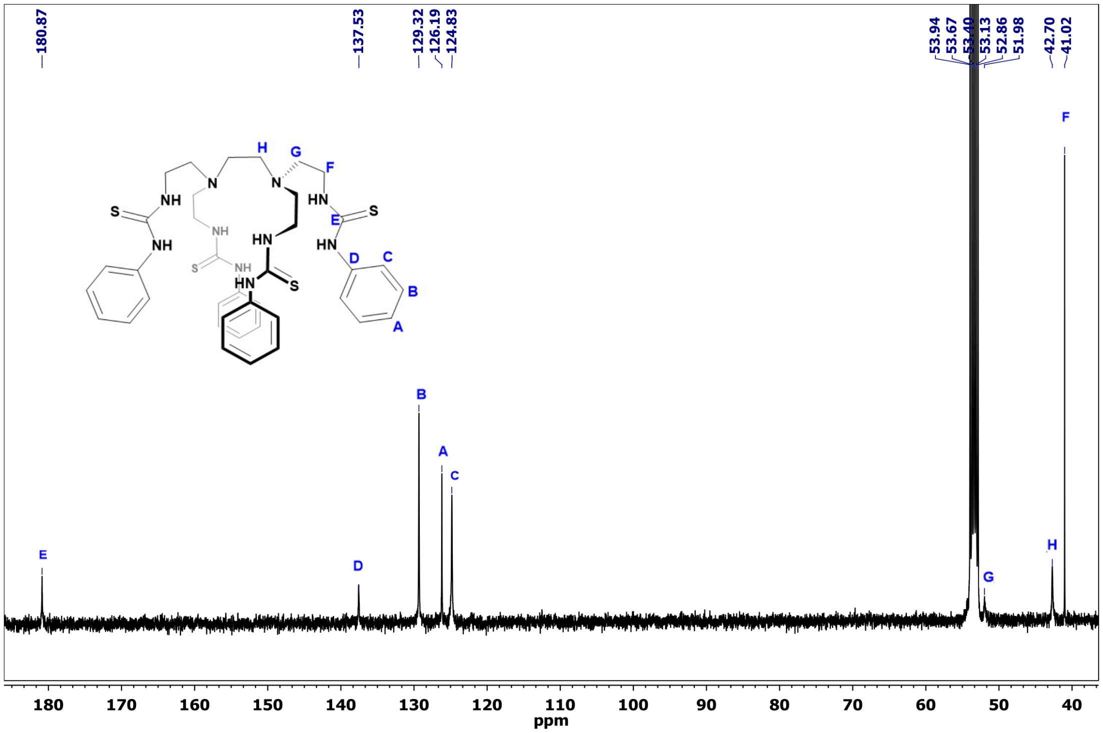

Figure S7: ^{13}C-NMR (101 MHz) spectrum of tetraphenylthiourea (**1**) in CD_2Cl_2 at 298 K.

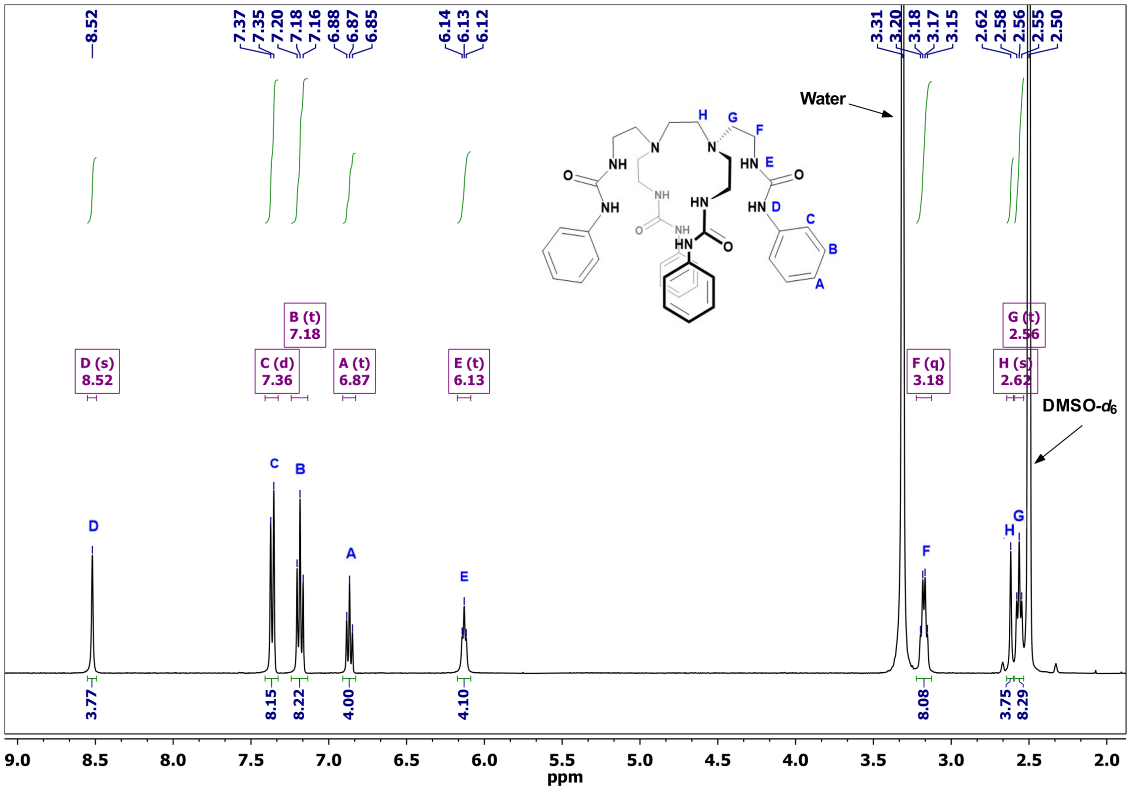

Figure S8: ^{1}H-NMR (400 MHz) spectrum of tetraphenylurea (**2**) in DMSO-d_6 at 298 K.

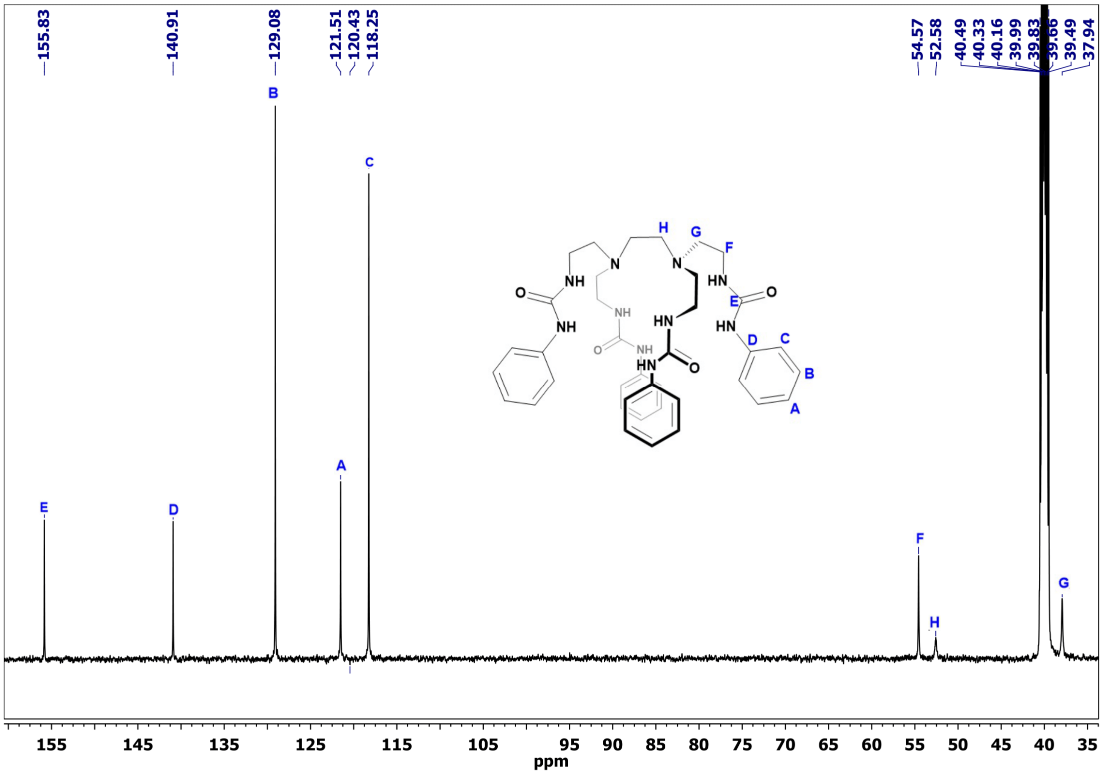

Figure S9: ^{13}C-NMR (126 MHz) spectrum of tetraphenylurea (**2**) in DMSO-d_6 at 298 K.

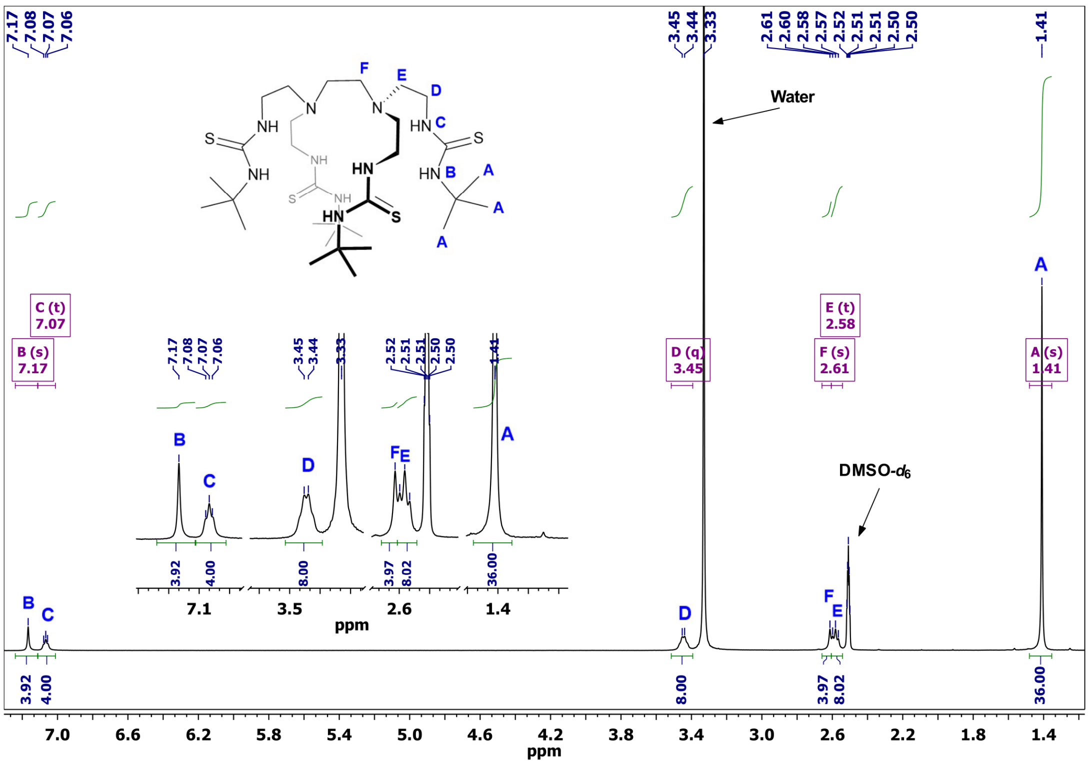

Figure S10: ^{1}H-NMR (400 MHz) spectrum of tetra *tert*-butylthiourea (**3**) in DMSO-d_6 at 298 K.

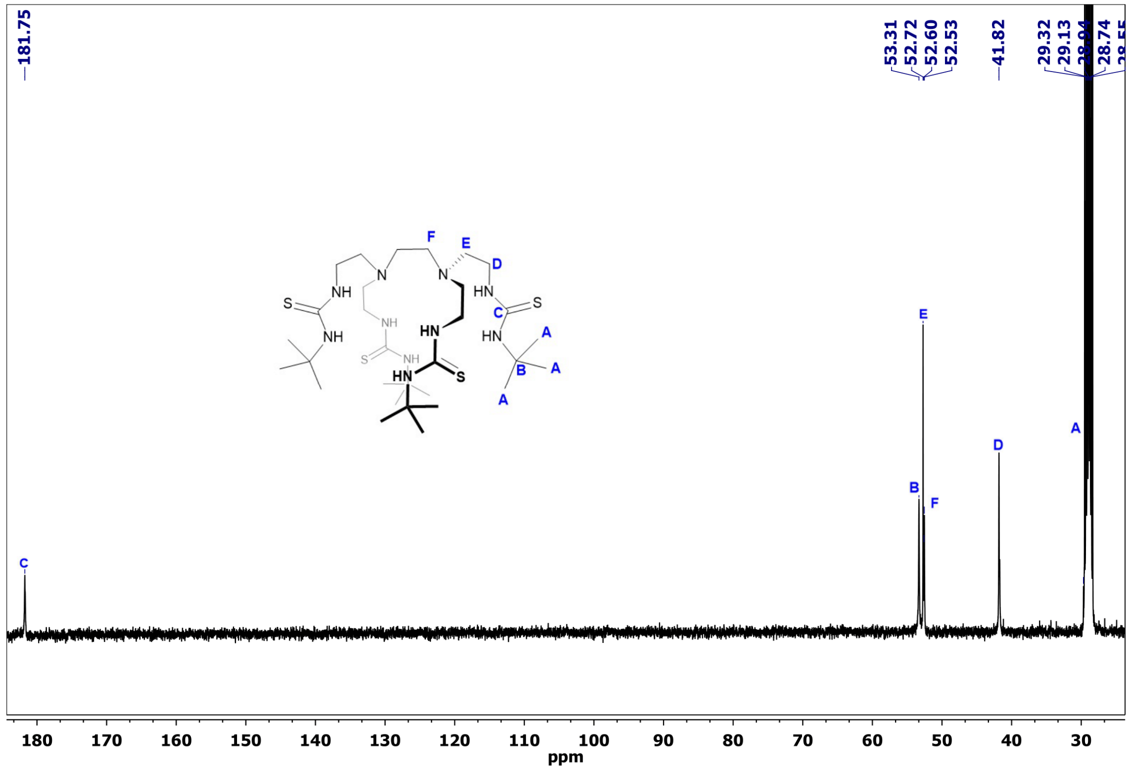

Figure S11: ^{13}C-NMR (101 MHz) spectrum of tetra *tert*-butylthiourea (**3**) in Acetone-d_6 at 298 K. Note: the Acetone-d_6 solvent peak has overlapped with the carbon 'A' peak.

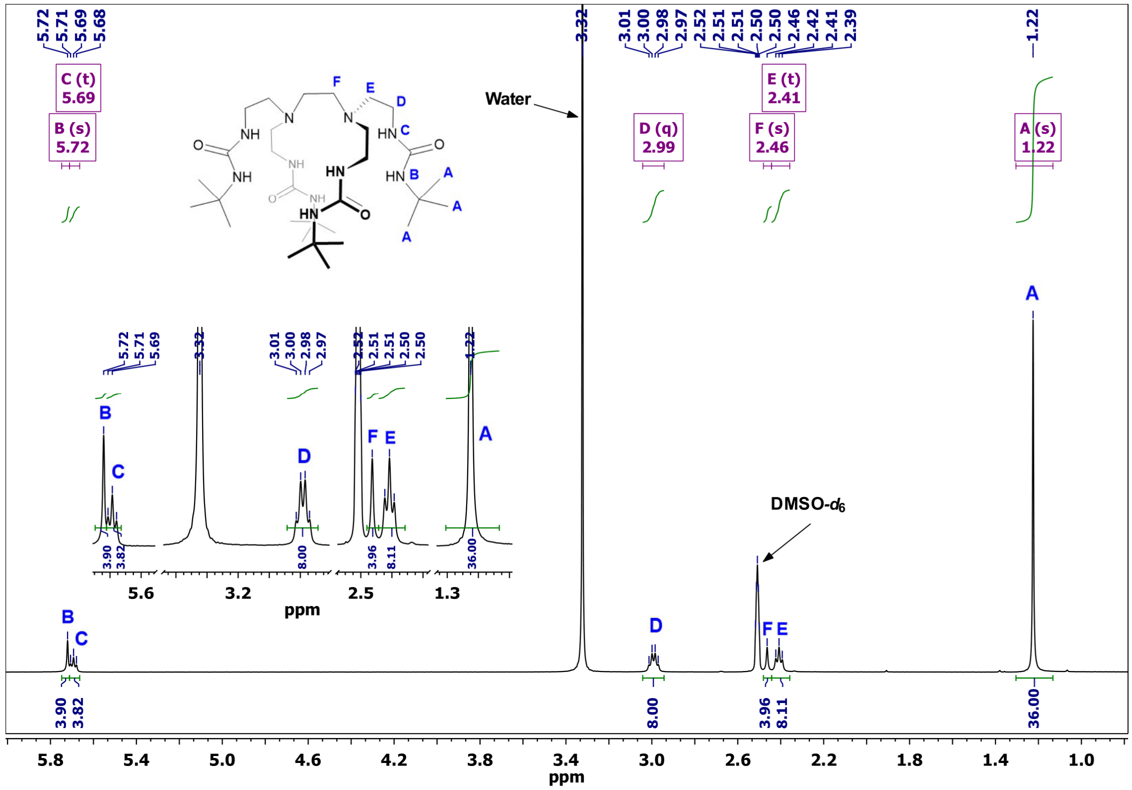

Figure S12: ^{1}H-NMR (400 MHz) spectrum of tetra *tert*-butylurea (**4**) in DMSO-d_6 at 298 K.

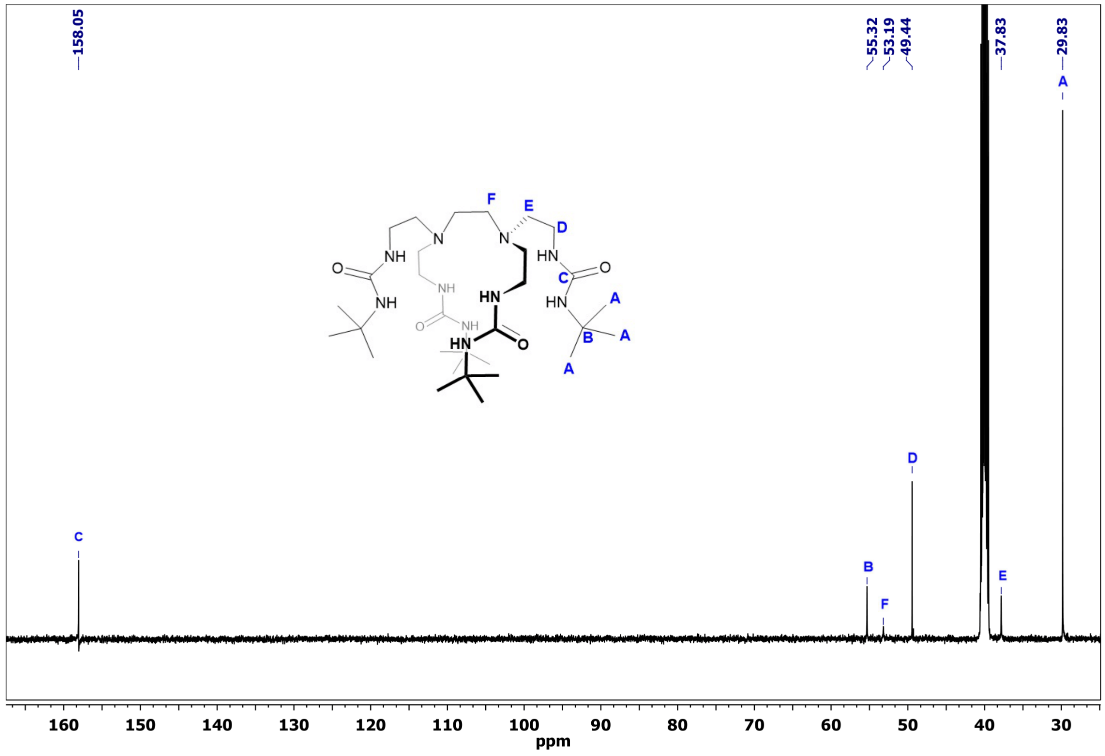

Figure S13: ^{13}C-NMR (126 MHz) spectrum of tetra *tert*-butylurea (**4**) in DMSO-d_6 at 298 K.

S4. Transport Studies:

S4.1 General Experimental Procedures:

Mentioned below describes the procedures shown in the article and is used to analyze the transport properties of the tetrapodal transporters. The synthetic vesicles used through the transport studies were made from 1-palmitoyl-2-oleoyl-sn-glycero-3-phosphocholine (POPC), which was supplied by Avanti Polar Lipids. During both the Cl^-/NO_3^- exchange assay (**S4.2.**) and the cationophore coupled assay (**S4.3.**) chloride concentrations were recorded using an ion-sensitive, in this case, chloride sensitive electrode (ISE) from Accumet. The fluorescence data recorded in the HPTS transport assay (**S4.4.**) was recorded on an Agilent Cary Eclipse Fluorescence Spectrophotometer using the ratiometric probe 8-hydroxypyrene-1,3,6-trisulfonic acid (HPTS).

S4.2 Cl^-/NO_3^- Transport Exchange Assay:

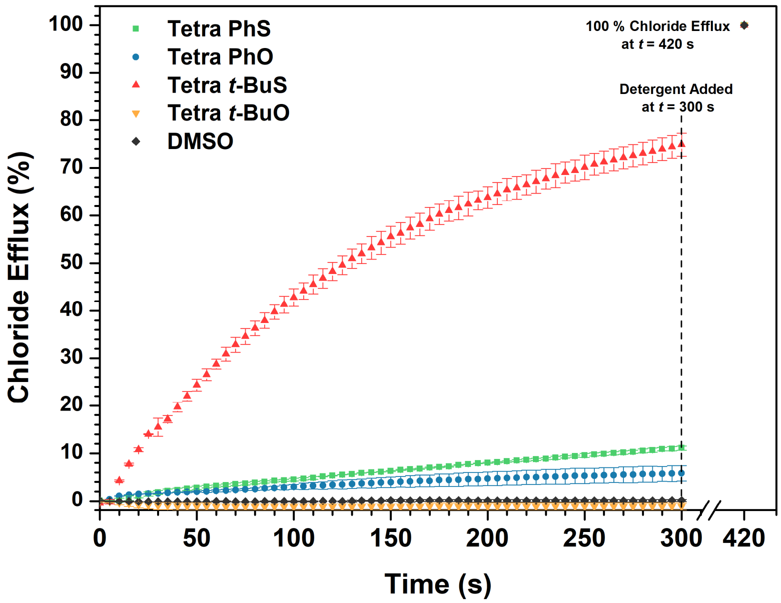

Figure S14: A comparison between the chloride/nitrate transport assay conducted on the four tetrapodal receptors **1 – 4** at 1 mol%.

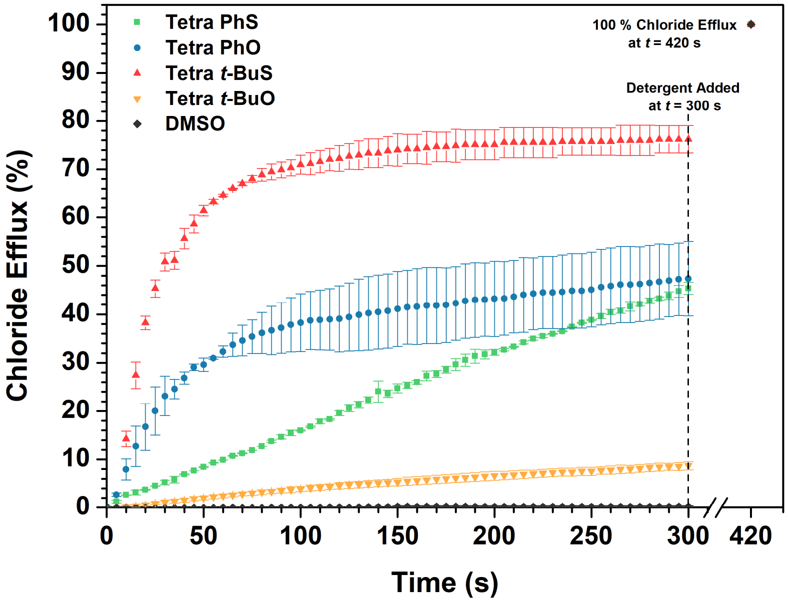

Figure S15: A comparison between the chloride/nitrate transport assay conducted on the four tetrapodal receptors **1 – 4** at 10 mol%.

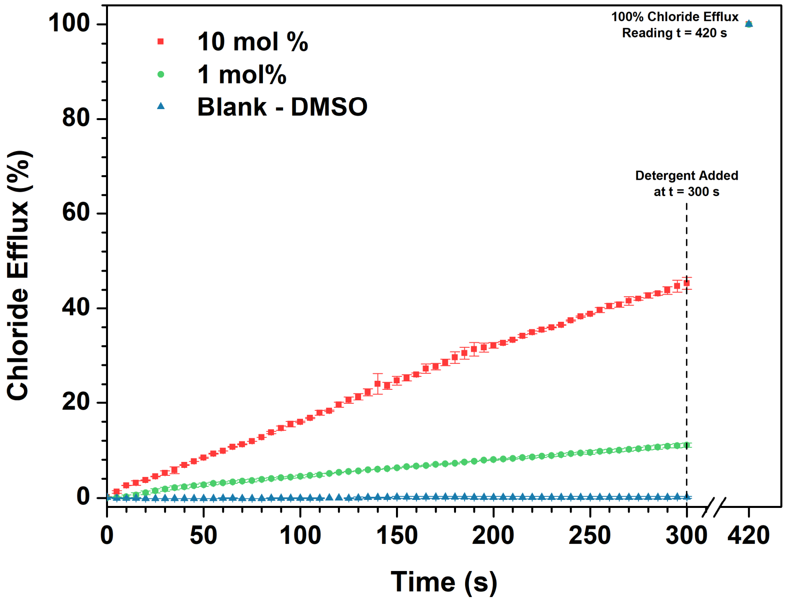

Figure S16: The chloride/nitrate transport assay conducted on tetra phenyl thiourea (**1**).

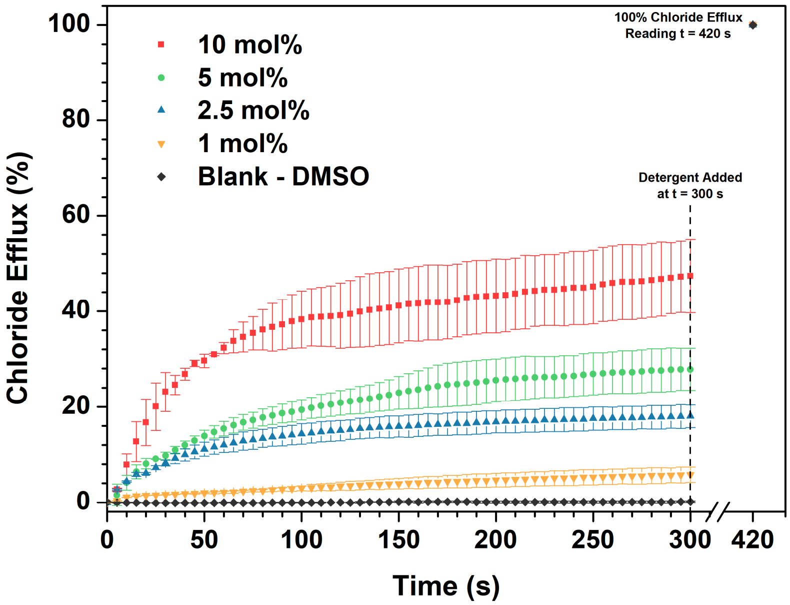

Figure S17: The chloride/nitrate transport assay conducted on tetra phenyl urea (**2**).

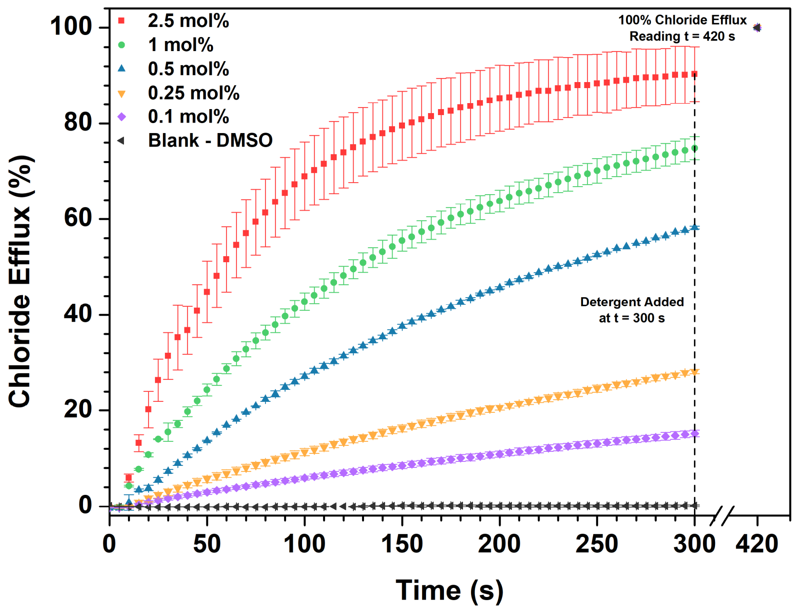

Figure S18: The chloride/nitrate transport assay conducted on tetra *tert*-butyl thiourea (**3**).

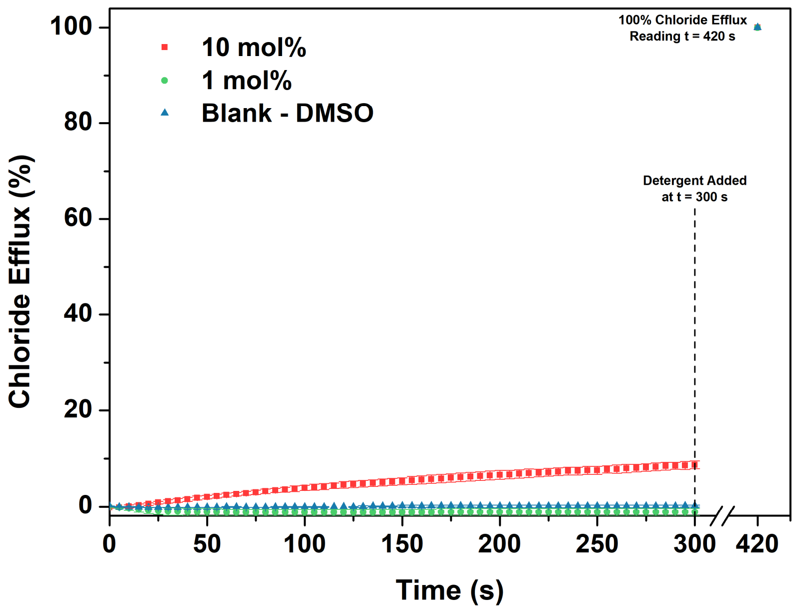

Figure S19: The chloride/nitrate transport assay conducted on tetra *tert*-butyl urea (**4**).

Table S1: Hill coefficients (n) and EC_{50} (mol%) values from the Cl^-/NO_3^- exchange assay.

**Receptor**	**n**[a]	**EC_{50}**[b]
Tetra PhO (**2**)	2	3.92
Tetra *t*-BuS (**3**)	2	0.408

Receptors **1** – **4** were not included due to a lack of activity and unreliable Hill fitting. [a] The Hill coefficient determined from hill analysis of the chloride efflux observed at 270 s. [b] The EC_{50} values (mol%) derived via hill analysis of the chloride efflux at 270 s.

S4.3 Cationophore Coupled Transport Assay:

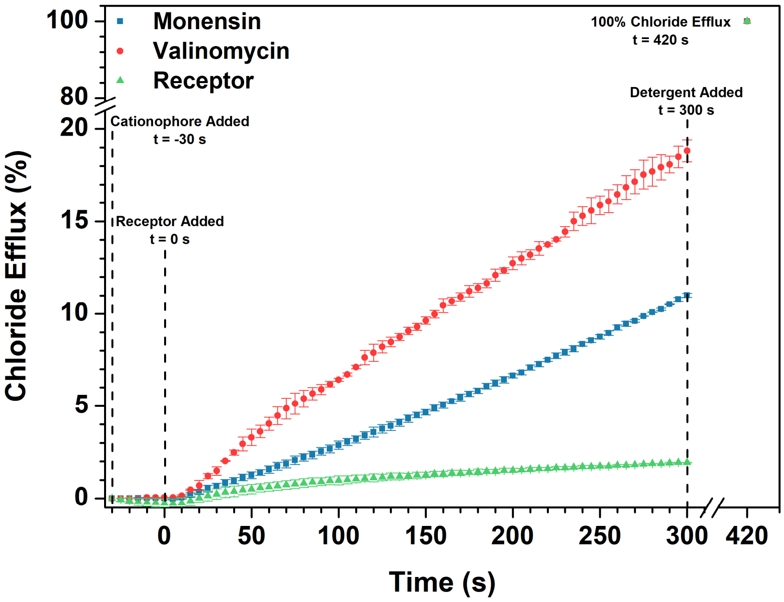

Figure S20: The chloride efflux facilitated by tetra phenyl thiourea (**1**) when no additional cationophore was in solution (green), monensin was added to see if electroneutral transport could occur (blue) and valinomycin was added to show electrogenic transport (red).

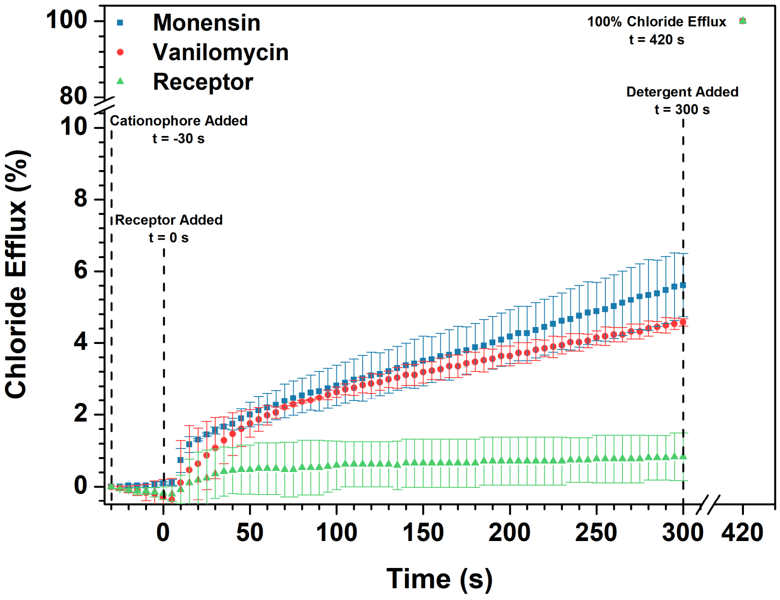

Figure S21: The chloride efflux facilitated by tetra phenyl urea (**2**) when no additional cationophore was in solution (green), monensin was added to see if electroneutral transport could occur (blue) and valinomycin was added to show electrogenic transport (red).

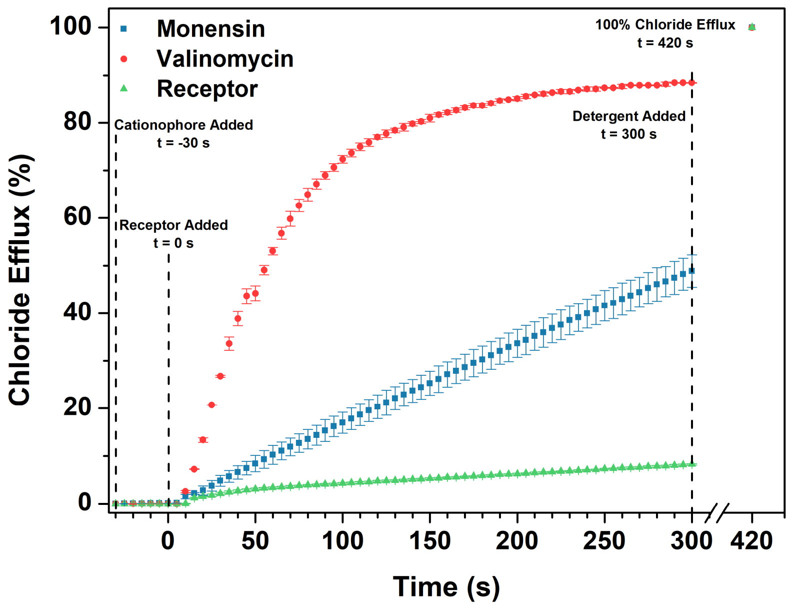

Figure S22: The chloride efflux facilitated by tetra *tert*-butyl thiourea (**3**) when no additional cationophore was in solution (green), monensin was added to see if electroneutral transport could occur (blue) and valinomycin was added to show electrogenic transport (red).

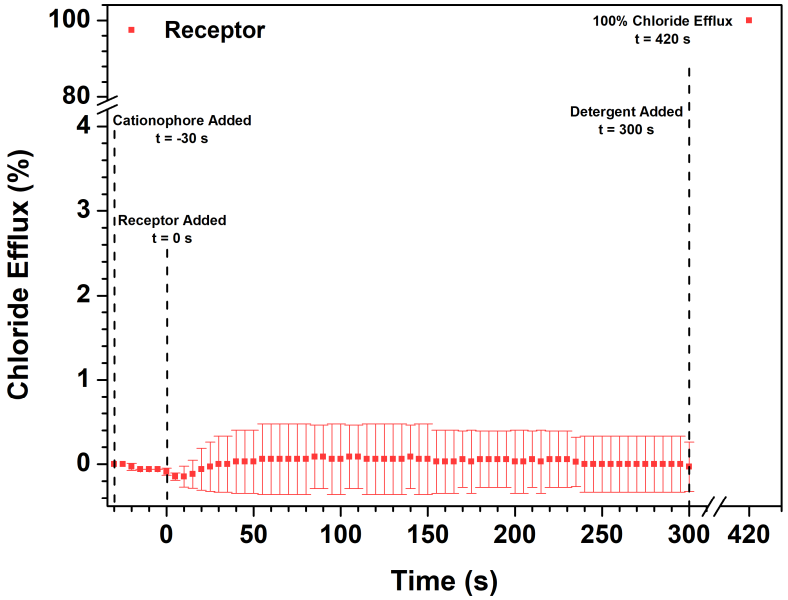

Figure S23: The chloride efflux facilitated by tetra *tert*-butyl urea (**4**, red). The cationophores, monensin and valinomycin, were not analysed in this study as the addition of the receptor caused interference with the electrode and precipitated out of solution at higher concentrations. This resulted in inaccurate data with a high error.

S4.4 HPTS Transport Selectivity Assay:

The HPTS experimental data underwent fitting to find the EC_{50} (mol%, at 200 s) of the receptors being tested as well as the Hill Coefficient (*n*). The receptors were analysed in three different systems; in the presence of fatty acids (FA) where the receptors were screened for chloride/proton cotransport via fatty acid flip flop, anion uniport in vesicles treated with the Gramicidin D (GRA) proton channel and chloride/proton symport without the aid of membrane-embedded fatty acids in vesicles treated with bovine serum albumin (BSA, 0.1 mol%).[3]

Table S2: The comparison of EC_{50}, Hill Coefficients (*n*) and enhancement factors (F) for the HPTS NMDG-Cl assay of both the tetrapodal and tripodal receptors.

Tetrapodal Receptors	EC_{50} (mol%)[a]			Hill Coefficient (*n*)[e]			$F_{(Cl^-/H^+ Symport)}$[i]	$F_{(Cl^- Uniport)}$[j]	$F_{(Selectivity)}$[k]
	FA[b]	GRA[c]	BSA[d]	FA[f]	GRA[g]	BSA[h]			
Tetra PhS (**1**)	0.26	0.32	0.72	1.60 (0.32)	1.28 (0.24)	1.25 (0.73)	2.78	2.26	0.81
Tetra PhO (**2**)	1.94	1.63	2.23	1.31 (0.19)	1.47 (0.24)	1.43 (0.14)	1.15	1.37	1.19
Tetra *t*-BuS (**3**)	0.17	0.019	0.60	0.98 (0.11)	1.1 (0.05)	1.37 (0.13)	3.45	31.48	9.14
Tetra *t*-BuO (**4**)	n.d. [o]	n.d. [o]	n.d. [o]	n.d. [o]	n.d. [o]	n.d. [o]	n.d. [o]	n.d. [o]	n.d. [o]
**Tripodal Receptors**									
Tris PhS	0.0081 [m]	0.0021 [m]	0.12 [m]	1.14 (4.3) [m]	1.58 (1.21) [m]	1.43 (0.45) [m]	14.81 [m]	57.14 [m]	3.86 [m]
Tris PhO	0.78 [m]	0.3 [m]	9.8 [m]	1.86 (0.47) [m]	1.04 (1.32) [m]	1.81 (0.58) [m]	12.53 [m]	32.57 [m]	2.60 [m]
Tris *t*-BuS	0.012 [n]	0.0048 [n]	0.29 [n]	1.49 (0.20) [n]	1.98 (1.08) [n]	1.52 (1.31) [n]	24.38 [n]	61.20 [n]	2.51 [n]

Errors have are denoted inside the brackets beside the derived value. [a] The concentration of the receptor, which promotes 50% of the maximum chloride efflux (EC_{50}, mol%, at 200 s). [b, c and d] The EC_{50} values of; the non-treated vesicles with fatty-acid impurities (FA) in the membrane, the vesicles treated with the proton channel Gramicidin D (0.1 mol%, GRA) and the vesicles treated with bovine serum albumin (BSA). [e] The Hill Coefficient of the tests showing that when $n = 1$ there is independent binding and when $n > 1$ there is cooperative binding. [f, g and h] The calculated Hill Coefficients produced from the Hill Fit.[i, j and k] The enhancement factors of the receptors undergoing Cl^-/H^+ symport, Cl^- uniport and the enhancement factor of chloride transport over Cl^-/H^+ cotransport. A value of $F \approx 1$ means that the mode of transport maintained but no increase was seen over the other mode of transport and when $F > 1$, the mode of transport being compared is enhanced and is the preferential mode of transport in the system. [m] Previously published data.[4] [n] Previously published data.[5] [o] Not determined due to insufficient activity.

The enhancement factors (F) were calculated by dividing the EC_{50} values of the experiments being compared:

$$F_{(Cl^-/H^+\ symport)} = EC_{50(BSA)}/EC_{50(FA)}$$

Equation S1: The chloride/proton symport enhancement factor equation.

$$F_{(Cl^-\ uniport)} = EC_{50(BSA)}/EC_{50(GRA)}$$

Equation S2: The chloride uniport enhancement factor equation.

$$F_{(selectivity)} = EC_{50(FA)}/EC_{50(GRA)}$$

Equation S3: The anion-selective enhancement factor equation.

When the enhancement factor is $F \approx 1$, the mode of transport being compared is just being maintained, and there is no increase in transport over the alternative mode of transport in the model system. While an enhancement factor over 1 ($F > 1$) means there has been an enhancement of the compared transport mode over the other and is the preferential mode of transport.

Table S3: A comparison of the EC_{50} values from the HPTS NMDG-NO_3 and NMDG-SO_4 assay for the tetrapodal and tripodal receptors.

Tetrapodal Receptors	NMDG-NO_3 EC_{50} (mol%) [a]	NMDG-SO_4 EC_{50} (mol%) [b]
Tetra PhS (**1**)	0.11	n.d. [c]
Tetra PhO (**2**)	0.77	n.d. [c]
Tetra *t*-BuS (**3**)	0.033	n.d. [c]
Tetra *t*-BuO (**4**)	8.5	n.d. [c]
**Tripodal Receptors**		
Tris PhS	0.039 [d]	n.d. [c, d]
Tris PhO	0.4 [d]	n.d. [c, d]

[a] The NMDG-NO_3 assay was conducted in the presence of the proton channel Gramicidin D (0.1 mol%). [b] The NMDG-SO_4 assay was conducted in the presence of vesicles with fatty acid impurities. [c] The value could not be determined. [d] The result was determined in a previous study.[4]

The NMDG-NO_3 assay was only conducted in vesicles treated with Gramicidin D rather than BSA and FA treated vesicles as only the maximum NO_3^- transport rate (lowest EC_{50}) was needed to be compared with the maximum transport of the same receptors in the NMDG-Cl assay. The NMDG-SO_4 assay was only conducted on vesicles that still had the fatty acids in the membrane to observe any possible transport as well as possible interference.

Table S4: A comparison between the transport selectivity of Cl^- transport, Cl^- vs H^+ transport and Cl^- vs NO_3^- transport promoted by the change in receptor design.

Receptor	Cl^- vs H^+ Transport Selectivity ($F_{(selectivity)}$) for each Receptor [a]	Change in Cl^- vs H^+ Transport due to Design [b]	Change in Cl^- vs NO_3^- Transport due to Design [c]	Change in Cl^- vs transport due to Design [d]
Tetra PhS (**1**)	0.81	0.21	0.02	0.01
Tris PhS	3.86 [e]			
Tetra PhO (**2**)	1.19	0.46	0.36	0.18
Tris PhO	2.60 [e]			
Tetra *t*-BuS (**3**)	9.14	3.64	n.d. [f]	0.25
Tris *t*-BuS	2.51 [e]			

[a] The enhancement factor ($F_{(selectivity)}$) of each receptor for chloride transport over chloride/proton cotransport. [b] The change in Cl^- vs H^+ transport is calculated by (Tetra $F_{(selectivity)}$ / Tris $F_{(selectivity)}$). [c] The change in Cl^- vs NO_3^- transport is calculated by ((Tetra $1/EC_{50}$ of Cl^- ÷ Tris $1/EC_{50}$ of Cl^-) ÷ (Tetra $1/EC_{50}$ of NO_3^- ÷ Tris $1/EC_{50}$ of NO_3^-)). [d] The change in Cl^- transport is calculated by (Tetra $1/EC_{50}$ of Cl^- ÷ Tris $1/EC_{50}$ of Cl^-). [e] The $F_{(selectivity)}$ values of the tripodal receptors were found in a previous study.[5,6]

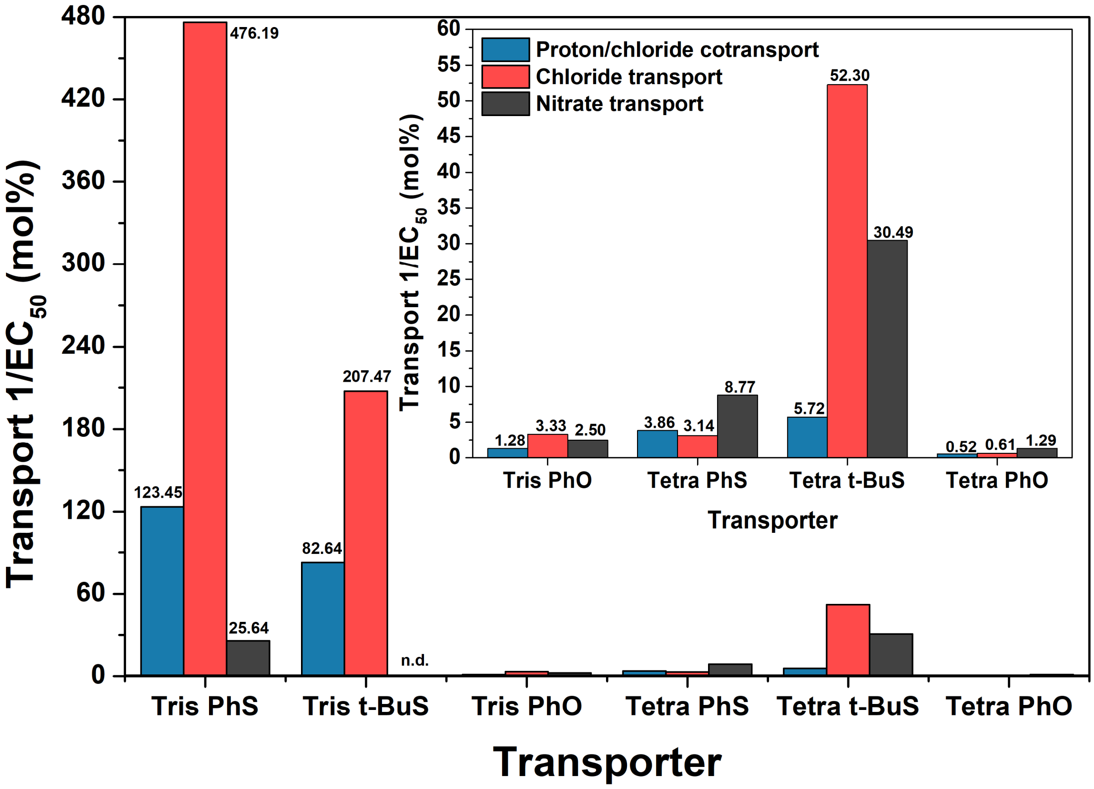

Figure S24: A comparison between the calculated $1/EC_{50}$ values for proton/chloride cotransport, chloride transport and nitrate transport from the different receptors, tetra PhS (**1**), tetra PhO (**2**) and tetra *t*-BuS (**3**).

*Tetra PhS (**1**) NMDG-Cl Transport Studies:*

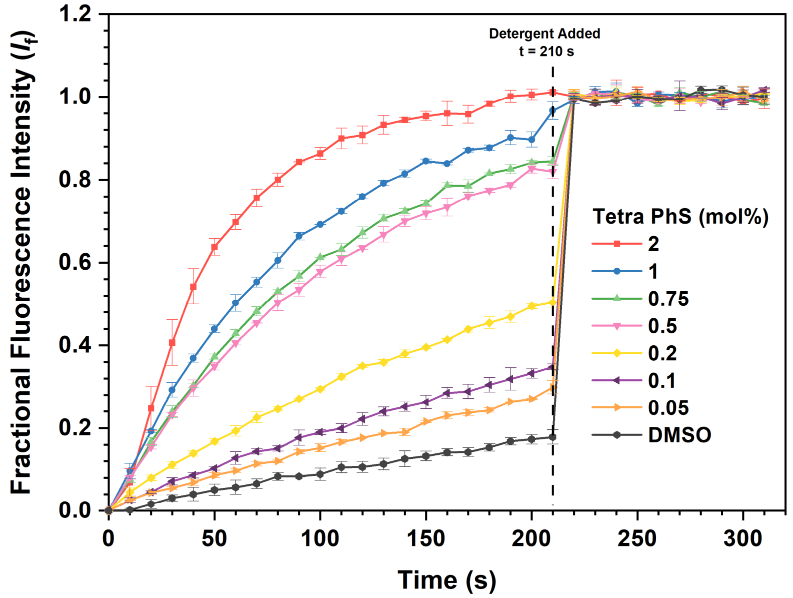

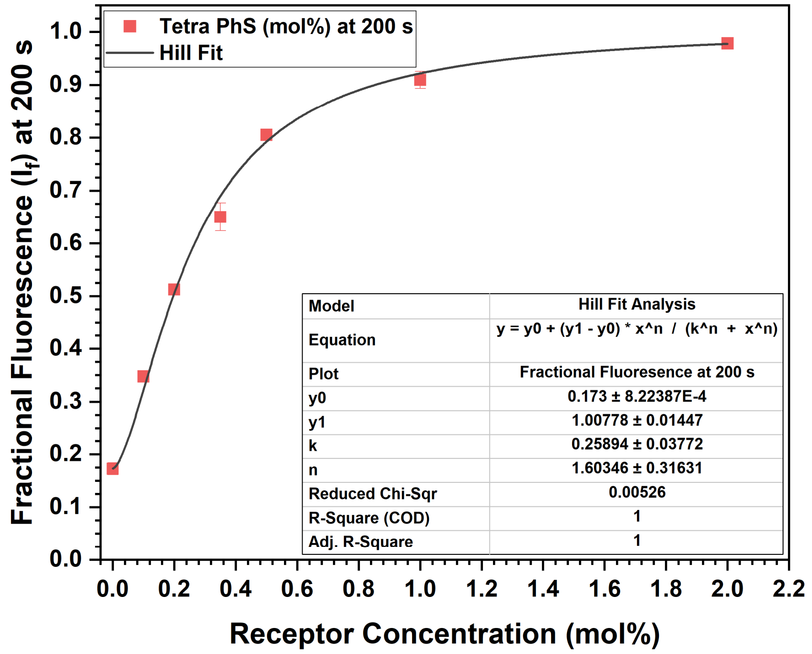

Figure S25: The HPTS NMDG-Cl transport assay of tetra PhS (**1**) conducted on vesicles with fatty acids embedded in the membrane (top) and Hill analysis (bottom).

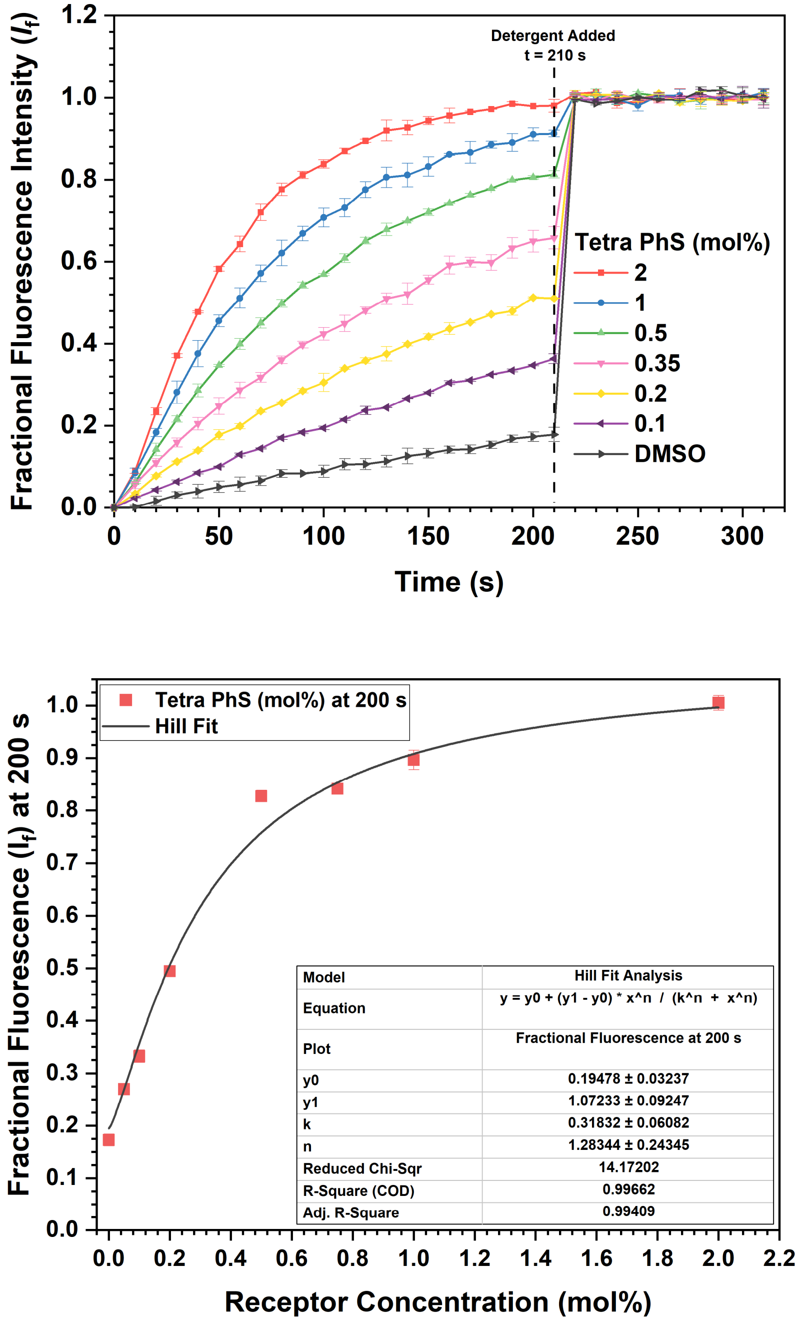

Figure S26: The HPTS NMDG-Cl transport assay of tetra PhS (**1**) conducted on vesicles treated with GRA (top) and Hill analysis (bottom).

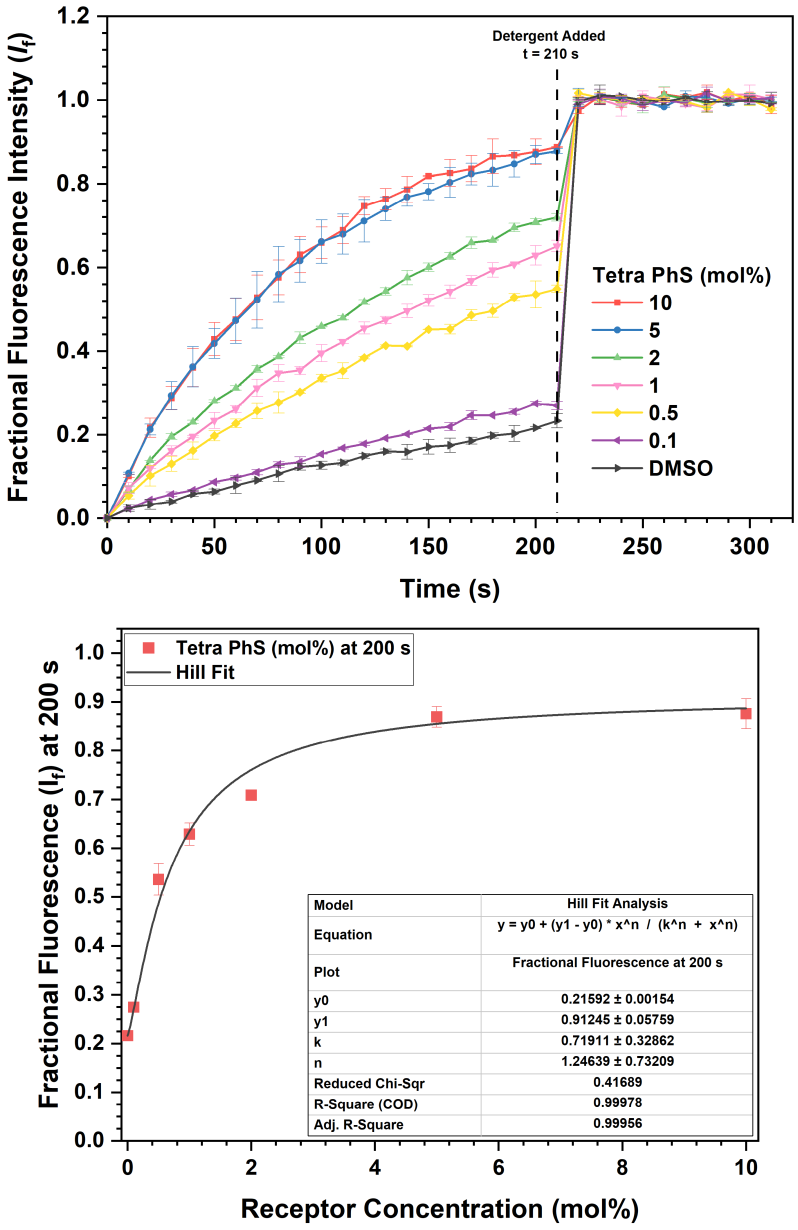

Figure S27: The HPTS NMDG-Cl transport assay of tetra PhS (**1**) conducted on vesicles treated with BSA (top) and Hill analysis (bottom).

*Tetra PhO (**2**) NMDG-Cl Transport Studies*

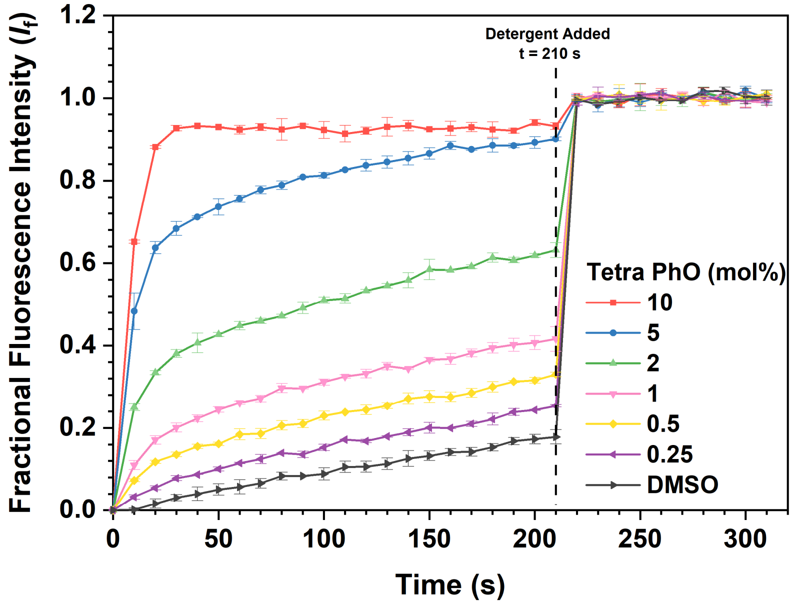

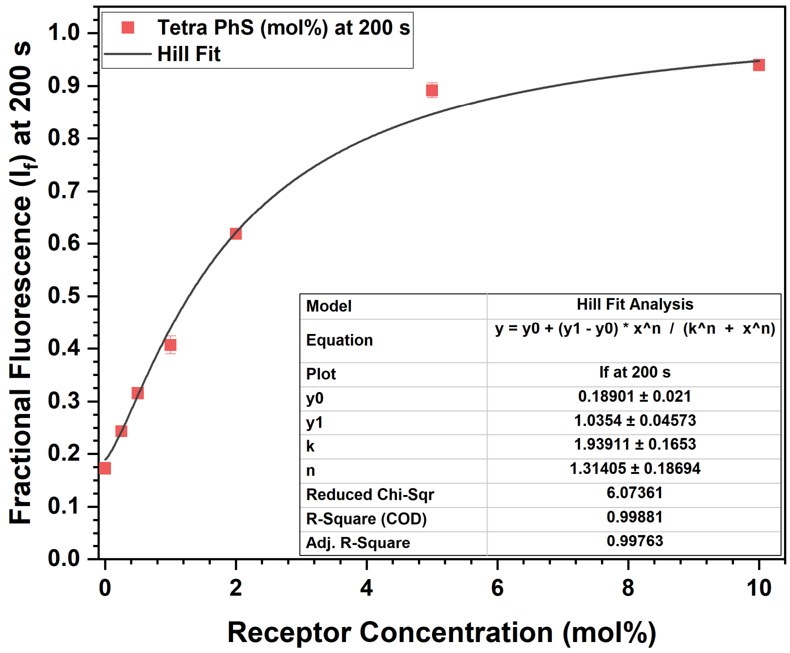

Figure S28: The HPTS NMDG-Cl transport assay of tetra PhO (**2**) conducted on vesicles with fatty acids embedded in the membrane (top) and Hill analysis (bottom).

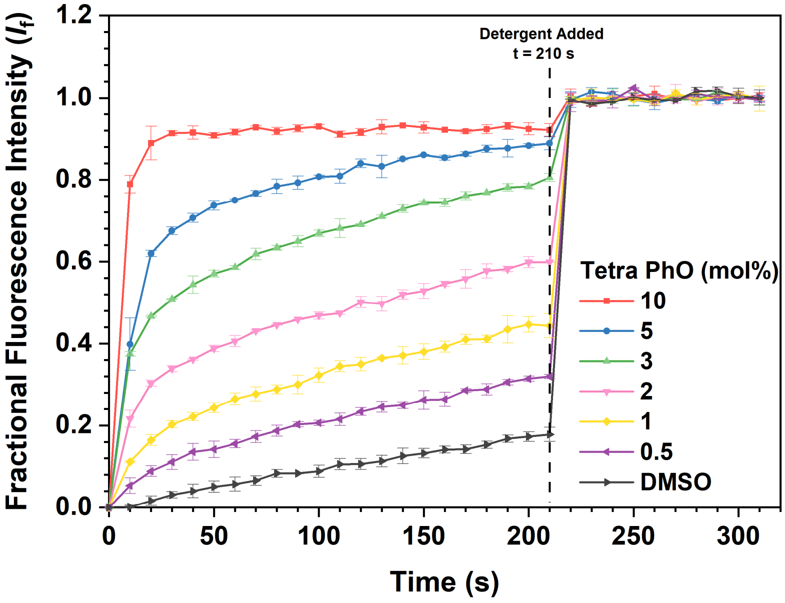

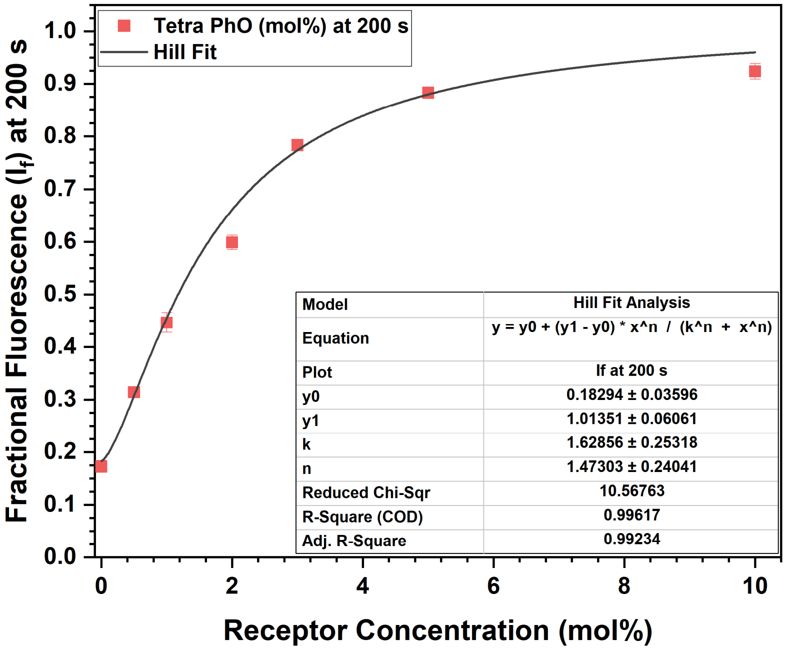

Figure S29: The HPTS NMDG-Cl transport assay of tetra PhO (**2**) conducted on vesicles treated with GRA (top) and Hill analysis (bottom).

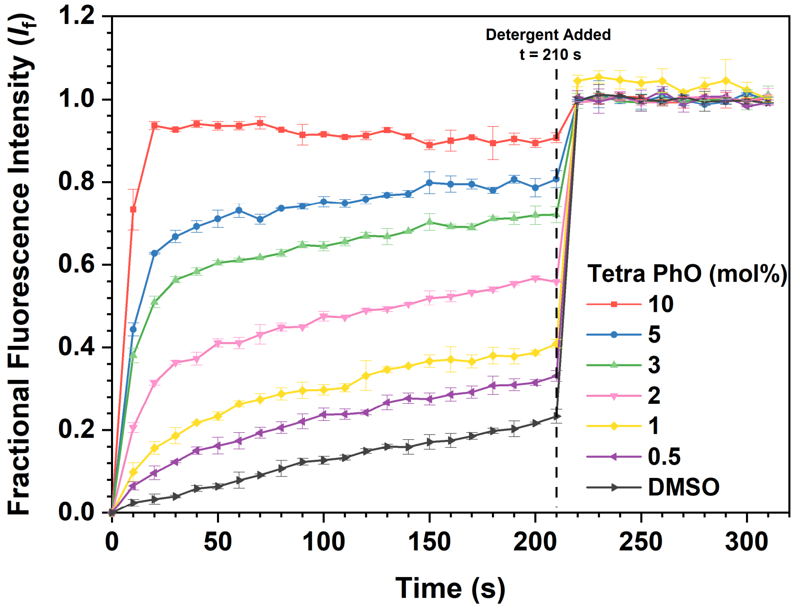

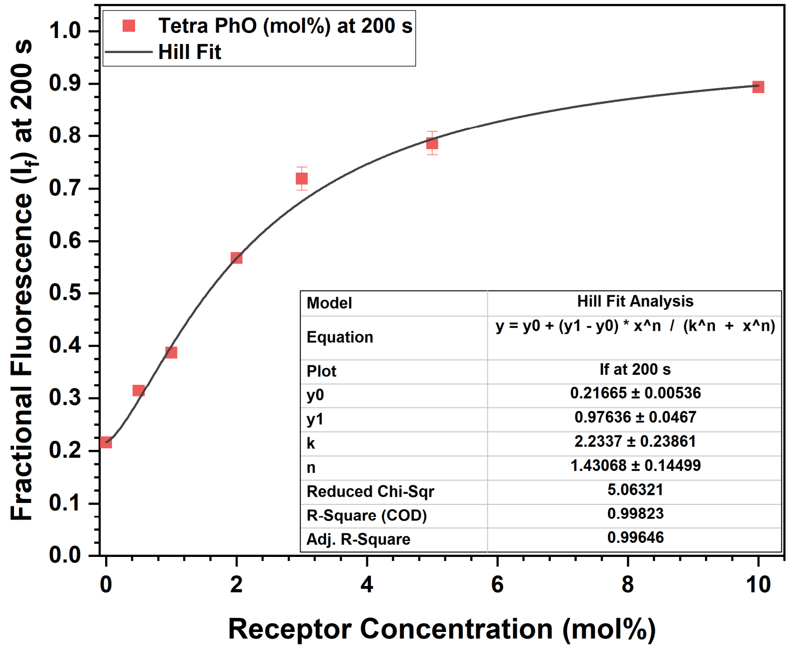

Figure S30: The HPTS NMDG-Cl transport assay of tetra PhO (**2**) conducted on vesicles treated with BSA (top) and Hill analysis (bottom).

*Tetra t-BuS (**3**) NMDG-Cl Transport Studies:*

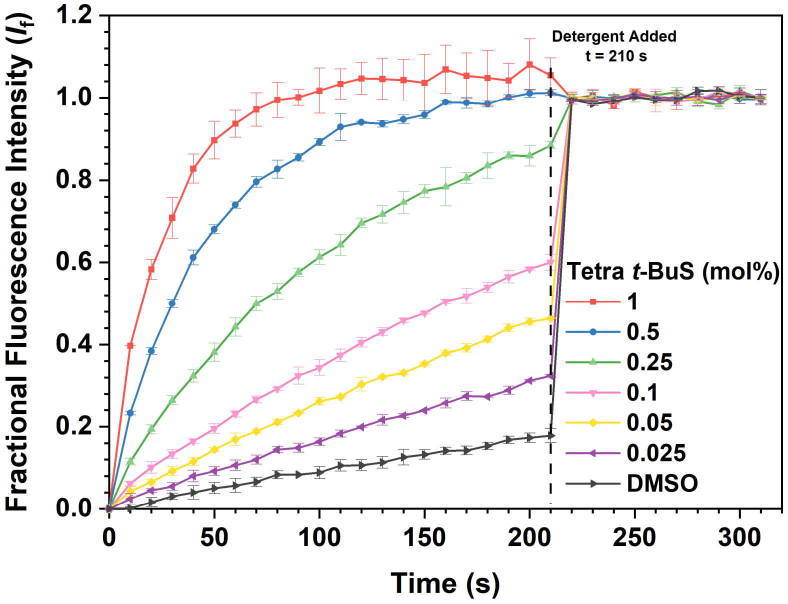

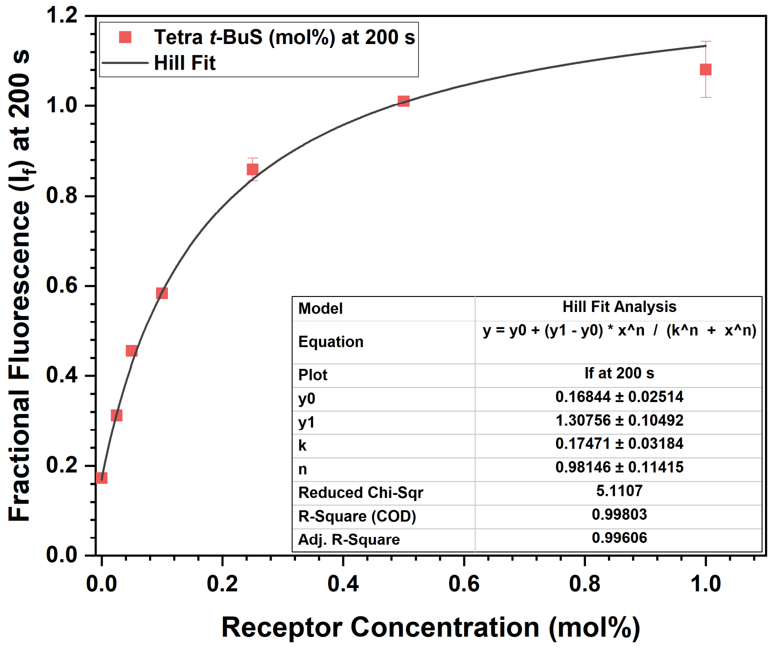

Figure S31: The HPTS NMDG-Cl transport assay of tetra *t*-BuS (**3**) conducted on vesicles with fatty acids embedded in the membrane (top) and Hill analysis (bottom).

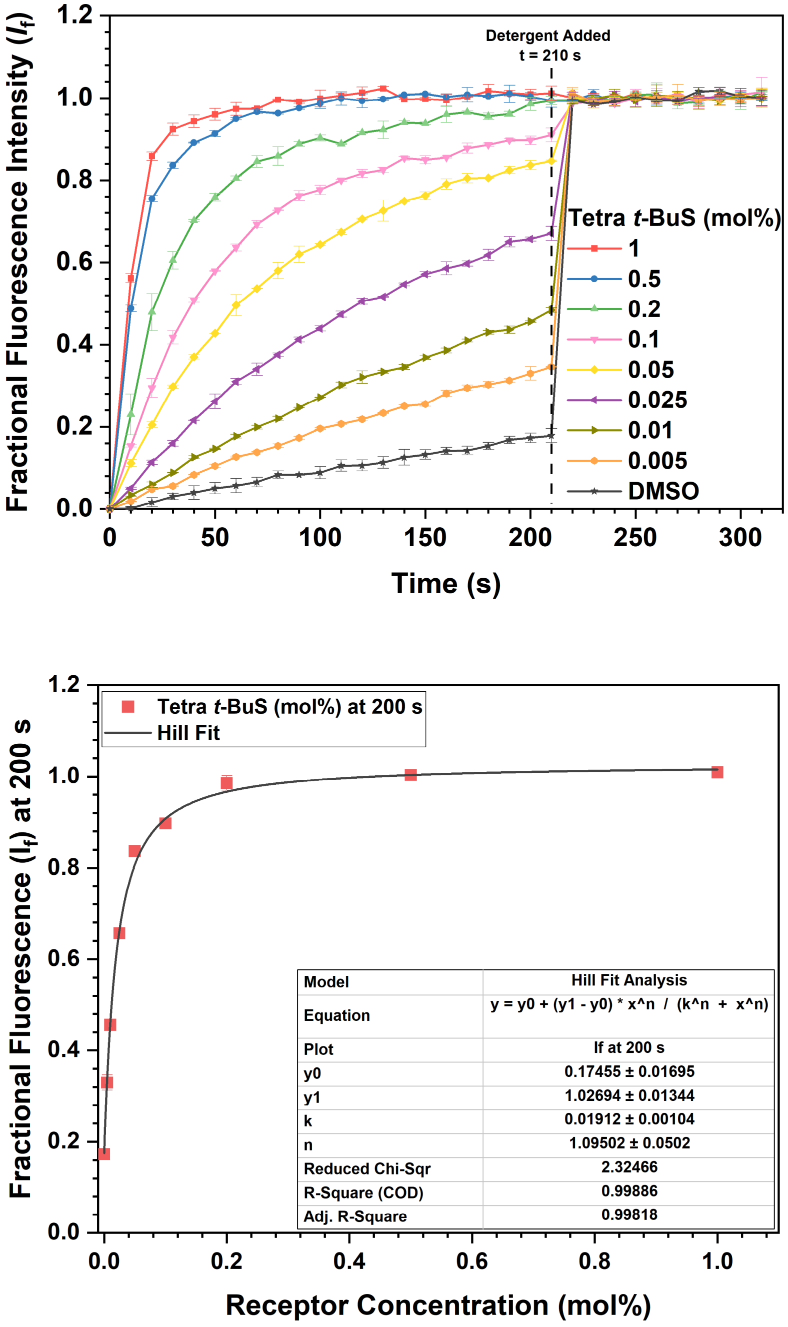

Figure S32: The HPTS NMDG-Cl transport assay of tetra *t*-BuS (**3**) conducted on vesicles treated with GRA (top) and Hill analysis (bottom).

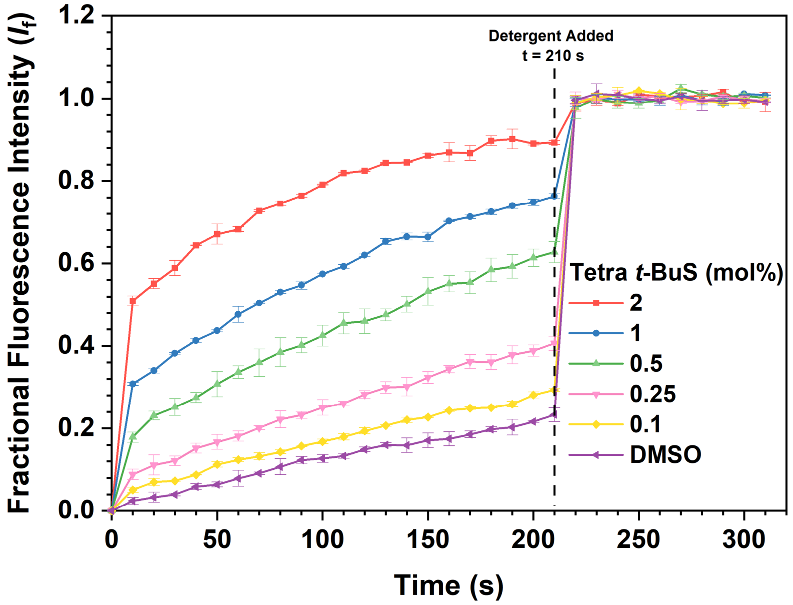

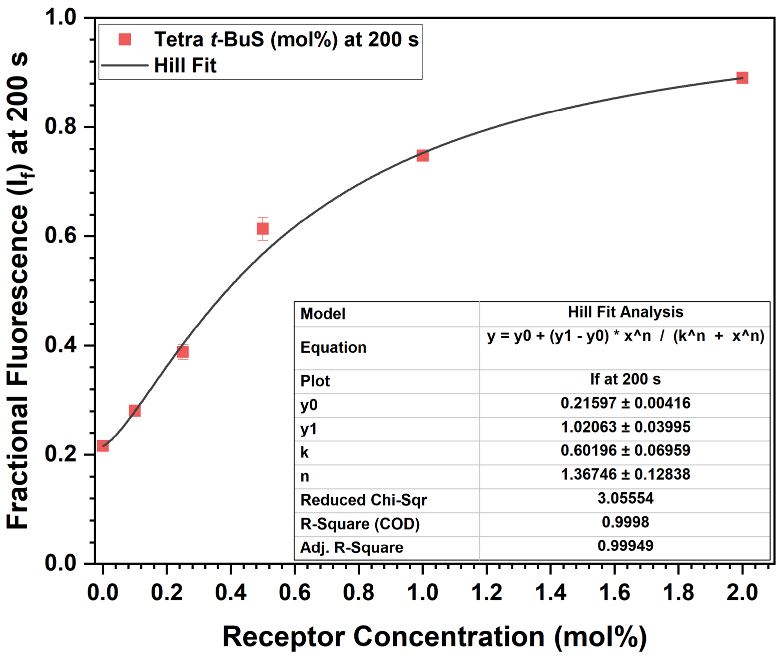

Model	Hill Fit Analysis
Equation	y = y0 + (y1 - y0) * x^n / (k^n + x^n)
Plot	If at 200 s
y0	0.21597 ± 0.00416
y1	1.02063 ± 0.03995
k	0.60196 ± 0.06959
n	1.36746 ± 0.12838
Reduced Chi-Sqr	3.05554
R-Square (COD)	0.9998
Adj. R-Square	0.99949

Figure S33: The HPTS NMDG-Cl transport assay of tetra *t*-BuS (**3**) conducted on vesicles treated with BSA (top) and Hill analysis (bottom).

Tetra t-BuO (4) NMDG-Cl Transport Studies:

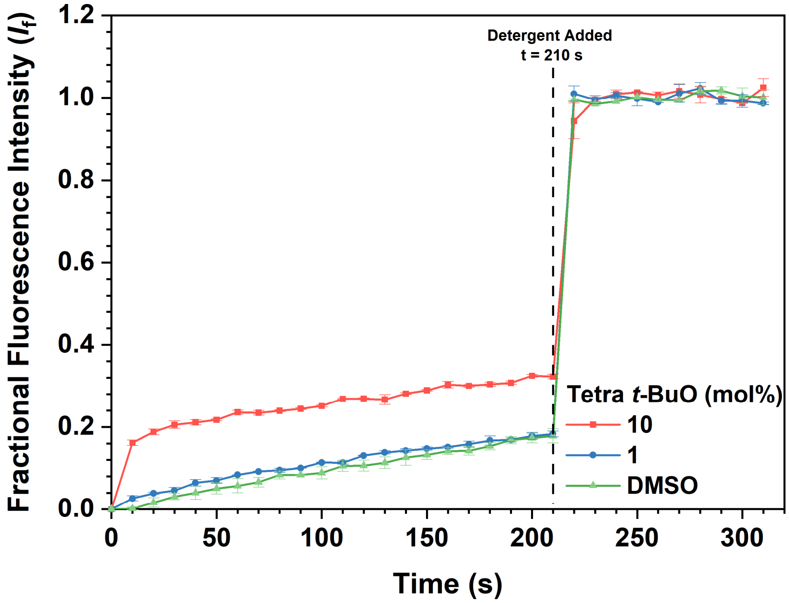

Figure S34: The HPTS NMDG-Cl transport assay of tetra *t*-BuO (**4**) conducted on vesicles with fatty acids embedded in the membrane. Due to the inactivity of this compound, no Hill analysis could be conducted.

*Tetra PhS (**1**) NMDG-NO_3 Transport Studies:*

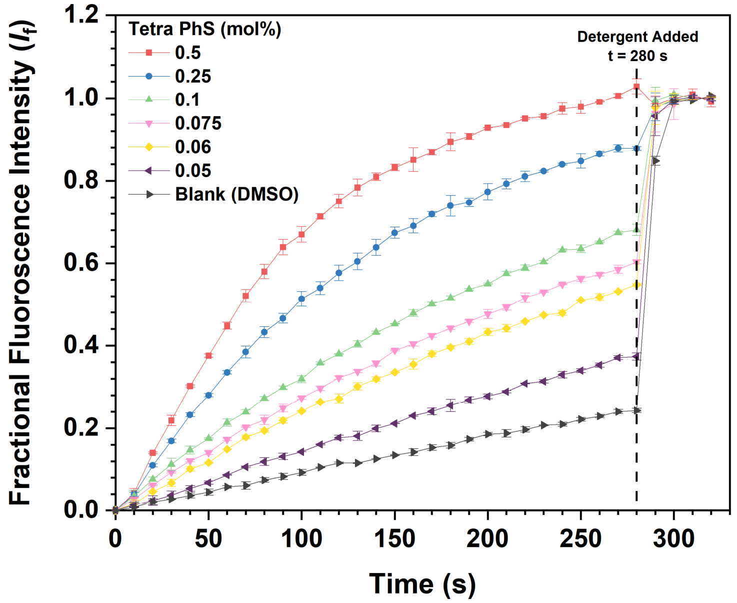

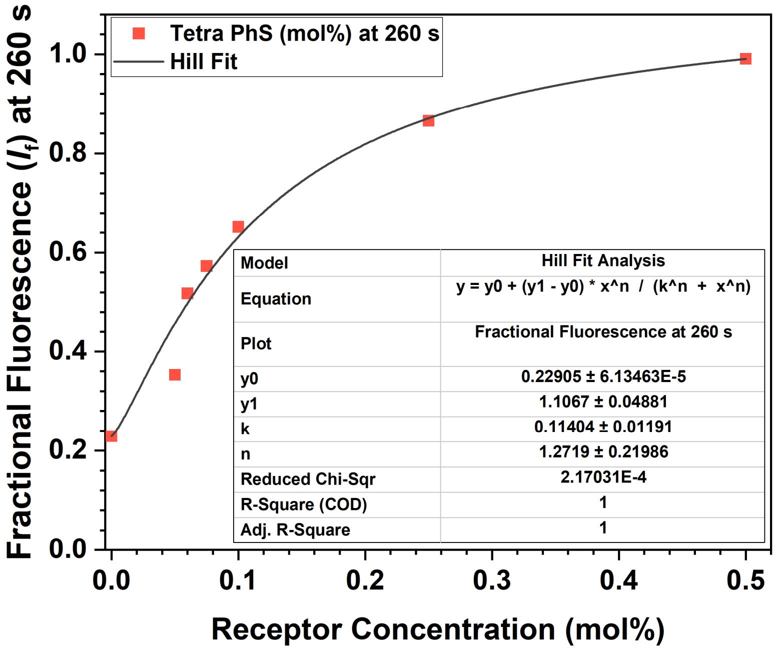

Figure S35: The HPTS NMDG-NO_3 transport assay of tetra PhS (**1**) conducted on vesicles treated with GRA (top) and Hill analysis (bottom).

*Tetra PhO (**2**) NMDG-NO_3 Transport Studies:*

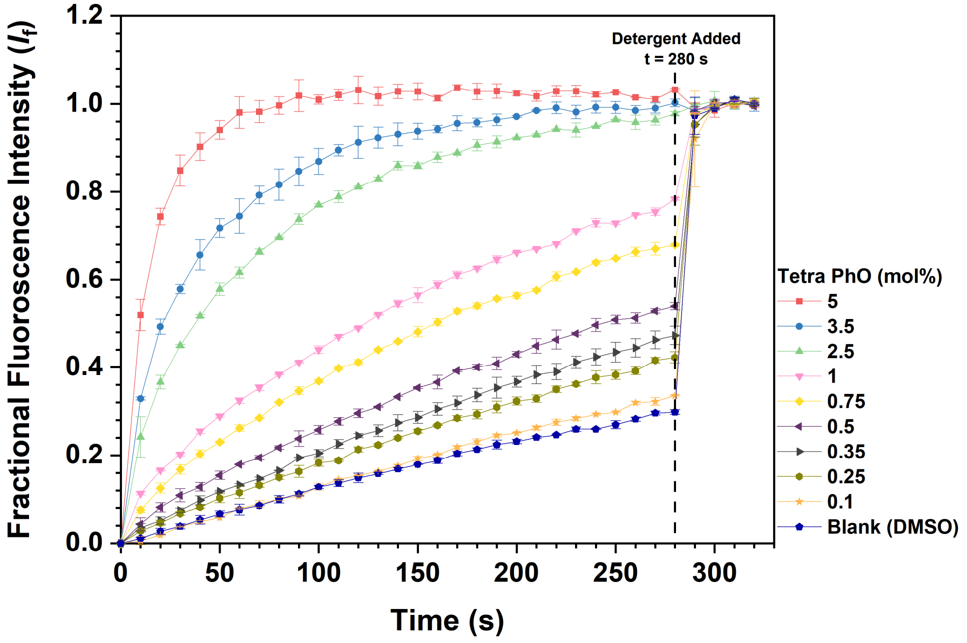

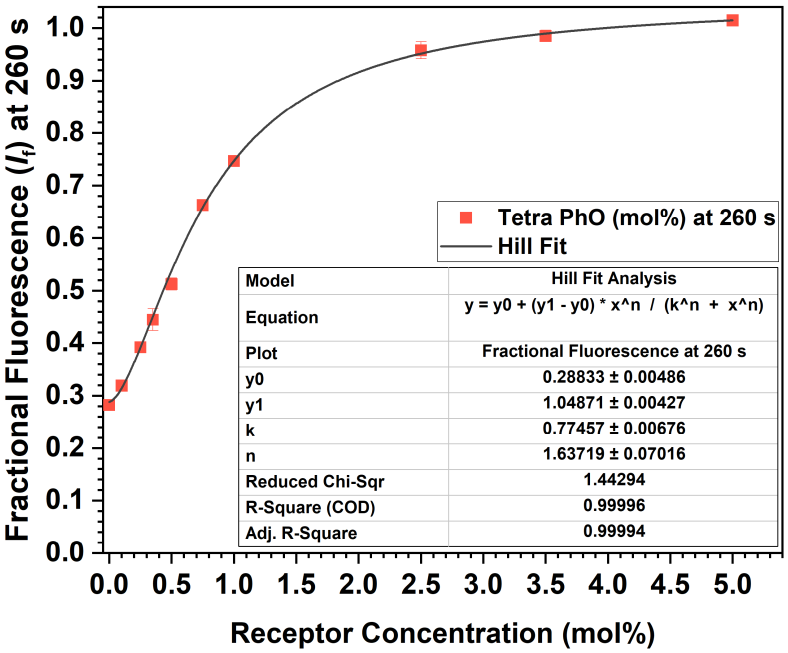

Figure S36: The HPTS NMDG-NO_3 transport assay of tetra PhO (**2**) conducted on vesicles treated with GRA (top) and Hill analysis (bottom).

*Tetra t-BuS (**3**) NMDG-NO_3 Transport Studies:*

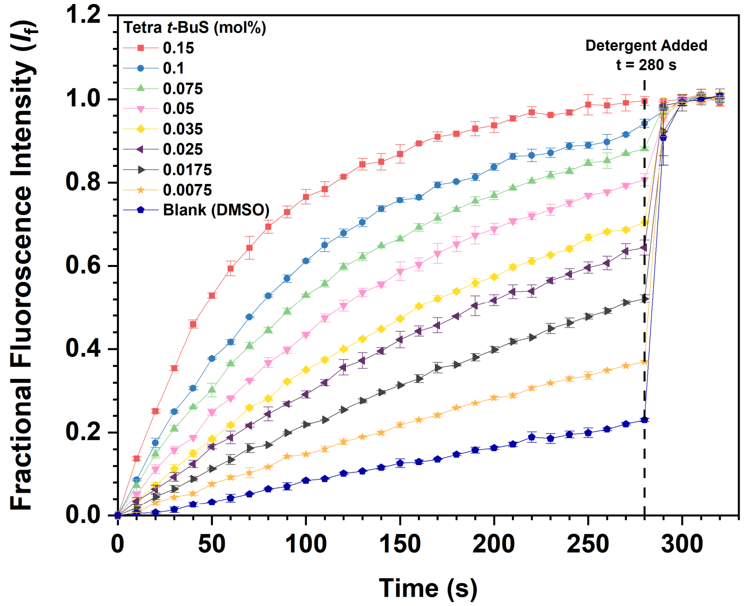

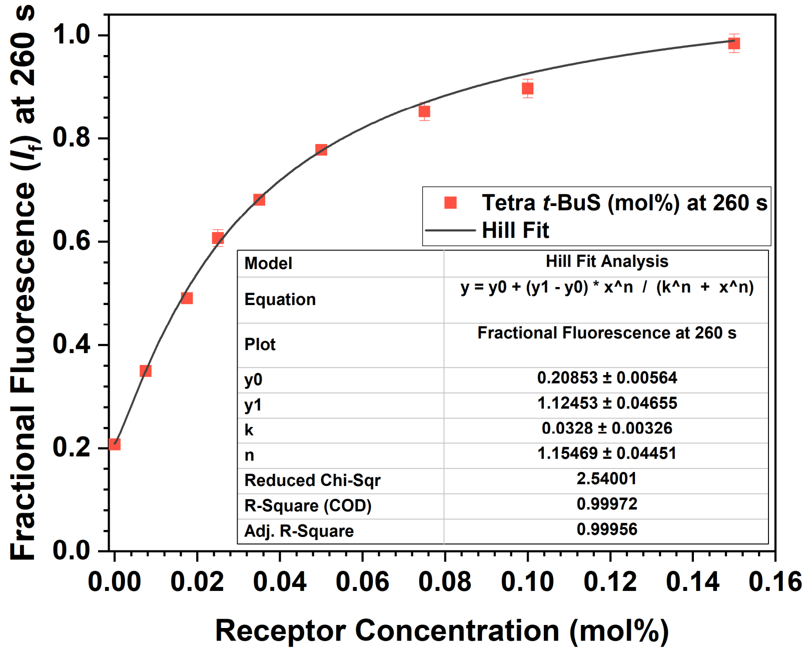

Figure S37: The HPTS NMDG-NO_3 transport assay of tetra *t*-BuS (**3**) conducted on vesicles treated with GRA (top) and Hill analysis (bottom).

Tetra t-BuO (4) NMDG-NO_3 Transport Studies:

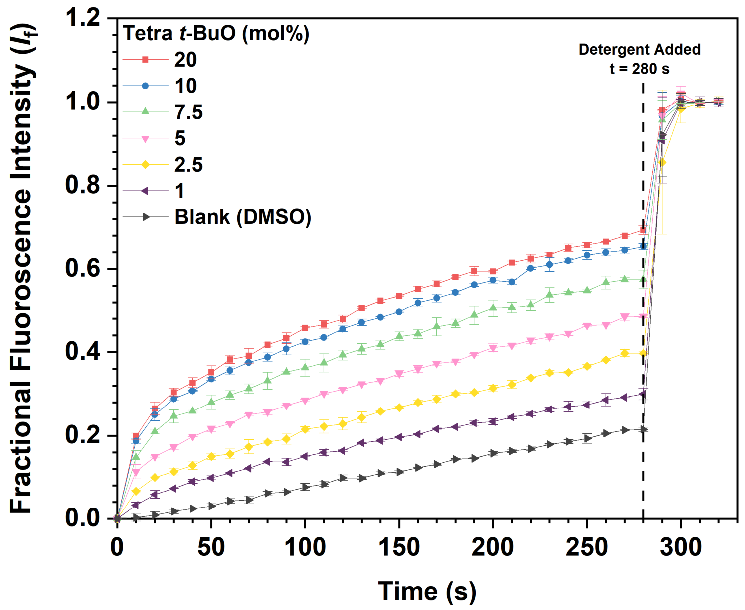

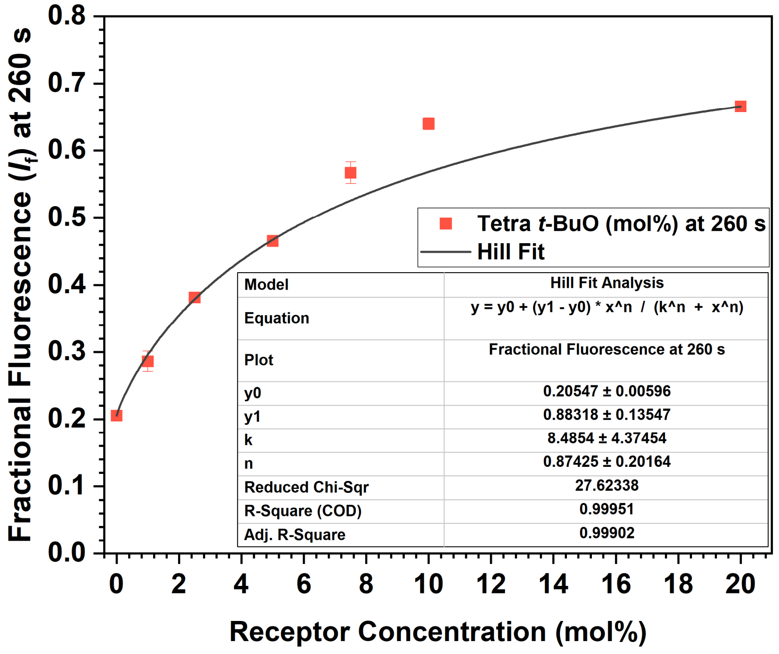

Figure S38: The HPTS NMDG-NO_3 transport assay of tetra *t*-BuO (**4**) conducted on vesicles treated with GRA (top) and Hill analysis (bottom).

*Tetra PhS (**1**) and tetra PhO (**2**) NMDG-SO_4 Transport Studies:*

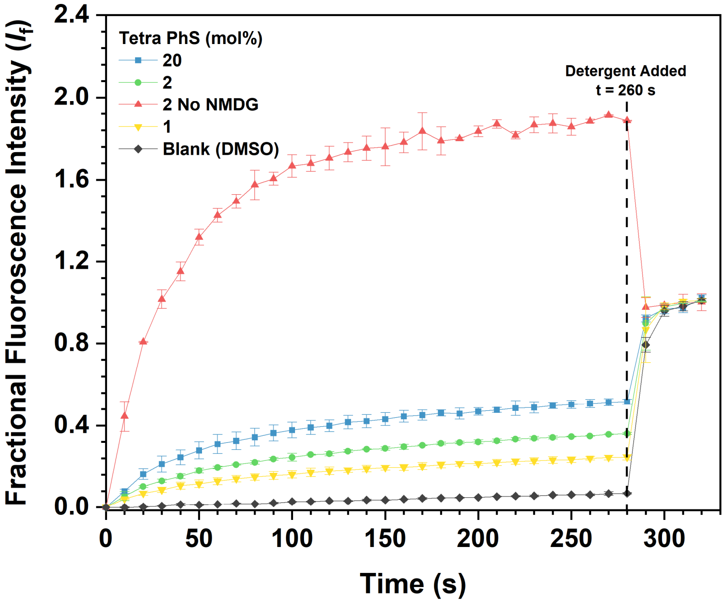

Figure S39: The HPTS NMDG-SO_4 transport assay of tetra PhS (**1**) conducted on vesicles with fatty acids embedded in the membrane. A false positive was conducted where no NMDG base pulse was added at the beginning to initiate transport to see if interference was occurring.

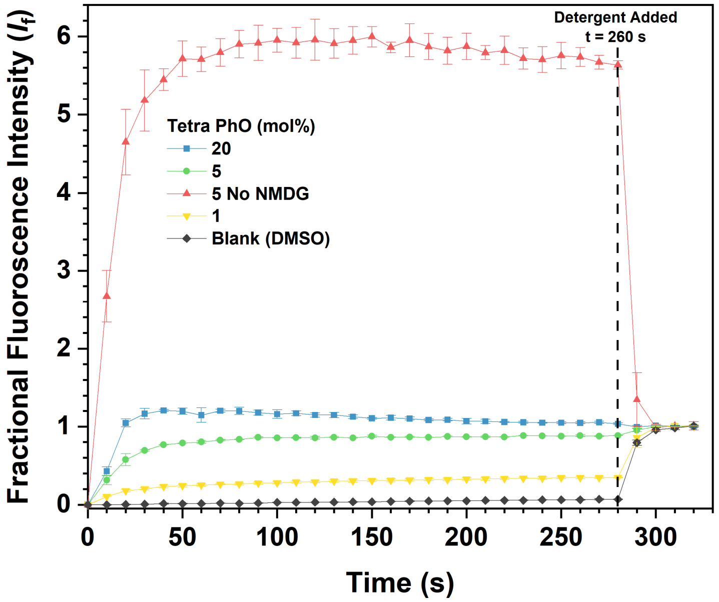

Figure S40: The HPTS NMDG-SO_4 transport assay of tetra PhO (**2**) conducted on vesicles with fatty acids embedded in the membrane. A false positive was conducted where no NMDG base pulse was added at the beginning to initiate transport to see if interference was occurring.

*Tetra t-BuS (**3**) and tetra t-BuO (**4**) NMDG-SO_4 Transport Studies:*

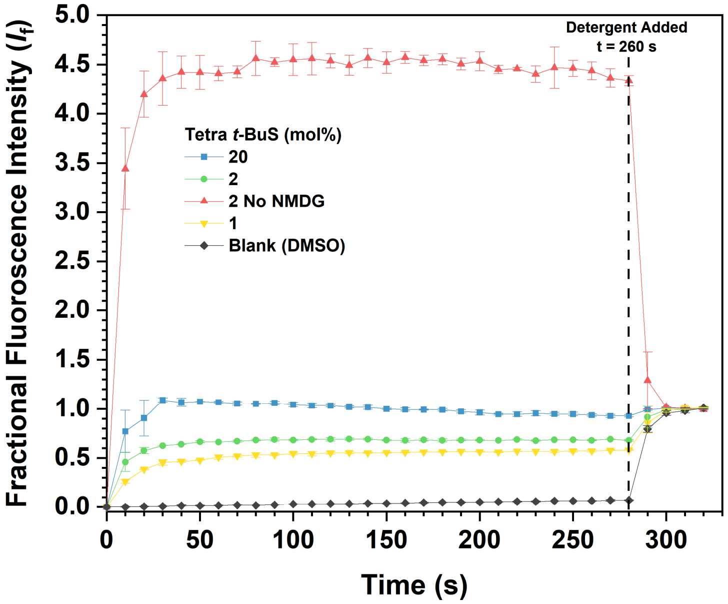

Figure S41: The HPTS NMDG-SO_4 transport assay of tetra *t*-BuS (**3**) conducted on vesicles with fatty acids embedded in the membrane. A false positive was conducted where no NMDG base pulse was added at the beginning to initiate transport to see if interference was occurring.

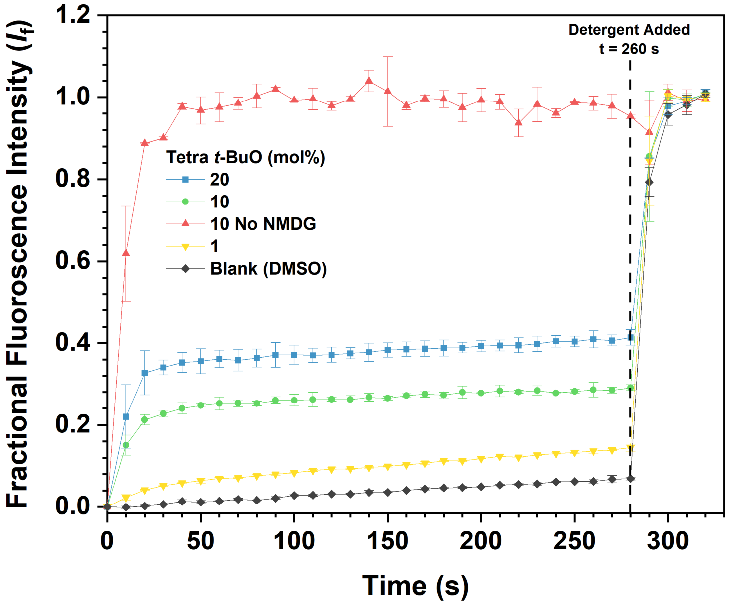

Figure S42: The HPTS NMDG-SO_4 transport assay of tetra *t*-BuO (**4**) conducted on vesicles with fatty acids embedded in the membrane. A false positive was conducted where no NMDG base pulse was added at the beginning to initiate transport to see if interference was occurring.

S4.5 Structure-Activity Relationship:

Initial Rate of Cl^-/NO_3^- Transport:

Table S5: The initial chloride efflux rates ($k_{initial}$, s^{-1}) measured when 1 mol% of the transporter was added in the chloride/nitrate exchange assay.

**Transporter**	**$k_{initial}$ (s^{-1}) of transport at 1 mol%** [a]
Tetra PhS (**1**)	0.074
Tetra PhO (**2**)	0.020
Tetra *t*-BuS (**3**)	0.703
Tetra *t*-BuO (**4**)	0.0
DMSO	0.001

[a] The initial rates of chloride transport ($k_{initial}$) were calculated by fitting the chloride efflux (%) versus time (s) to a non-linear curve with an exponential decay function or fitting via linear-regression analysis.

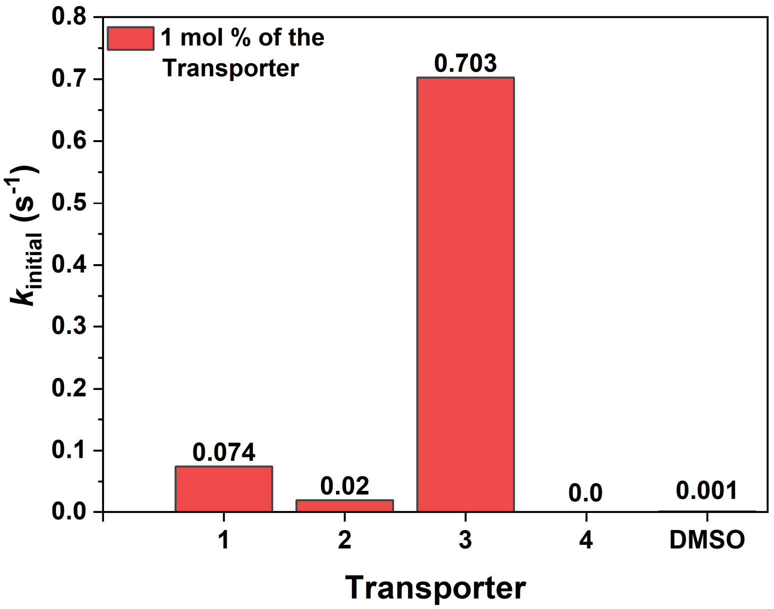

Figure S43: A comparison between the initial transport rates of chloride when 1 mol% of receptors **1 – 4** are added during the chloride/nitrate exchange assay.

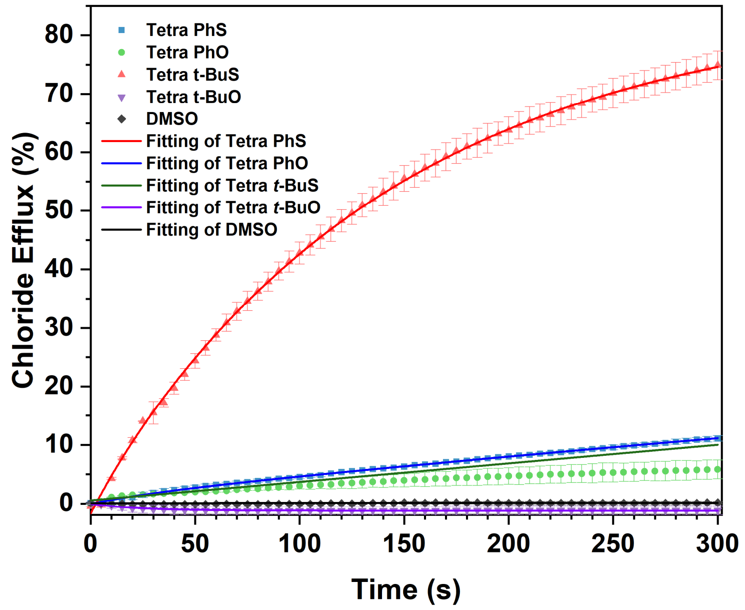

Model	K(initial) Fitting of Tetra PhS
Equation	y = A1*exp(-x/t1) + A2*exp(-x/t2) + y0
Plot	Chloride Efflux
y0	59.1231 ± 32.25216
A1	-1.16832 ± 0.26274
t1	31.01458 ± 10.1796
A2	-58.0138 ± 31.98852
t2	1580.55844 ± 994.03112
Reduced Chi-Sqr	0.23751
R-Square (COD)	0.99997
Adj. R-Square	0.99997

Model	K(initial) Fitting of Tetra t-BuS
Equation	y = A1*exp(-x/t1) + A2*exp(-x/t2) + y0
Plot	Chloride Efflux
y0	0.14274 ± --
A1	-0.03646 ± --
t1	82.11472 ± --
A2	-0.03646 ± --
t2	100.36244 ± --
Reduced Chi-Sqr	--
R-Square (COD)	0
Adj. R-Square	-0.22222

Model	K(initial) Fitting of Tetra PhO
Equation	y = A1*exp(-x/t1) + A2*exp(-x/t2) + y0
Plot	Chloride Efflux
y0	16.83189 ± 15.41799
A1	-7.30834 ± 9565.70982
t1	788.48259 ± 612385.58073
A2	-8.47972 ± 9573.41922
t2	787.27711 ± 524088.61379
Reduced Chi-Sqr	0.0229
R-Square (COD)	1
Adj. R-Square	1

Model	K(initial) Fitting of Tetra t-BuO
Equation	y = A1*exp(-x/t1) + A2*exp(-x/t2) + y0
Plot	Chloride Efflux
y0	85.1904 ± 1.32062
A1	-1.48324 ± 1.11491
t1	13.78606 ± 16.28493
A2	-85.45256 ± 0.74948
t2	143.4782 ± 6.36168
Reduced Chi-Sqr	0.24633
R-Square (COD)	0.99998
Adj. R-Square	0.99997

Model	K(initial) Fitting of DMSO - Blank
Equation	y = A1*exp(-x/t1) + A2*exp(-x/t2) + y0
Plot	Chloride Efflux
y0	-1.23633 ± 0.01479
A1	0.57865 ± 749996.29859
t1	23.55751 ± 24836.04195
A2	0.6135 ± 749996.29859
t2	23.55465 ± 23422.46594
Reduced Chi-Sqr	0.0125
R-Square (COD)	0.99001
Adj. R-Square	0.98929

Figure S44: The $k_{(initial)}$ fitting of the Cl^{-}/NO_3^{-} data sets for each receptor (**1 – 4**) (above) at 1 mol% in respect to the lipid concentration and the calculated data used to find the $k_{(initial)}$ value.

Initial Rate of Chloride Efflux seen in the Cationophore Coupled Assay and the Electrogenic Transport Character.

Table S6: The initial chloride efflux rates ($k_{initial}$, s^{-1}) measured when the cationophores, valinomycin and monensin, are added and the electrogenic transport character of the transporter.

**Transporter**	$k_{initial\ (Receptor)}$ (s^{-1}) [a]	$k_{initial\ (Vin)}$ (s^{-1}) [a]	$k_{initial\ (Mon)}$ (s^{-1}) [a]	**Electrogenic Transport Character** [b]
Tetra PhS (**1**)	0.025	0.070	0.028	2.538
Tetra PhO (**2**)	0.025	0.087	0.130	0.668
Tetra *t*-BuS (**3**)	0.166	1.845	0.168	10.955
Tetra *t*-BuO (**4**)	0.003	[c]	[c]	[c]

[a] The initial rates of chloride transport ($k_{initial}$) were calculated by fitting the chloride efflux (%) versus time (s) to a non-linear curve with an exponential decay function or fitting via linear-regression analysis.

[b] The electrogenic transport character of the transporter being tested was calculated with ($\frac{k_{initial\ (Vin)}}{k_{initial\ (Mon)}}$). When the ratio of the $k_{initial}$ values is over 1.0, electrogenic transport is preferential to electroneutral transport. Any value under 1.0 means that the mechanism of transport is predominately electroneutral.

[c] No reliable data was recorded for the tetra *t*-BuO (**4**) receptor as there was both interference with the electrode and precipitation at higher concentrations.

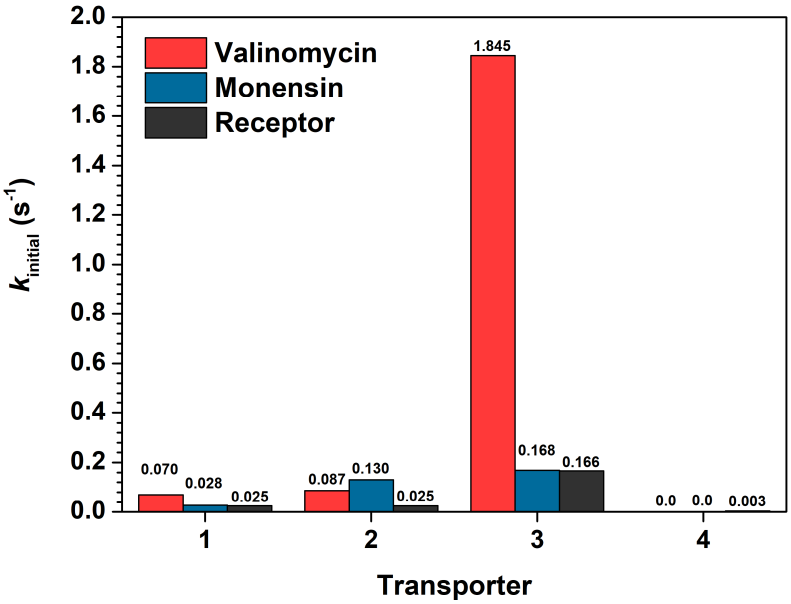

Figure S45: A comparison between the initial transport rates of chloride in the cationophore assay when the transporter (**1 – 4**) is added to a solution with either valinomycin, monensin or only DMSO (only the receptor in solution).

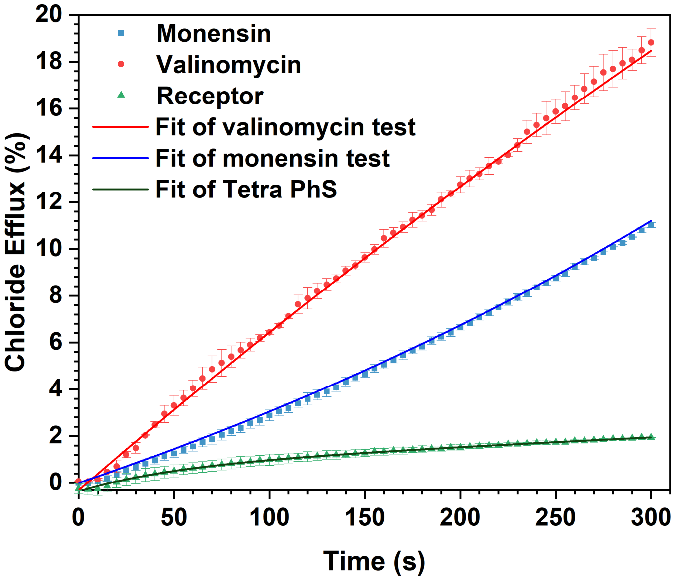

Model	K(initial) Fitting of valinomycin test
Equation	y = A1*exp(-x/t1) + A2*exp(-x/t2) + y0
Plot	Chloride Efflux
y0	14.00398 ± 4.84481
A1	-2.07738 ± 0.10652
t1	27.45835 ± 2.39655
A2	-12.42757 ± 4.75286
t2	1089.00891 ± 514.70638
Reduced Chi-Sqr	0.03525
R-Square (COD)	1
Adj. R-Square	1

Model	K(initial) Fitting of monensin test
Equation	y = A1*exp(-x/t1) + A2*exp(-x/t2) + y0
Plot	Chloride Efflux
y0	23.36443 ± 23.39024
A1	-1.31743 ± 0.09313
t1	11.54501 ± 1.84434
A2	-22.16841 ± 23.30284
t2	1370.89342 ± 1604.13697
Reduced Chi-Sqr	0.16844
R-Square (COD)	0.99998
Adj. R-Square	0.99998

Model	K(initial) Fitting of receptor test
Equation	y = A1*exp(-x/t1) + A2*exp(-x/t2) + y0
Plot	Chloride Efflux
y0	124.04381 ± 27056.05791
A1	-0.75576 ± 0.04472
t1	30.75707 ± 3.7321
A2	-123.51444 ± 27056.03149
t2	134886.63589 ± 2.95906E7
Reduced Chi-Sqr	0.00793
R-Square (COD)	1
Adj. R-Square	1

Figure S46: The $k_{(\mathrm{initial})}$ fitting of the tetra PhS (**1**) cationophore test and the calculated data used to find the $k_{(\mathrm{initial})}$ value.

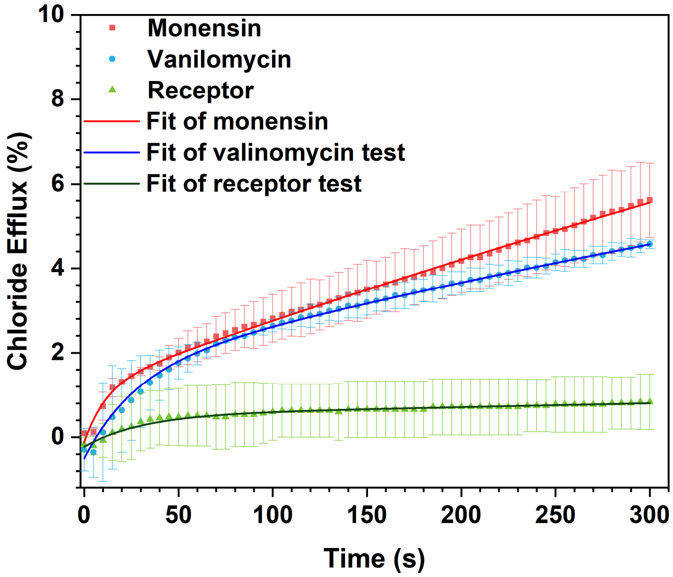

Model	K(initial) Fitting of valinomycin test
Equation	y = A1*exp(-x/t1) + A2*exp(-x/t2) + y0
Plot	Chloride Efflux
y0	14.00398 ± 4.84481
A1	-2.07738 ± 0.10652
t1	27.45835 ± 2.39655
A2	-12.42757 ± 4.75286
t2	1089.00891 ± 514.70638
Reduced Chi-Sqr	0.03525
R-Square (COD)	1
Adj. R-Square	1

Model	K(initial) Fitting of monensin test
Equation	y = A1*exp(-x/t1) + A2*exp(-x/t2) + y0
Plot	Chloride Efflux
y0	23.36443 ± 23.39024
A1	-1.31743 ± 0.09313
t1	11.54501 ± 1.84434
A2	-22.16841 ± 23.30284
t2	1370.89342 ± 1604.13697
Reduced Chi-Sqr	0.16844
R-Square (COD)	0.99998
Adj. R-Square	0.99998

Model	K(initial) Fitting of receptor test
Equation	y = A1*exp(-x/t1) + A2*exp(-x/t2) + y0
Plot	Chloride Efflux
y0	124.04381 ± 27056.05791
A1	-0.75576 ± 0.04472
t1	30.75707 ± 3.7321
A2	-123.51444 ± 27056.03149
t2	134886.63589 ± 2.95906E7
Reduced Chi-Sqr	0.00793
R-Square (COD)	1
Adj. R-Square	1

Figure S47: The $k_{(\mathrm{initial})}$ fitting of the tetra PhO (**2**) cationophore test and the calculated data used to find the $k_{(\mathrm{initial})}$ value.

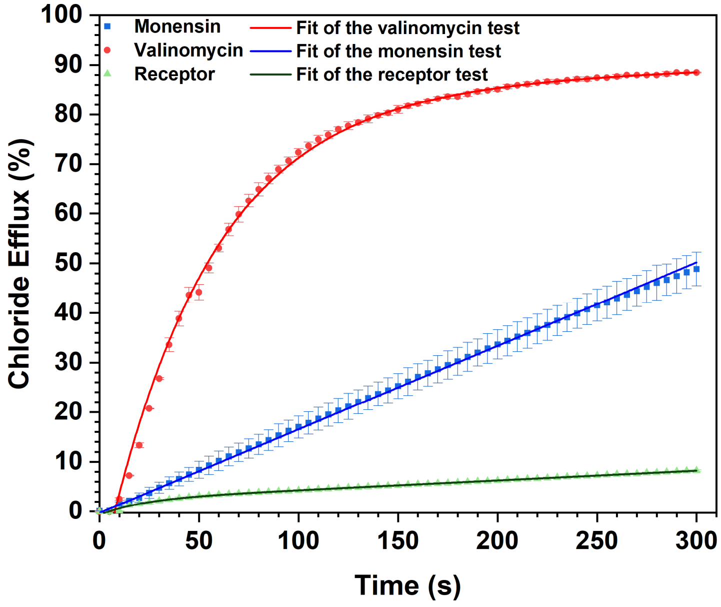

Model	K(initial) Fitting of valinomycin test
Equation	y = A1*exp(-x/t1) + A2*exp(-x/t2) + y0
Plot	Chloride Efflux
y0	689.083 ± 90289.97272
A1	-603.51595 ± 90288.83719
t1	54005.23886 ± 8119789.09612
A2	-98.76439 ± 1.56007
t2	53.86204 ± 1.52064
Reduced Chi-Sqr	2.34457
R-Square (COD)	0.99998
Adj. R-Square	0.99998

Model	K(initial) Fitting of monensin test
Equation	y = A1*exp(-x/t1) + A2*exp(-x/t2) + y0
Plot	Chloride Efflux
y0	-2895.62309 ± 5290829.46473
A1	-1369479.07568 ± 3623669.17368
t1	1280649.99127 ± 3525598.68452
A2	1372374.40232 ± 3882498.14953
t2	1523219.81437 ± 4812276.76402
Reduced Chi-Sqr	0.22414
R-Square (COD)	0.99997
Adj. R-Square	0.99997

Model	K(initial) Fitting of the receptor test
Equation	y = A1*exp(-x/t1) + A2*exp(-x/t2) + y0
Plot	Chloride Efflux
y0	2155.63427 ± 88648.01143
A1	-3.1019 ± 0.14575
t1	21.17273 ± 2.61624
A2	-2153.2772 ± 88647.87938
t2	109828.08812 ± 4529494.33976
Reduced Chi-Sqr	0.7554
R-Square (COD)	0.99992
Adj. R-Square	0.99992

Figure S48: The $k_{(\mathrm{initial})}$ fitting of the tetra *t*-BuS (**3**) cationophore test and the calculated data used to find the $k_{(\mathrm{initial})}$ value.

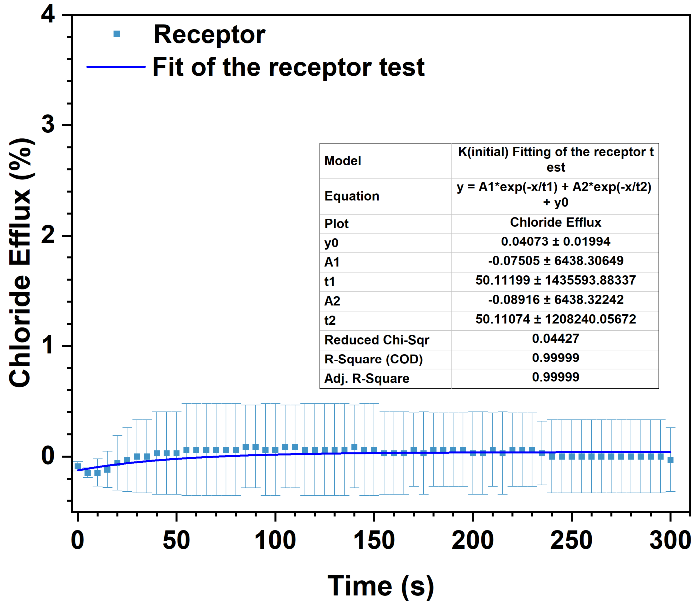

Model	K(initial) Fitting of the receptor test
Equation	y = A1*exp(-x/t1) + A2*exp(-x/t2) + y0
Plot	Chloride Efflux
y0	0.04073 ± 0.01994
A1	-0.07505 ± 6438.30649
t1	50.11199 ± 1435593.88337
A2	-0.08916 ± 6438.32242
t2	50.11074 ± 1208240.05672
Reduced Chi-Sqr	0.04427
R-Square (COD)	0.99999
Adj. R-Square	0.99999

Figure S49: The $k_{(\mathrm{initial})}$ fitting of the tetra *t*-BuO (**4**) cationophore test and the calculated data used to find the $k_{(\mathrm{initial})}$ value.

The partition coefficient (LogP).

Estimated Partition Coefficient Values:

Table S7: The estimated partition coefficients (LogP) for the tetrapodal transporters.

**Transporter**	**Partition Coefficient (LogP)**
Tetra PhS (**1**)	4.11
Tetra PhO (**2**)	3.09
Tetra *t*-BuS (**3**)	3.32
Tetra t-BuO (**4**)	1.65

The estimation of the partition coefficients was performed on the online app ALOGPS 2.1 via the Virtual Computational Chemistry Laboratory website [6,7].

S5. ^{1}H-NMR Binding Studies:

S5.1 Anion Binding Studies:

Binding Constants (K_a), β_{12} and the Covariance of Fit:

Table S8: A comparison between the binding constants (K_a) for receptor:anion complexation with different anions produced by the change in receptor design.

Tetrapodal Receptors	K_a Cl^- [M^{-1}]	K_a NO_3^- [M^{-1}]	K_a SO_4^{2-} [M^{-1}]	K_a HCO_3^- [M^{-1}] [h]	K_a $H_2PO_4^-$ [M^{-1}]	K_a $HP_2O_7^{3-}$ [M^{-1}]
Tetra PhS (**1**)	K_{11}: 458, K_{12}: 30	n.d.[a]	K_{11}: 1000, K_{21}: 56	[b]	[b]	[c]
Tetra PhO (**2**)	K_{11}: 447, K_{12}: 28	n.d.[a]	[b]	[b]	[b]	[c]
Tetra *t*-BuS (**3**)	K_{11}: 241, K_{12}: 16	n.d.[a]	> 10^4	K_{11}: 1800, K_{12}: 305	[b]	[d]
Tetra *t*-BuO (**4**)	K_{11}: 305, K_{12}: 7	n.d.[a]	[b]	K_{11}: 559, K_{12}: 21	[b]	4160
**Tripodal Receptors**						
Tris PhS	K_{11}: 1800, K_{12}: 10 [f]	n.d.[a, f]	> 10^4 [f]	[d, f]	K_{11}: 1200, K_{12}: 17 [f]	[e]
Tris PhO	K_{11}: 1300, K_{12}: 2.5 [f]	n.d.[a, f]	> 10^4 [f]	K_{11}: 3×10^4, K_{12}: 23 [f]	K_{11}: 5×10^4, K_{12}: 22 [f]	[e]
Tris *t*-BuS	K_a: 1110 [g]	[e, g]	[b,g]	2670 [g]	2600 [g]	[e]

[a] No anion binding interactions observed. [b] Complex binding and deprotonation of the receptor occurred. [c] Fast and slow exchange occurring. [d] The experimental data could not be fit to 1:1, 1:2 or 2:1 binding models. [e] The anion was not tested for this receptor. [f] The binding constants were determined in an earlier study by Jowett *et al*.[4] [g] The binding constants were determined in an earlier study by Jowett *et al*.[5] [h] The anion was added as the tetraethylammonium (TEA) salt.

Table S9: The total 1:2 binding constant (β_{12}), the covariance of fit (cov_{fit}) for the 1:1, 1:2 and 2:1 binding models and the enhancement factor of the covariance of fit ($F_{(covfit)}$).

Receptor	Anion	β_{12} [M^{-2}] [a]	1:1 cov_{fit} [b]	1:2 cov_{fit} [b]	$F_{(covfit)}$ [c]
Tetra PhS (**1**)	Cl^-	1.36×10^4	1.73×10^{-3}	1.51×10^{-3}	1.14
	SO_4^{2-}	5.59×10^4 [d]	3.37×10^{-3}	7.18×10^{-4} [d]	13.9
Tetra PhO (**2**)	Cl^-	1.27×10^4	1.27×10^{-3}	4.14×10^{-4}	3.06
Tetra *t*-BuS (**3**)	Cl^-	3.90×10^3	1.03×10^{-4}	2.89×10^{-6}	35.8
	HCO_3^-	5.05×10^5	1.17×10^{-3}	6.36×10^{-6}	184
Tetra *t*-BuO (**4**)	Cl^-	2.02×10^4	1.45×10^{-4}	2.36×10^{-5}	6.16
	HCO_3^-	1.19×10^4	2.07×10^{-4}	6.05×10^{-5}	3.42
	$HP_2O_7^{3-}$	9.29×10^4	4.58×10^{-4}	4.50×10^{-4}	1.02

[a] The overall binding contant of 1:2 binding (β_{12}) was calculated by multiplication of $K_{11} \times K_{12}$ in table S6. [b] The covariance of fit (1:1, 1:2 cov_{fit}) was calculated by calculating the covariance of the residual (subtracting the experimental data from the calculated data produced from the fitting of proton shifts). The cov_{fit} was then calculated by division of the calculated fitted covariance by the covariance of the residual (cov_{cal}/cov_{res}). [c] The $F_{(covfit)}$ enhancement factor was calculated by division of 1:1 cov_{fit} by 1:2 cov_{fit} (1:1 cov_{fit}/1:2 cov_{fit}).[8] [d] The β value and covariance of fit for the sulfate test on tetra PhS (**1**) was calculated using the 2:1 binding model data in exactly the same manner as the 1:2 calculations.

*Tetra PhS (**1**) Titration Spectra:*

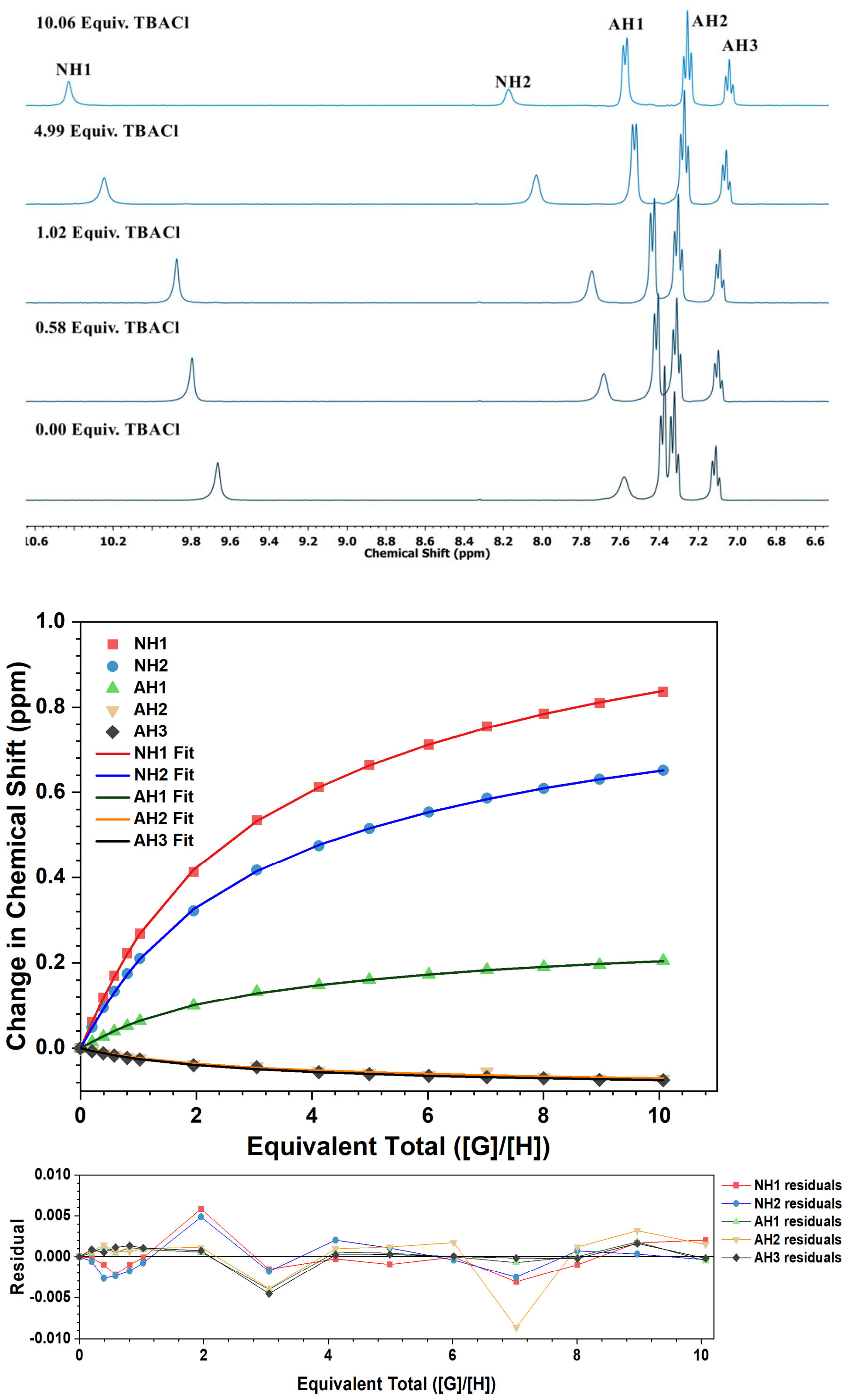

Figure S50: The ^{1}H-NMR stacked plot of tetra PhS (**1**) (5 mM) titrated with TBACl in DMSO-d_6/0.5% H_2O at 298 K (above). The fit plot of 1:2 receptor:anion binding and the residual error obtained from fitting to the 1:2 binding isotherm K_{11} = 458, K_{12} = 30 with K_{11} error = 2.96% and K_{12} error = 1.33% (below).

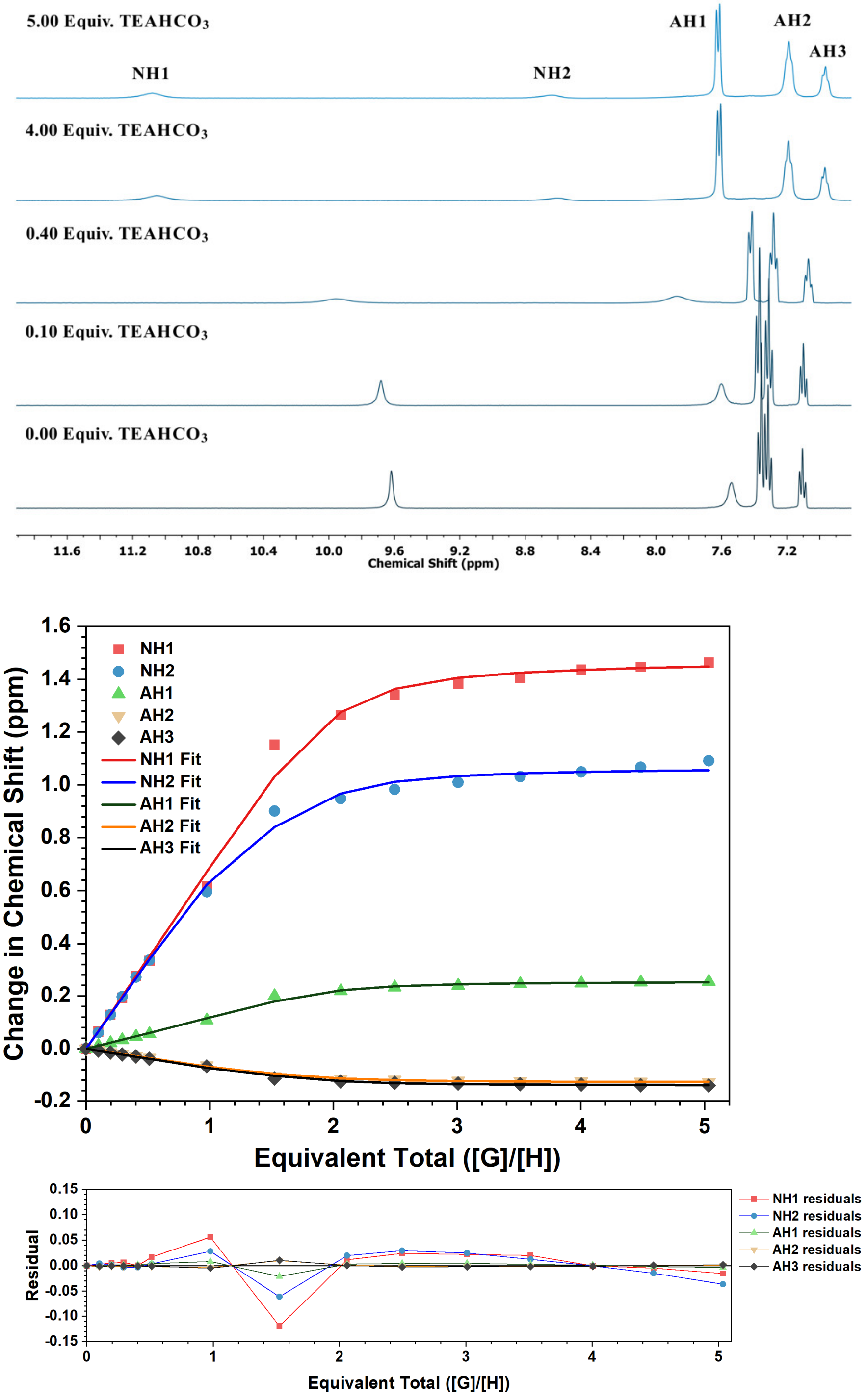

Figure S51: The ^{1}H-NMR stacked plot of tetra PhS (**1**) (5 mM) titrated with $TEAHCO_3$ in DMSO-d_6/0.5% H_2O at 298 K (above). The fit plot of 1:2 receptor:anion binding and the residual error obtained from fitting to the 1:2 binding isotherm (below). No binding constants were obtained due to poor fitting and high error with K_{11} error = 172.23% and K_{12} error = 13.87%.

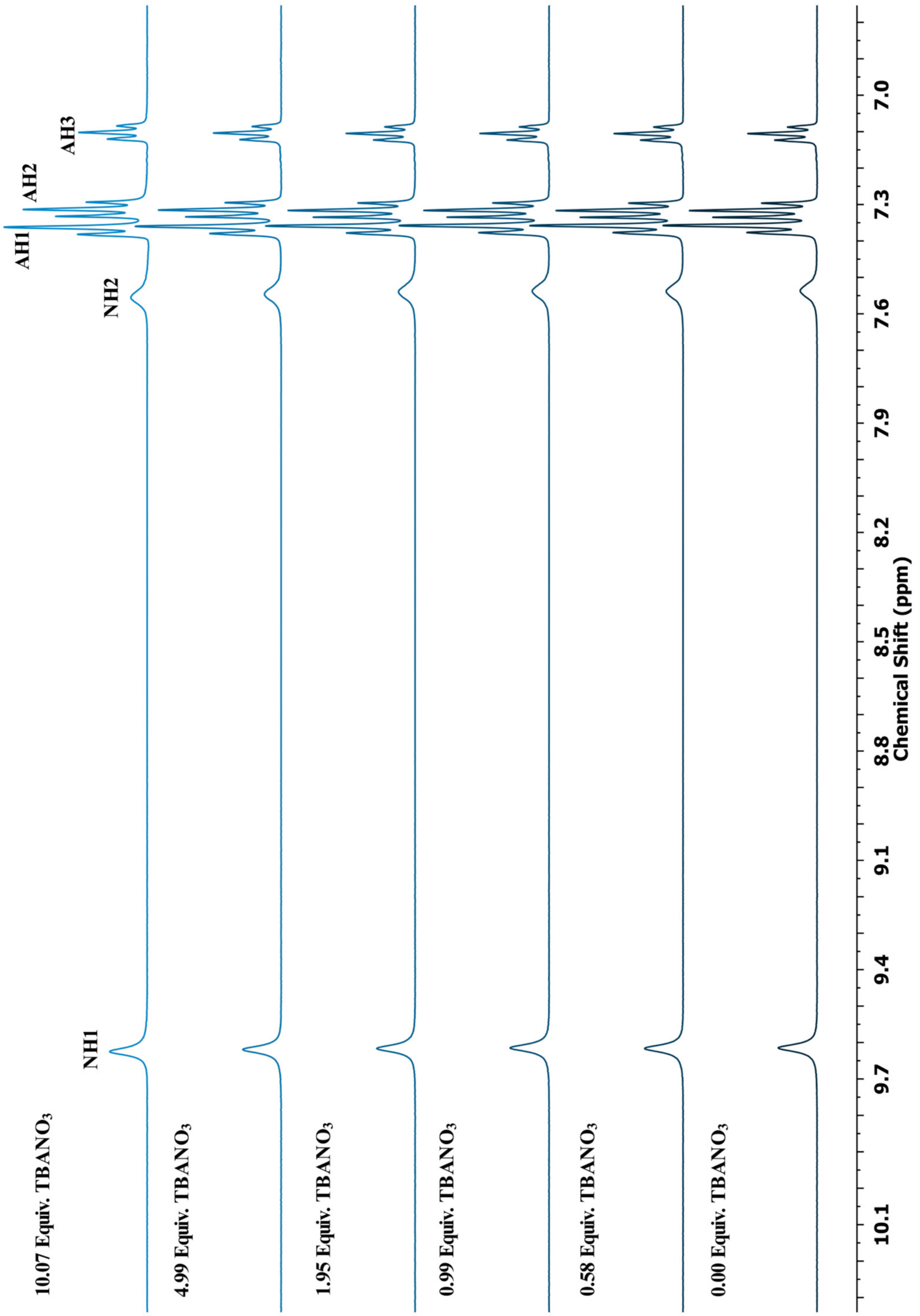

Figure S52: The ^{1}H-NMR stacked plot of tetra PhS (**1**) (5 mM) titrated with $TBANO_3$ in DMSO-d_6/0.5% H_2O at 298 K. There was no observed shifting from the protons so no binding isotherm graphs were made and no binding constants were found.

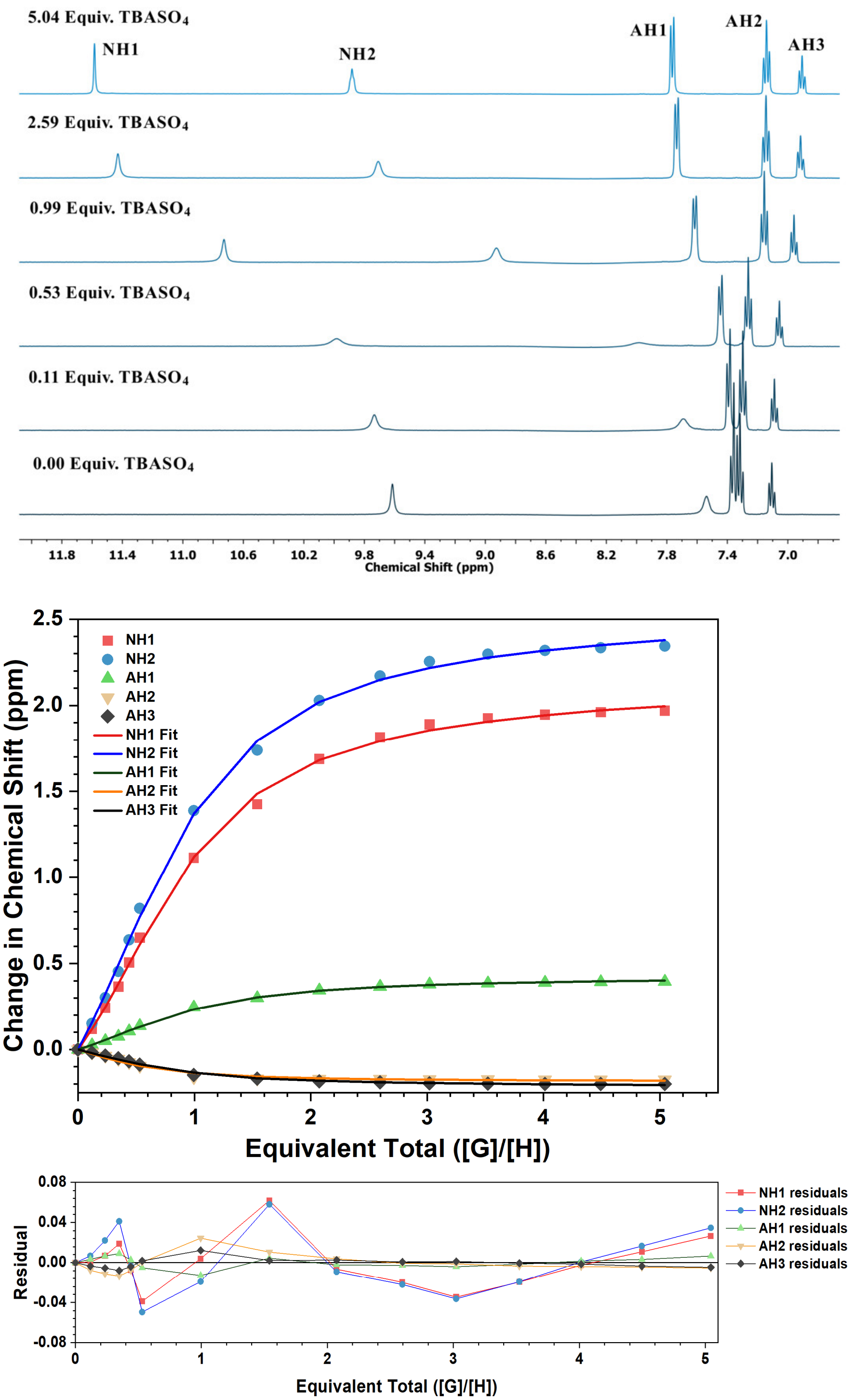

Figure S53: The ^{1}H-NMR stacked plot of tetra PhS (**1**) (5 mM) titrated with TBA_2SO_4 in DMSO-d_6/0.5% H_2O at 298 K (above). The fit plot of 2:1 receptor:anion binding and the residual error obtained from fitting to the 2:1 binding isotherm K_{11} = 1005, K_{21} = 56 with K_{11} error = 6.86% and K_{21} error = 10.88% (below).

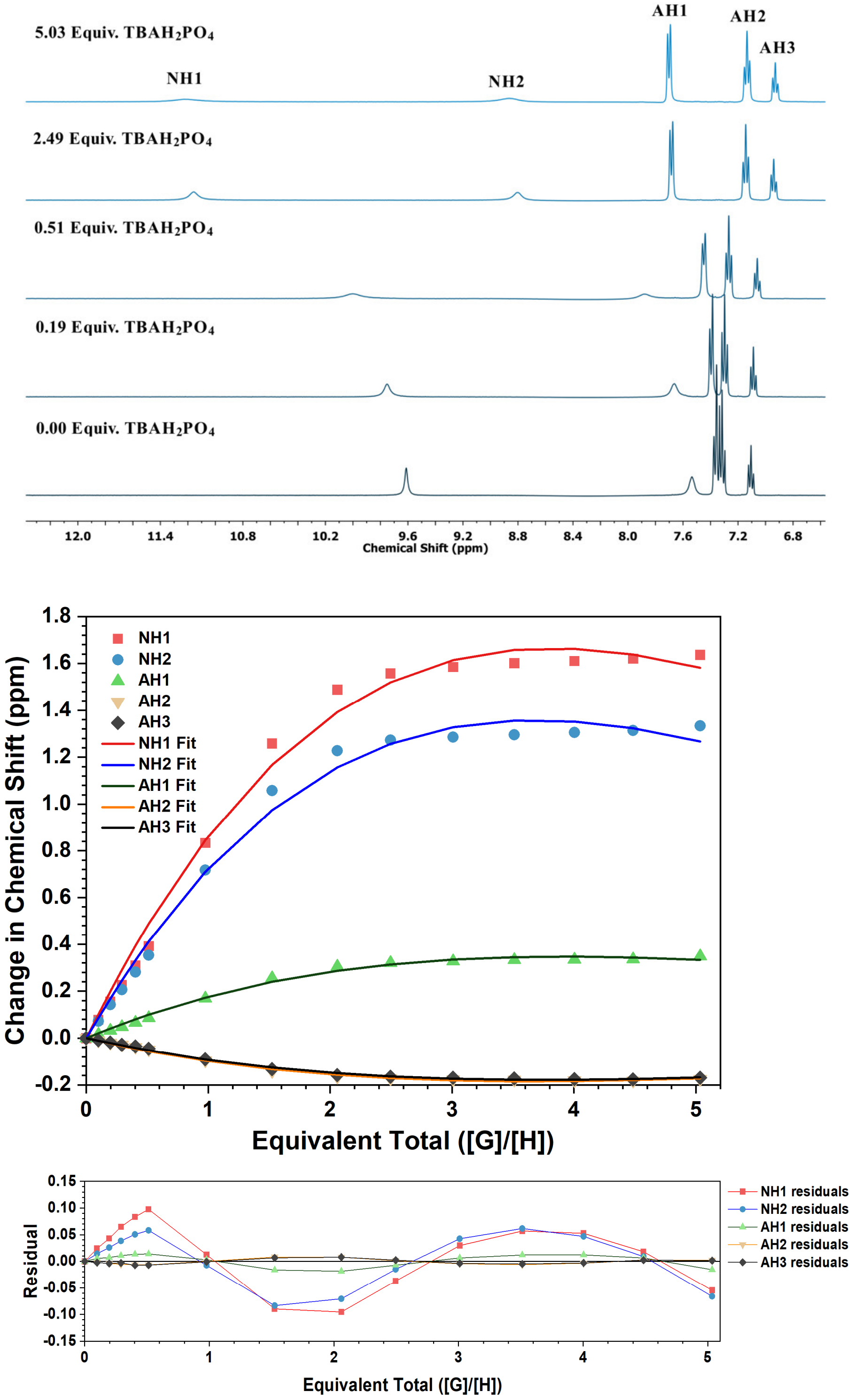

Figure S54: The ^{1}H-NMR stacked plot of tetra PhS (**1**) (5 mM) titrated with $TBAH_2PO_4$ in DMSO-d_6/0.5% H_2O at 298 K (above). The fit plot of 1:2 receptor:anion binding and the residual error obtained from fitting to the 1:2 binding isotherm (below). No binding constants were obtained due to poor fitting with K_{11} error = 3.87% and K_{12} error = 4.3%.

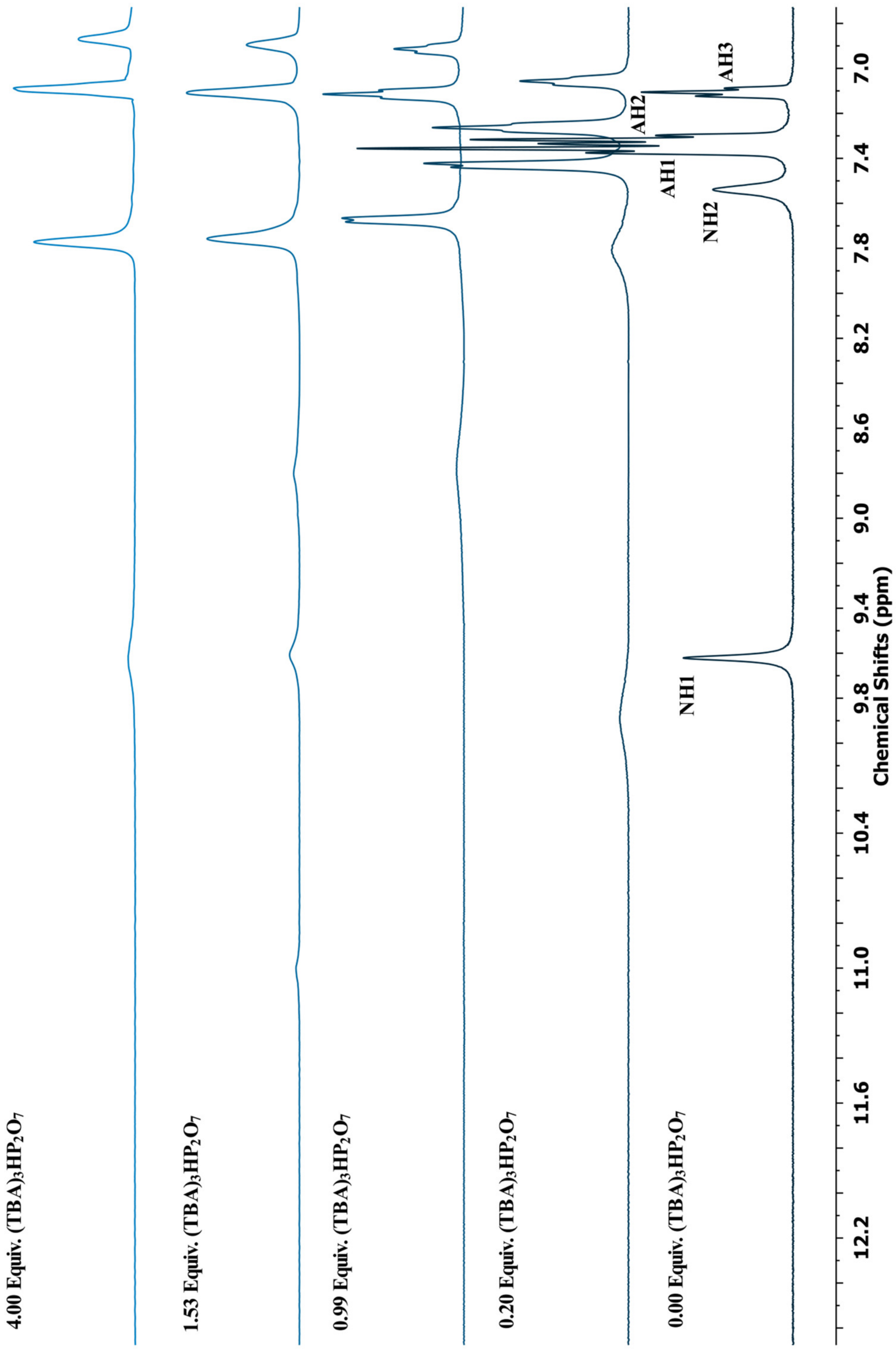

Figure S55: The ^{1}H-NMR stacked plot of tetra PhS (**1**) (5 mM) titrated with $(TBA)_3HP_2O_7$ in DMSO-d_6/0.5% H_2O at 298 K. Due to complex binding cause by fast and slow exchange no binding constants were obtained.

Tetra PhO (**2**) *Titration Spectra:*

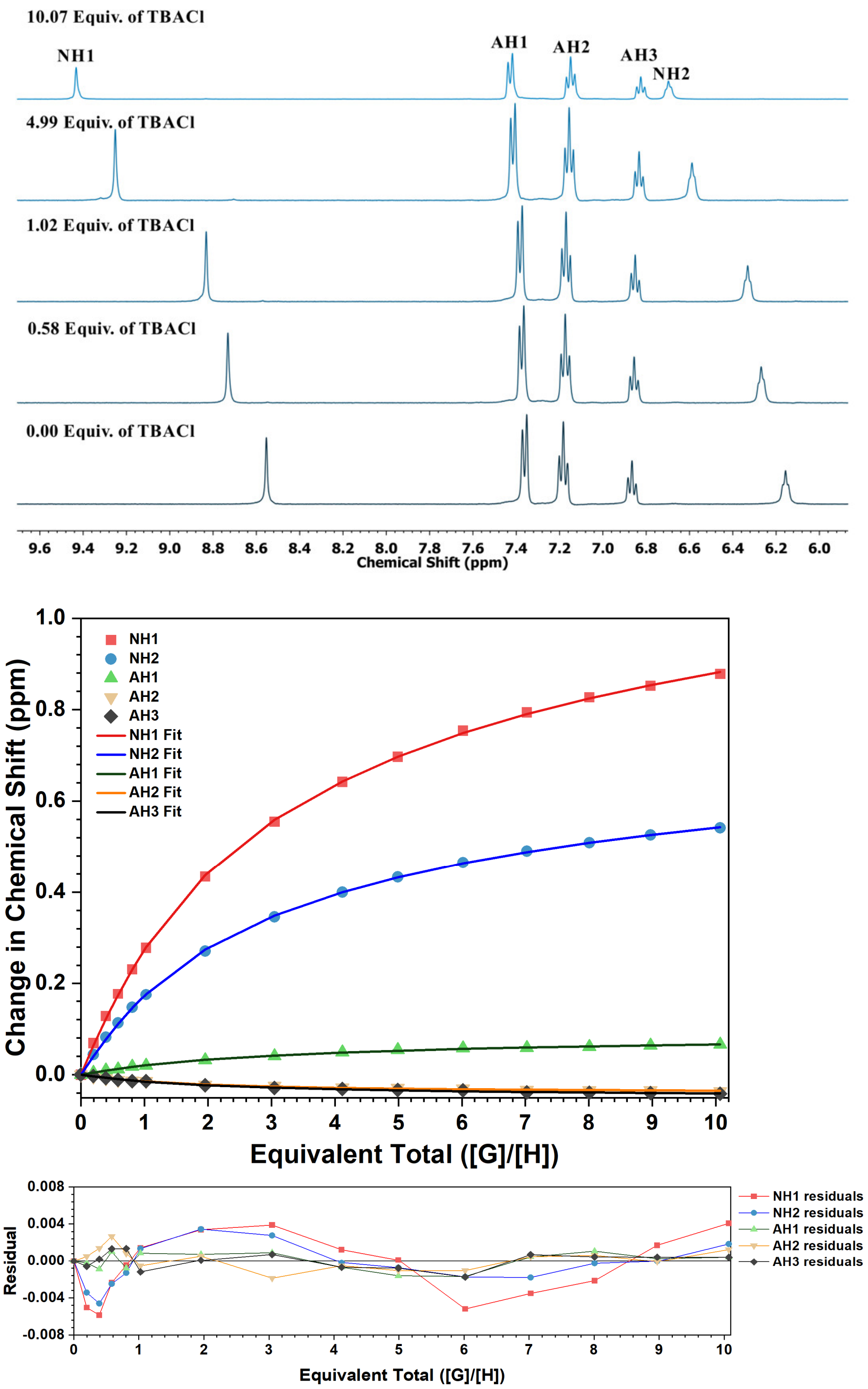

Figure S56: The ^{1}H-NMR stacked plot of tetra PhO (**2**) (5 mM) titrated with TBACl in DMSO-d_6/0.5% H_2O at 298 K (above). The fit plot of 1:2 receptor:anion binding and the residual error obtained from fitting to the 1:2 binding isotherm K_{11} = 447, K_{12} = 28 with K_{11} error = 2.04% and K_{12} error = 0.79% (below).

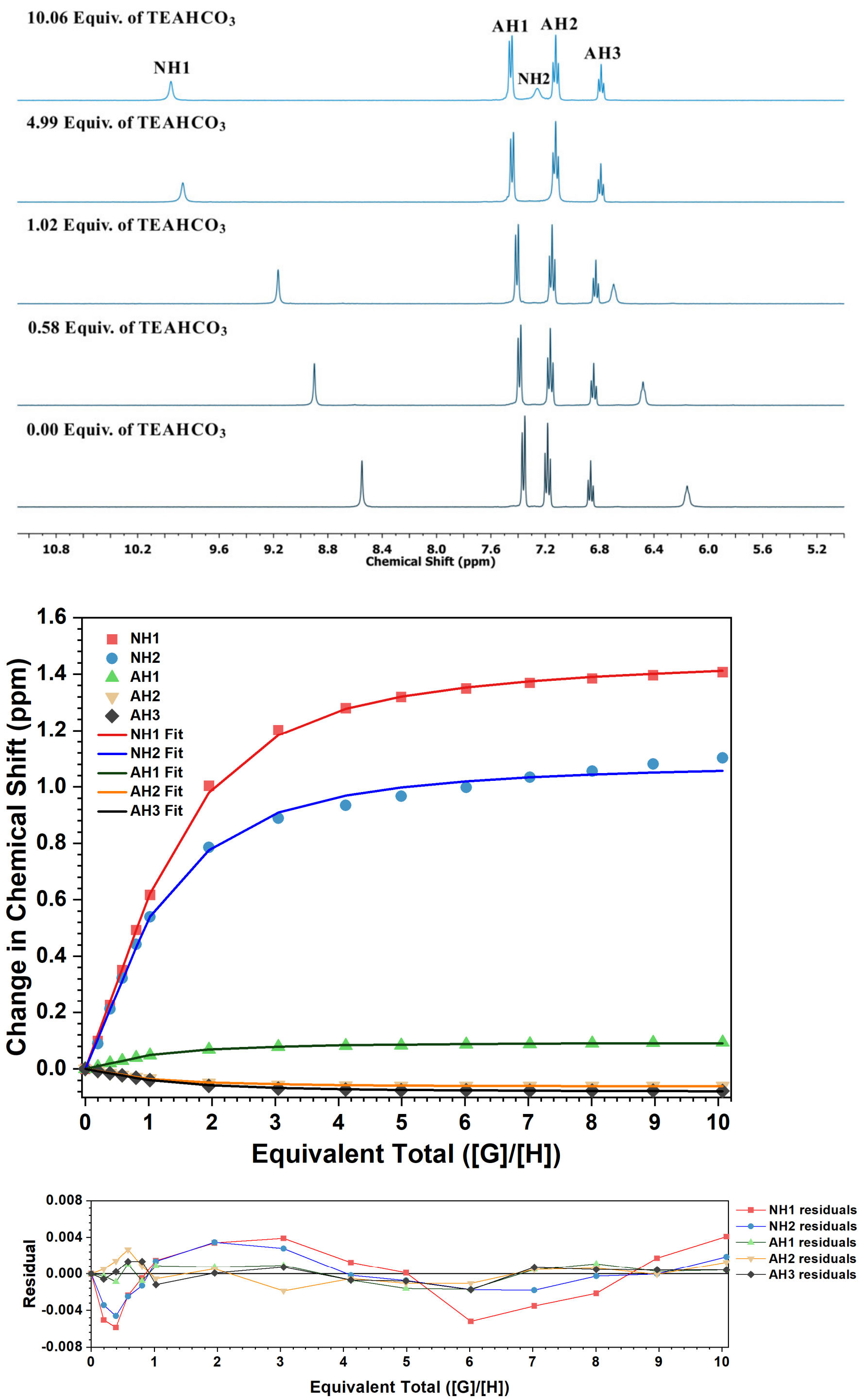

Figure S57: The ^{1}H-NMR stacked plot of tetra PhO (**2**) (5 mM) titrated with $TEAHCO_3$ in DMSO-d_6/0.5% H_2O at 298 K (above). The fit plot of 1:2 receptor:anion binding and the residual error obtained from fitting to the 1:2 binding isotherm (below). No binding constants were obtained due to poor fitting from complex binding with K_{11} error = 753.15% and K_{12} error = 5.59%.

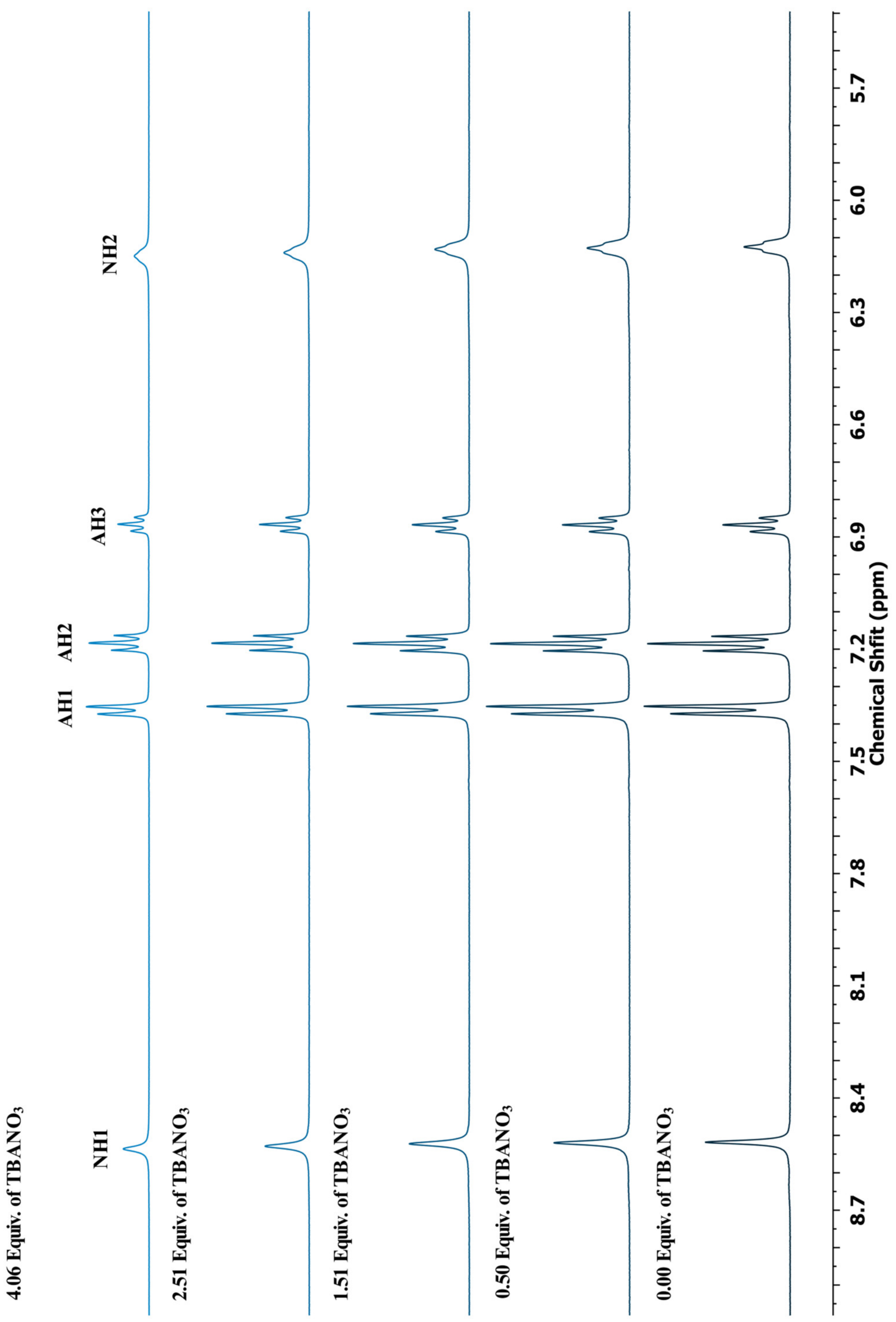

Figure S58: The ^{1}H-NMR stacked plot of tetra PhO (**2**) (5 mM) titrated with $TBANO_3$ in DMSO-d_6/0.5% H_2O at 298 K. There was no observed shifting from the protons so no binding isotherm graphs were made and no binding constants were found.

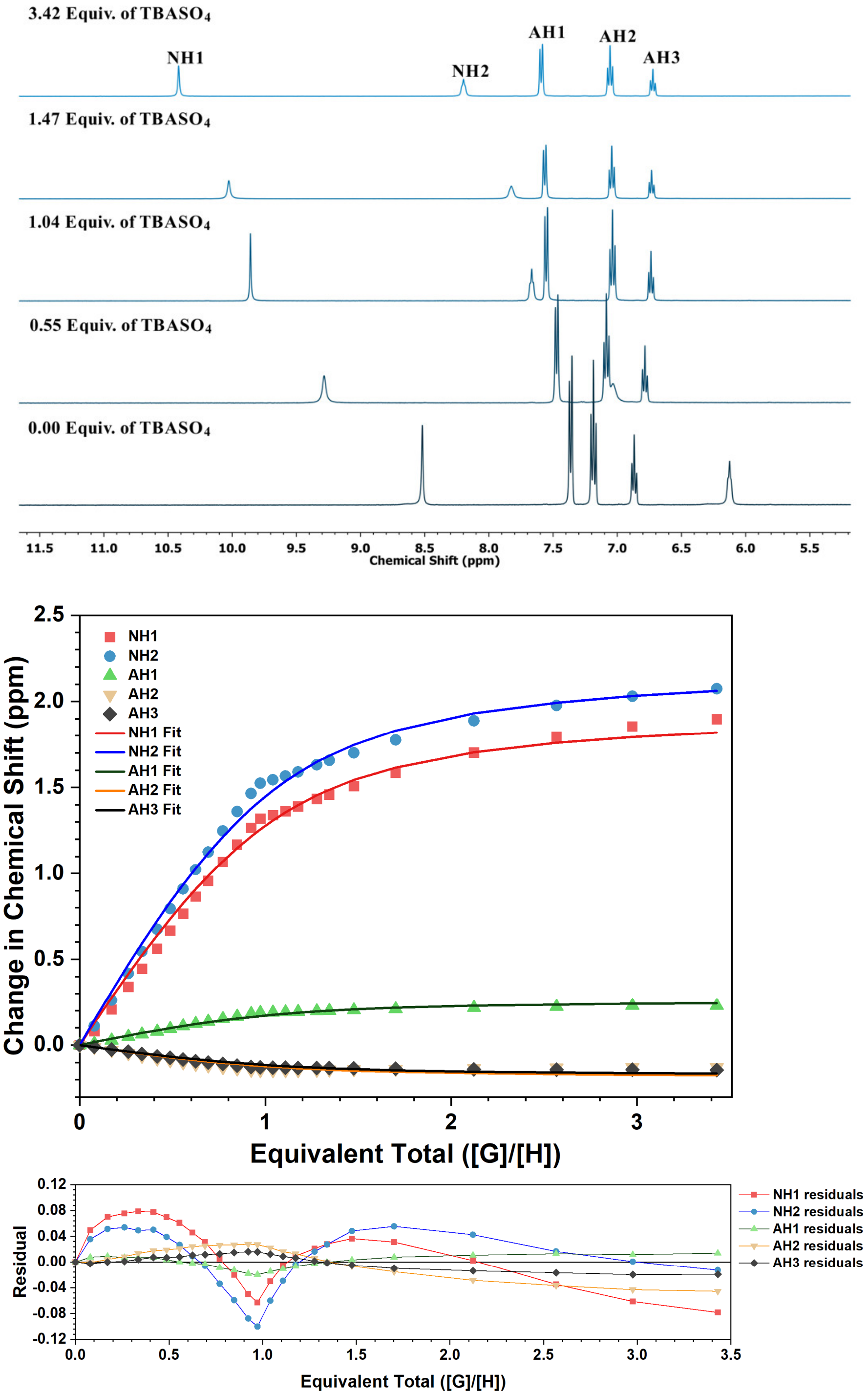

Figure S59: The ^{1}H-NMR stacked plot of tetra PhO (**2**) (5 mM) titrated with TBA_2SO_4 in DMSO-d_6/0.5% H_2O at 298 K (above). The fit plot of 1:1 receptor:anion binding and the residual error obtained from fitting to the 1:1 binding isotherm (below). No binding constants were obtained due to poor fitting from complex binding with K_a error = 4.33%.

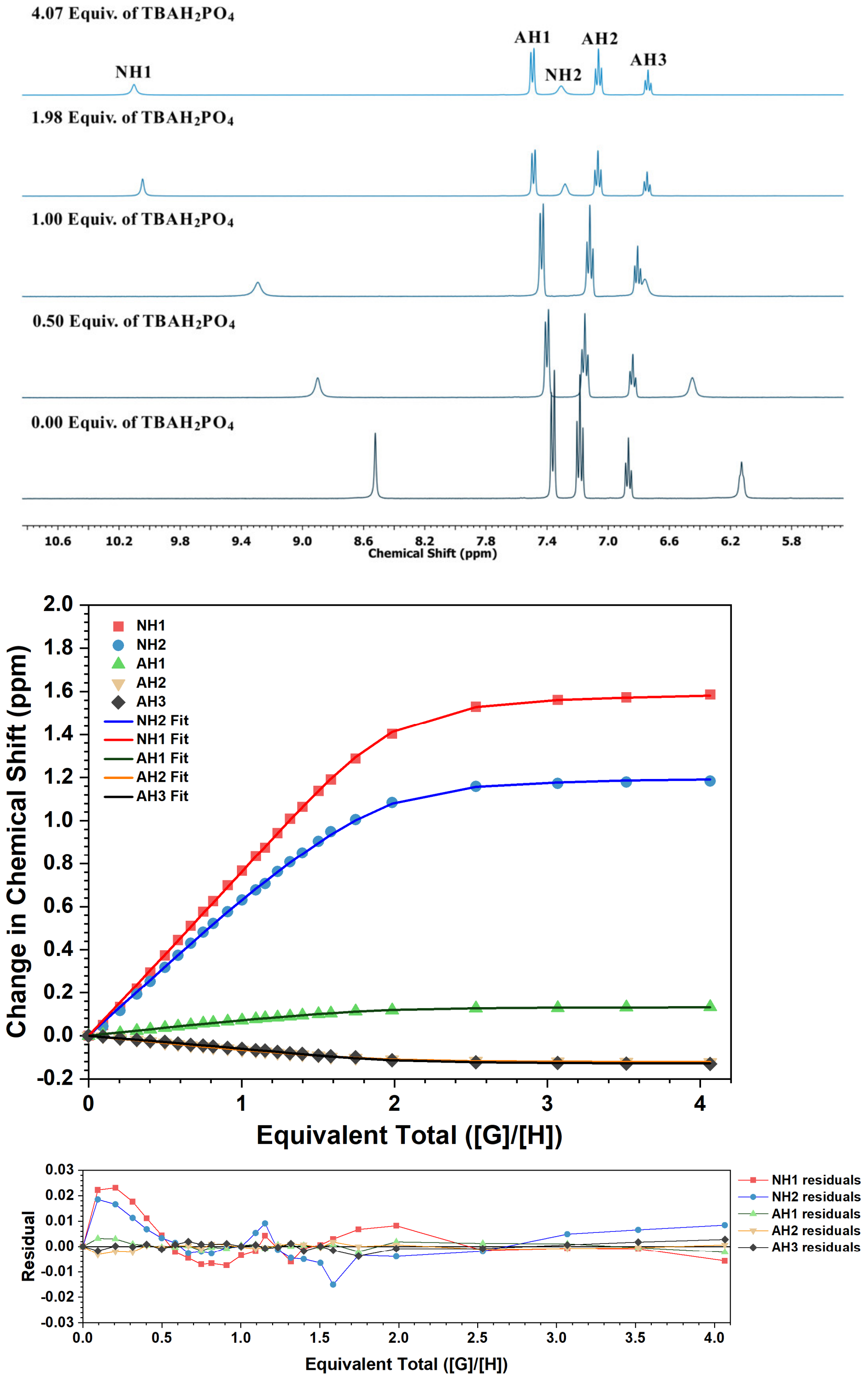

Figure S60: The ^{1}H-NMR stacked plot of tetra PhO (**2**) (5 mM) titrated with $TBAH_2PO_4$ in DMSO-d_6/0.5% H_2O at 298 K (above). The fit plot of 1:2 receptor:anion binding and the residual error obtained from fitting to the 1:2 binding isotherm (below). No binding constants were obtained due to poor fitting with K_{11} error = 47.26% and K_{12} error = 6.41%.

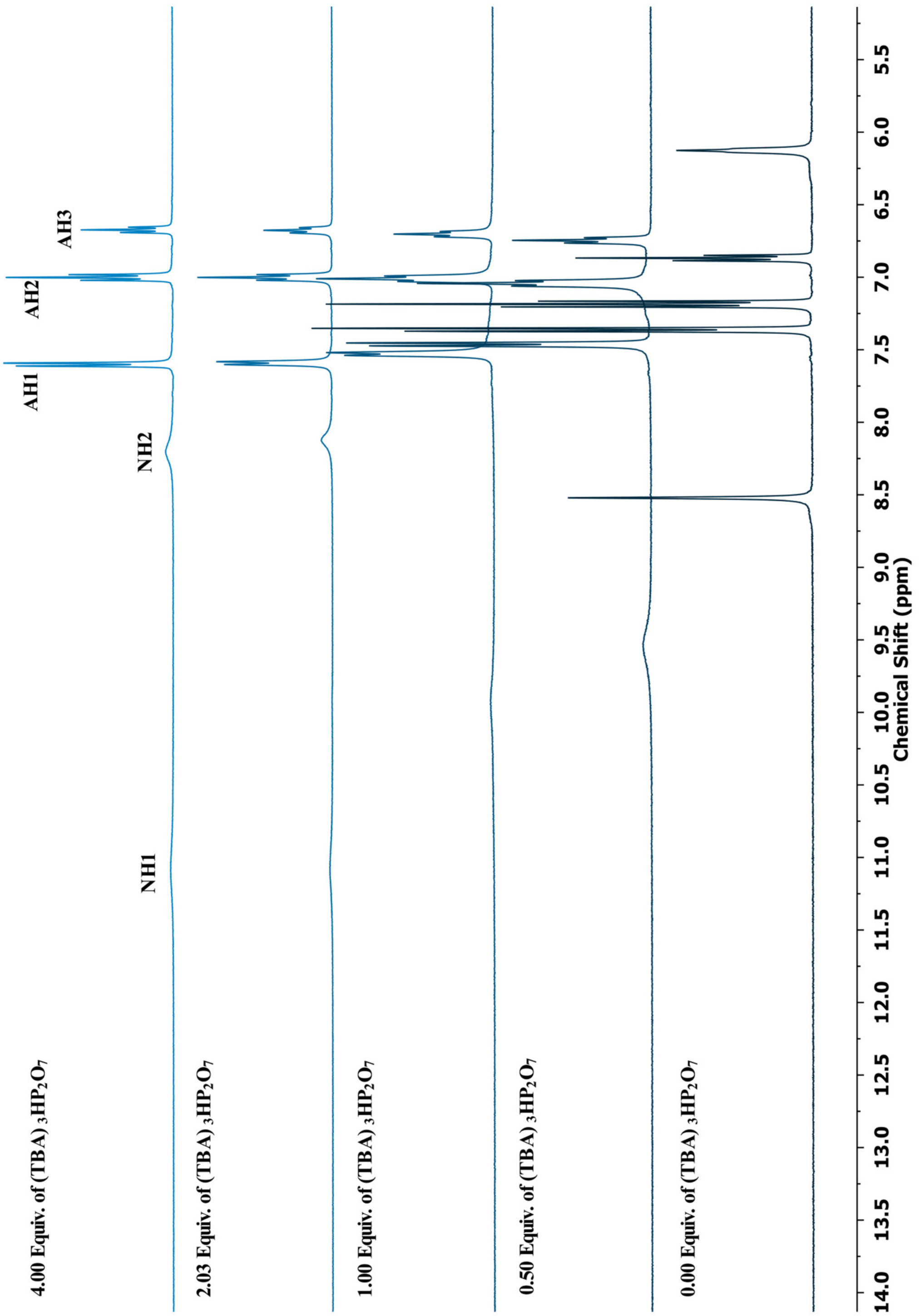

Figure S61: The ^{1}H-NMR stacked plot of tetra PhO (**2**) (5 mM) titrated with $(TBA)_3HP_2O_7$ in DMSO-d_6/0.5% H_2O at 298 K. Due to complex binding cause by fast and slow exchange no binding constants were obtained.

Tetra t-BuS (3) Titration Spectra:

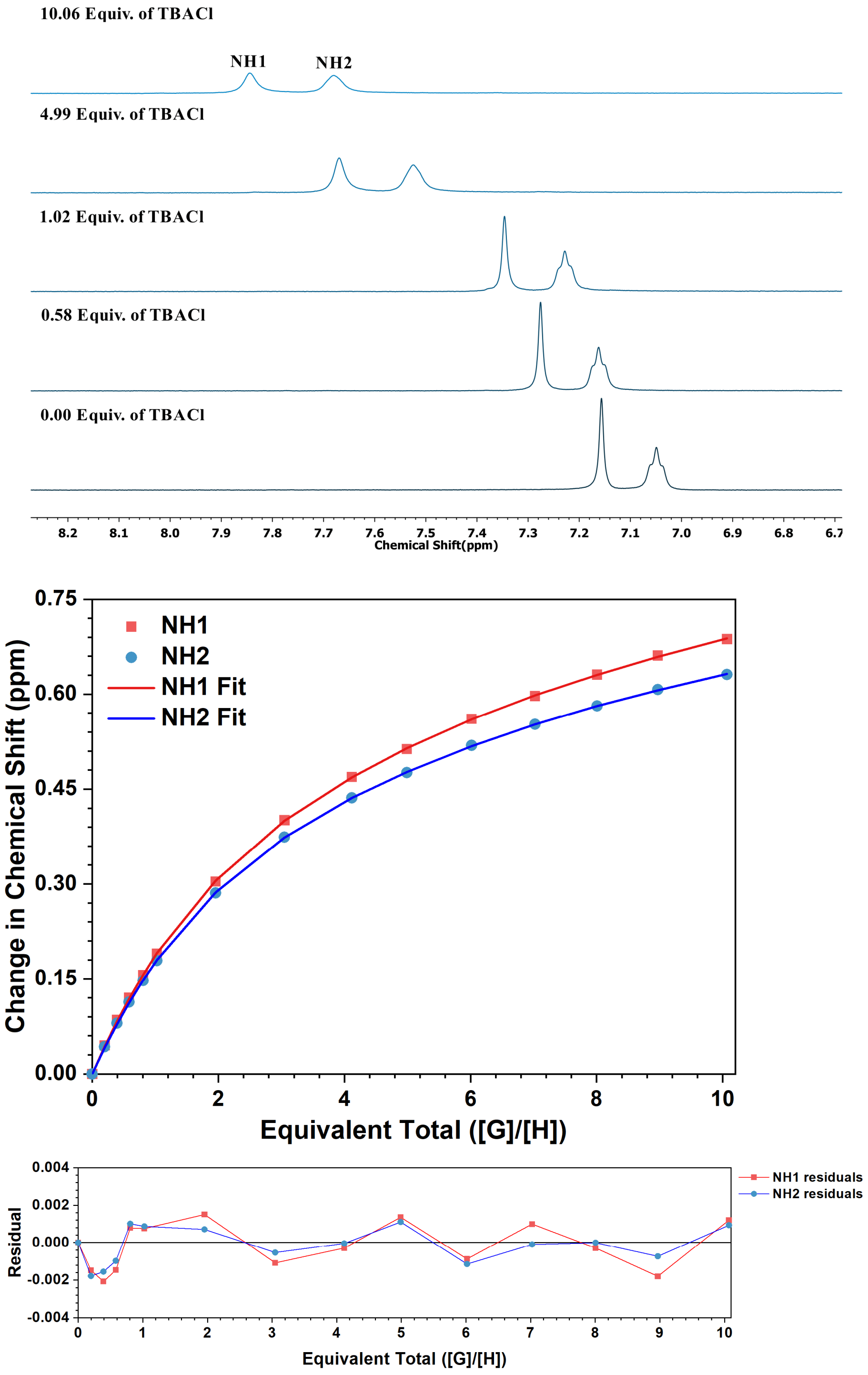

Figure S62: The ^{1}H-NMR stacked plot of tetra *t*-BuS (**3**) (5 mM) titrated with TBACl in DMSO-d_6/0.5% H_2O at 298 K (above). The fit plot of 1:2 receptor:anion binding and the residual error obtained from fitting to the 1:2 binding isotherm K_{11} = 241, K_{12} = 16 with K_{11} error = 1.25% and K_{12} error = 0.71% (below).

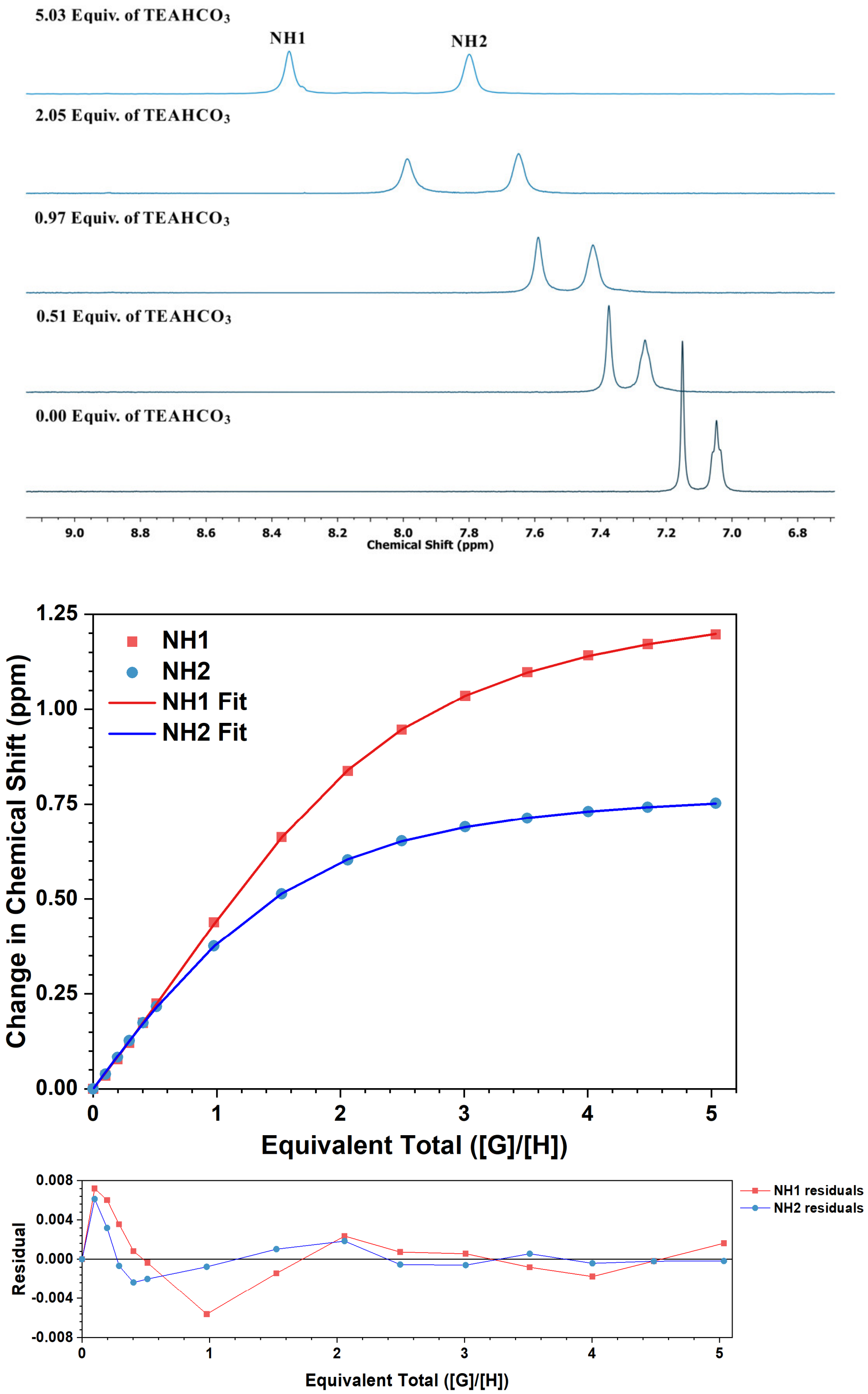

Figure S63: The ^{1}H-NMR stacked plot of tetra *t*-BuS (**3**) (5 mM) titrated with $TEAHCO_3$ in DMSO-d_6/0.5% H_2O at 298 K (above). The fit plot of 1:2 receptor:anion binding and the residual error obtained from fitting to the 1:2 binding isotherm $K_{11} = 1802$, $K_{12} = 305$ with K_{11} error = 6.69% and K_{12} error = 1.72% (below).

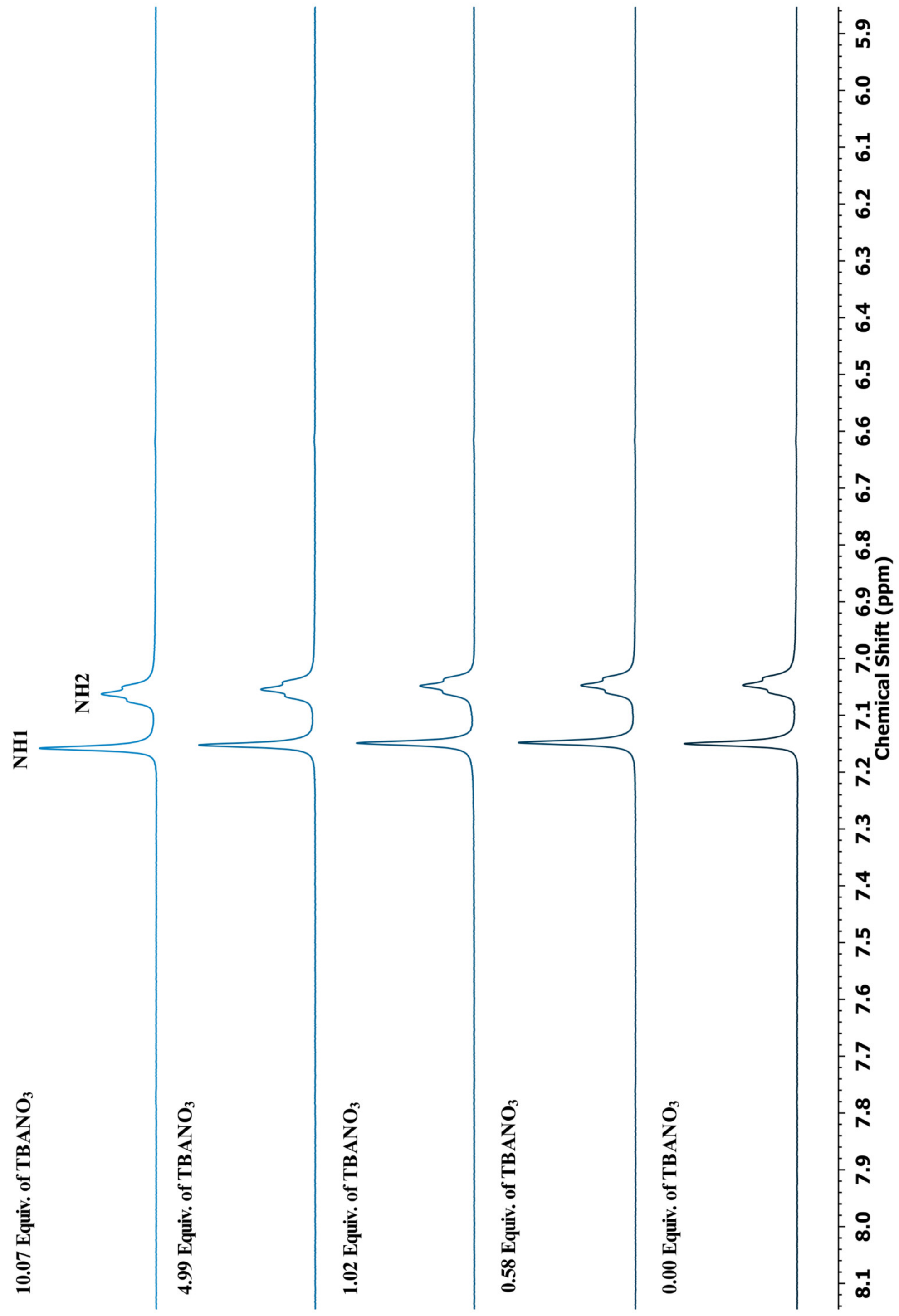

Figure S64: The ^{1}H-NMR stacked plot of tetra *t*-BuS (**3**) (5 mM) titrated with $TBANO_3$ in DMSO-d_6/0.5% H_2O at 298 K. There was no observed shifting from the protons so no binding isotherm graphs were made and no binding constants were found.

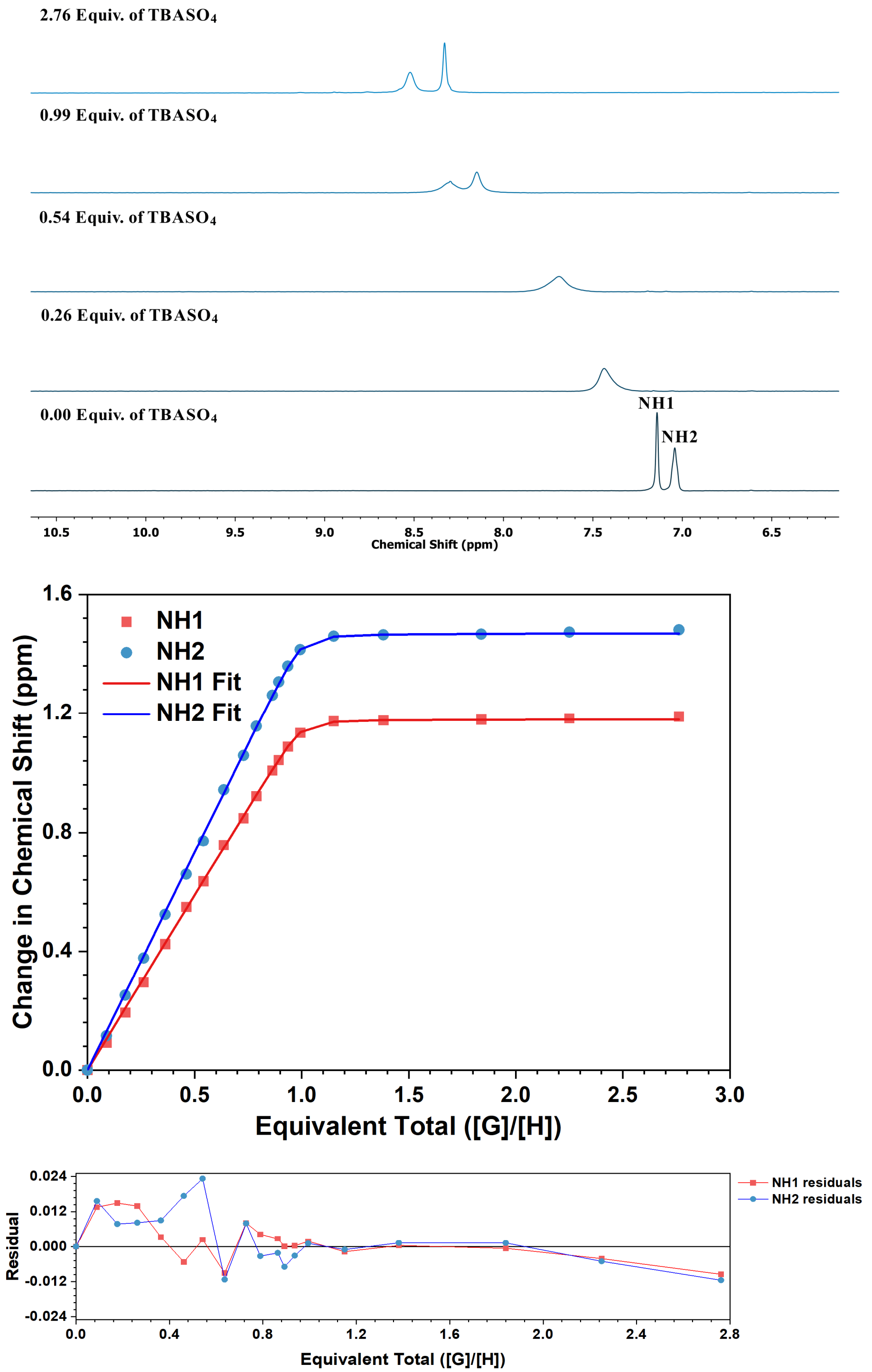

Figure S65: The ^{1}H-NMR stacked plot of tetra *t*-BuS (**3**) (5 mM) titrated with TBA_2SO_4 in DMSO-d_6/0.5% H_2O at 298 K (above). The fit plot of 1:1 receptor:anion binding and the residual error obtained from fitting to the 1:1 binding isotherm K_a > 10^4 with error = 37.9% (below).

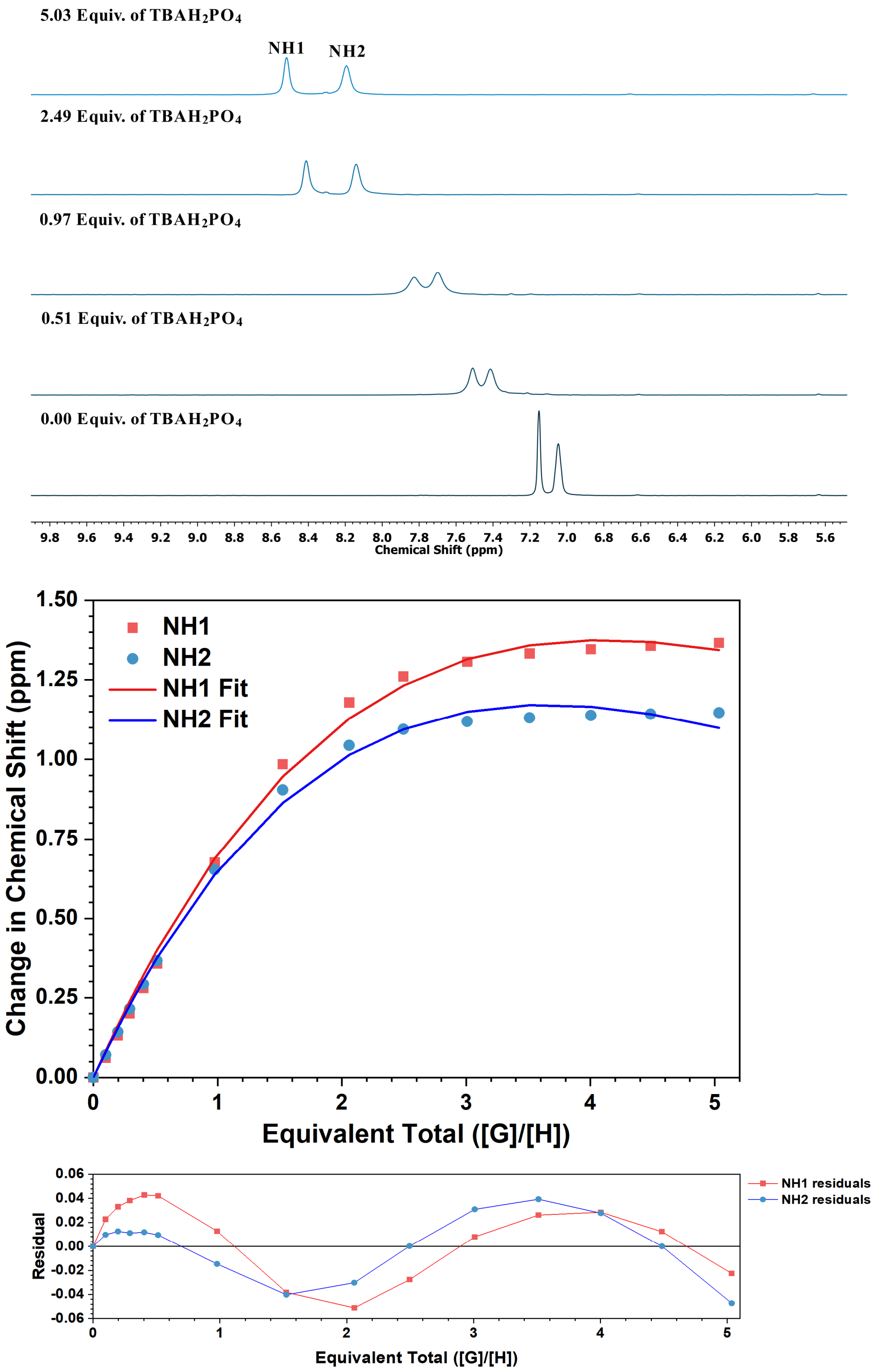

Figure S66: The ^{1}H-NMR stacked plot of tetra *t*-BuS (**3**) (5 mM) titrated with $TBAH_2PO_4$ in DMSO-d_6/0.5% H_2O at 298 K (above). The fit plot of 1:2 receptor:anion binding and the residual error obtained from fitting to the 1:2 binding isotherm (below). No binding constants were obtained due to poor fitting with K_{11} error = 3.40% and K_{12} error = 2.16%.

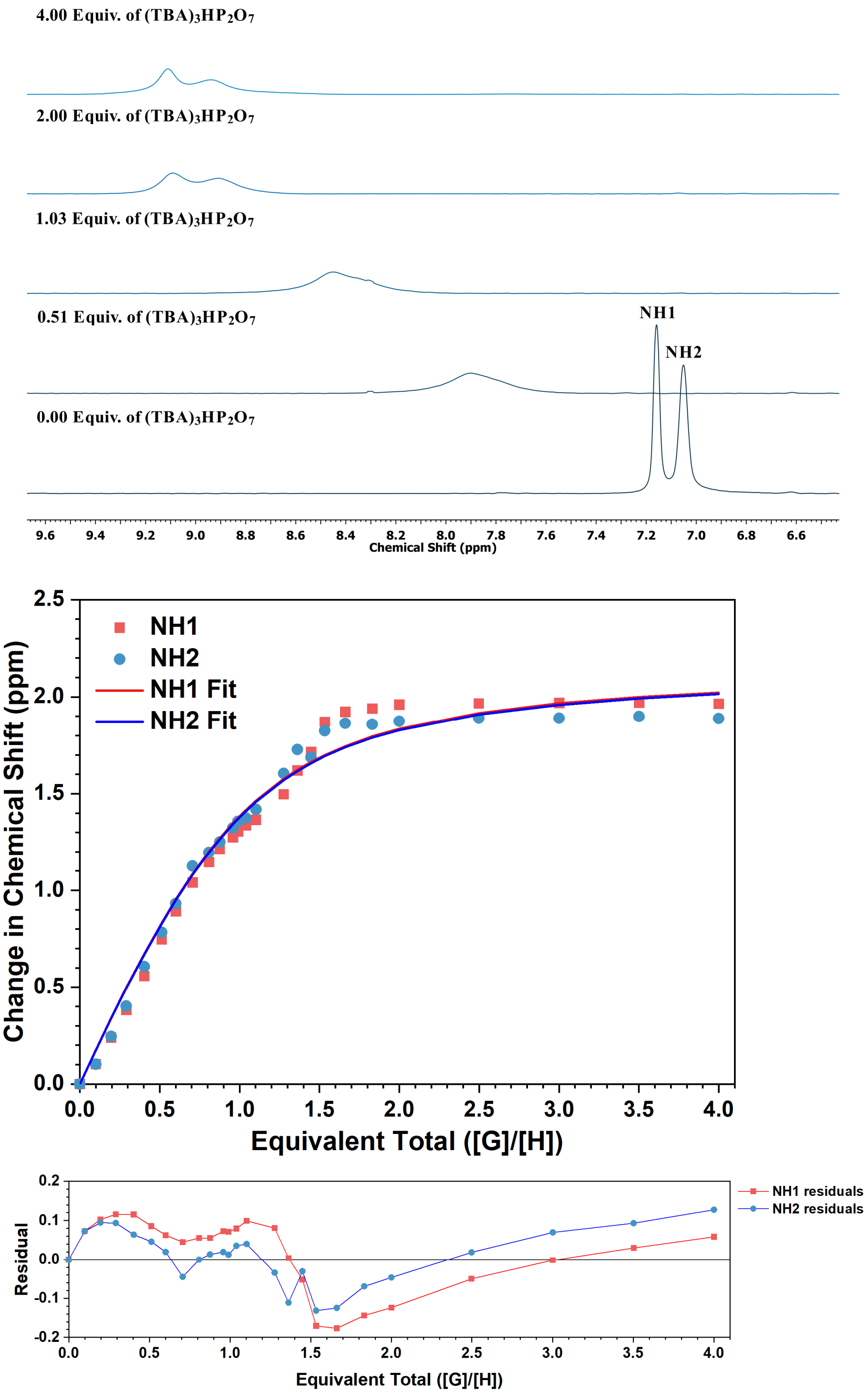

Figure S67: The ^{1}H-NMR stacked plot of tetra *t*-BuS (**3**) (5 mM) titrated with $(TBA)_3HP_2O_7$ in DMSO-d_6/0.5% H_2O at 298 K (above). The fit plot of 1:1 receptor:anion binding and the residual error obtained from fitting to the 1:1 binding isotherm (below). No binding constants were obtained due to poor fitting, seen in the sigmoidal curve at the beginning of the titration data, with K_a error = 8.91%.

Tetra t-BuO (4) Titration Spectra:

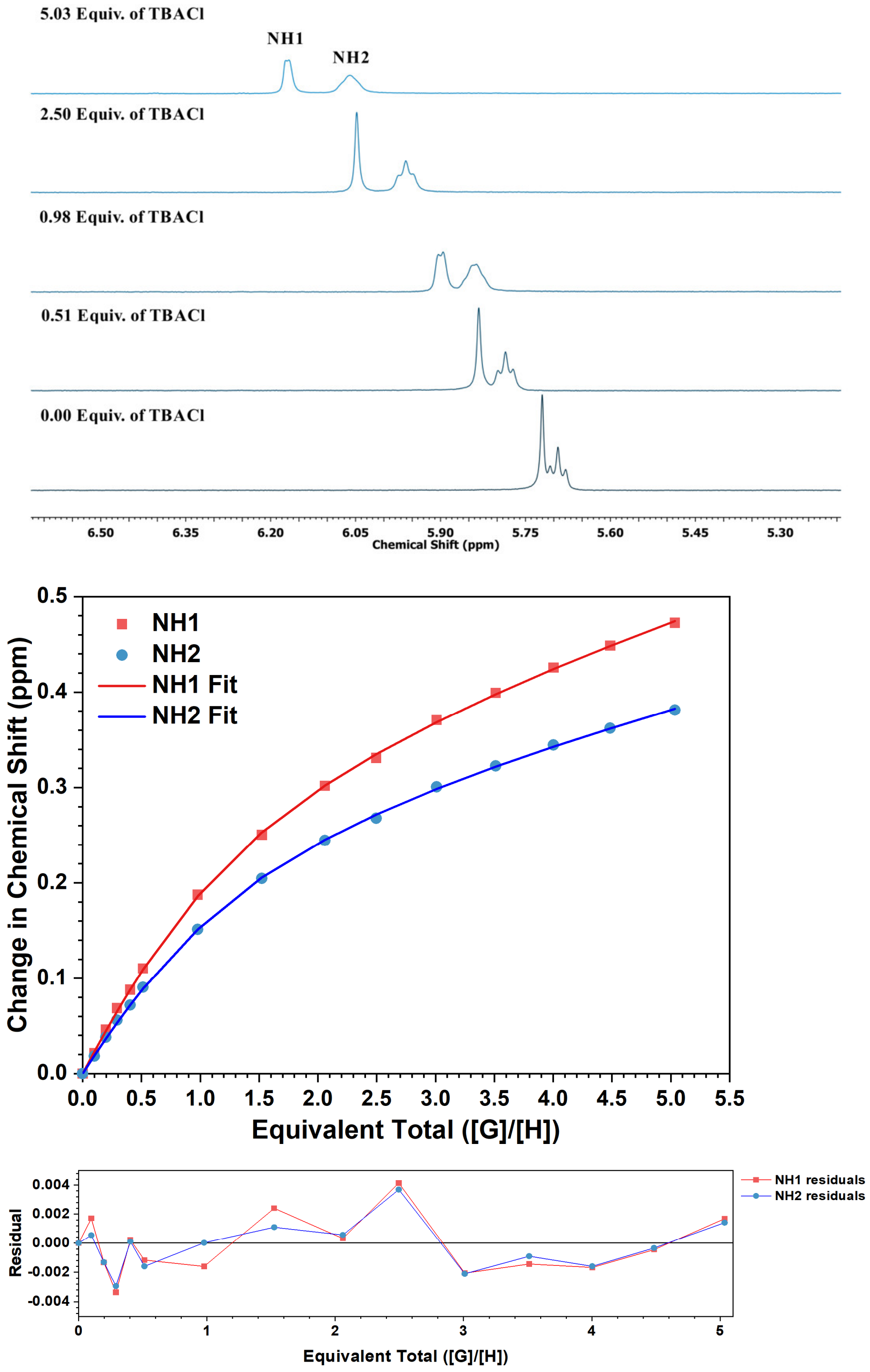

Figure S68: The ^{1}H-NMR stacked plot of tetra *t*-BuO (**4**) (5 mM) titrated with TBACl in DMSO-d_6/0.5% H_2O at 298 K (above). The fit plot of 1:2 receptor:anion binding and the residual error obtained from fitting to the 1:2 binding isotherm $K_{11} = 305$, $K_{12} = 7$ with K_{11} error = 3.34% and K_{12} error = 2.84% (below).

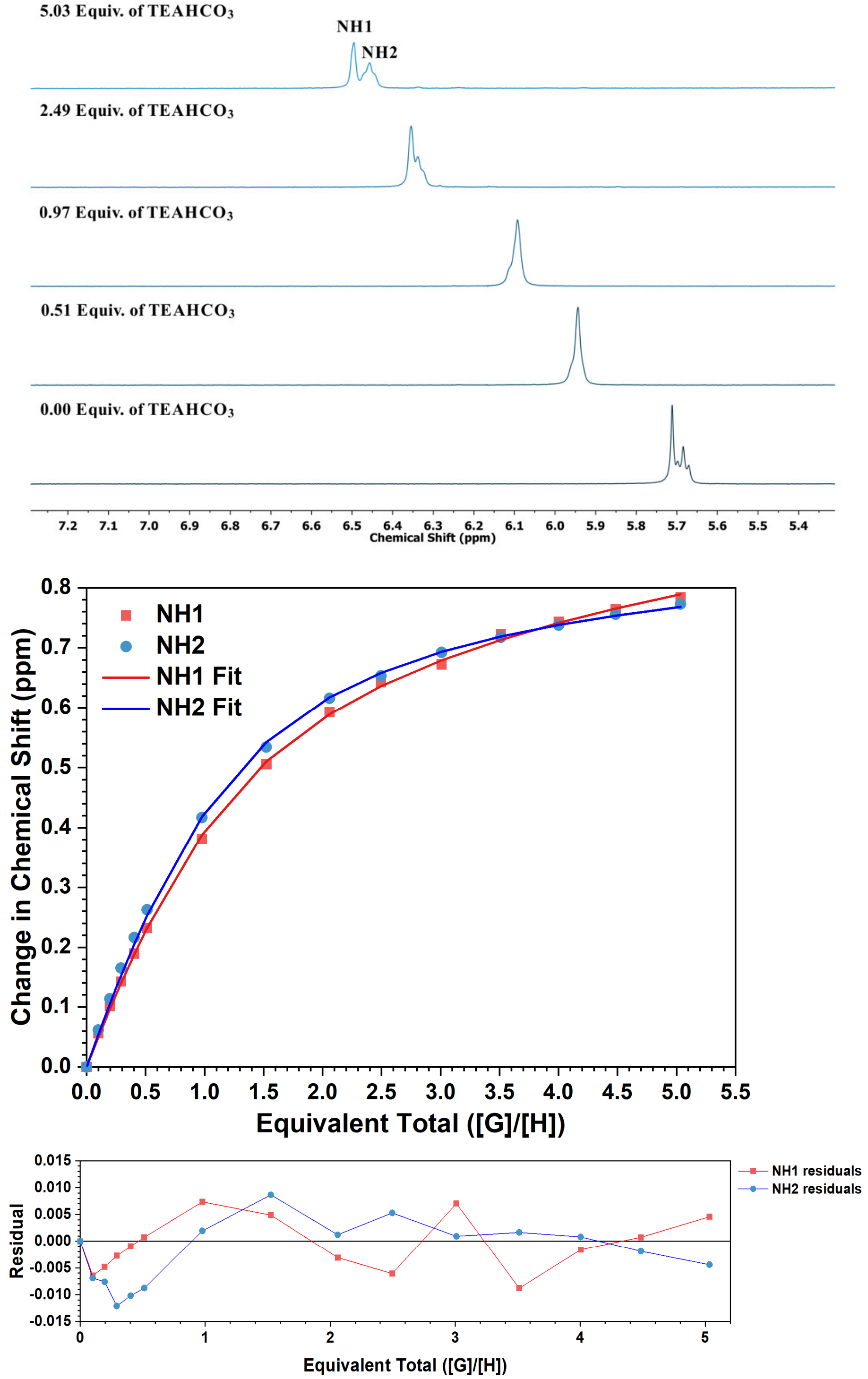

Figure S69: The ^{1}H-NMR stacked plot of tetra *t*-BuO (**4**) (5 mM) titrated with $TEAHCO_3$ in DMSO-d_6/0.5% H_2O at 298 K (above). The fit plot of 1:2 receptor:anion binding and the residual error obtained from fitting to the 1:2 binding isotherm K_{11} = 559, K_{12} = 21 with K_{11} error = 4.26% and K_{12} error = 7.67% (below).

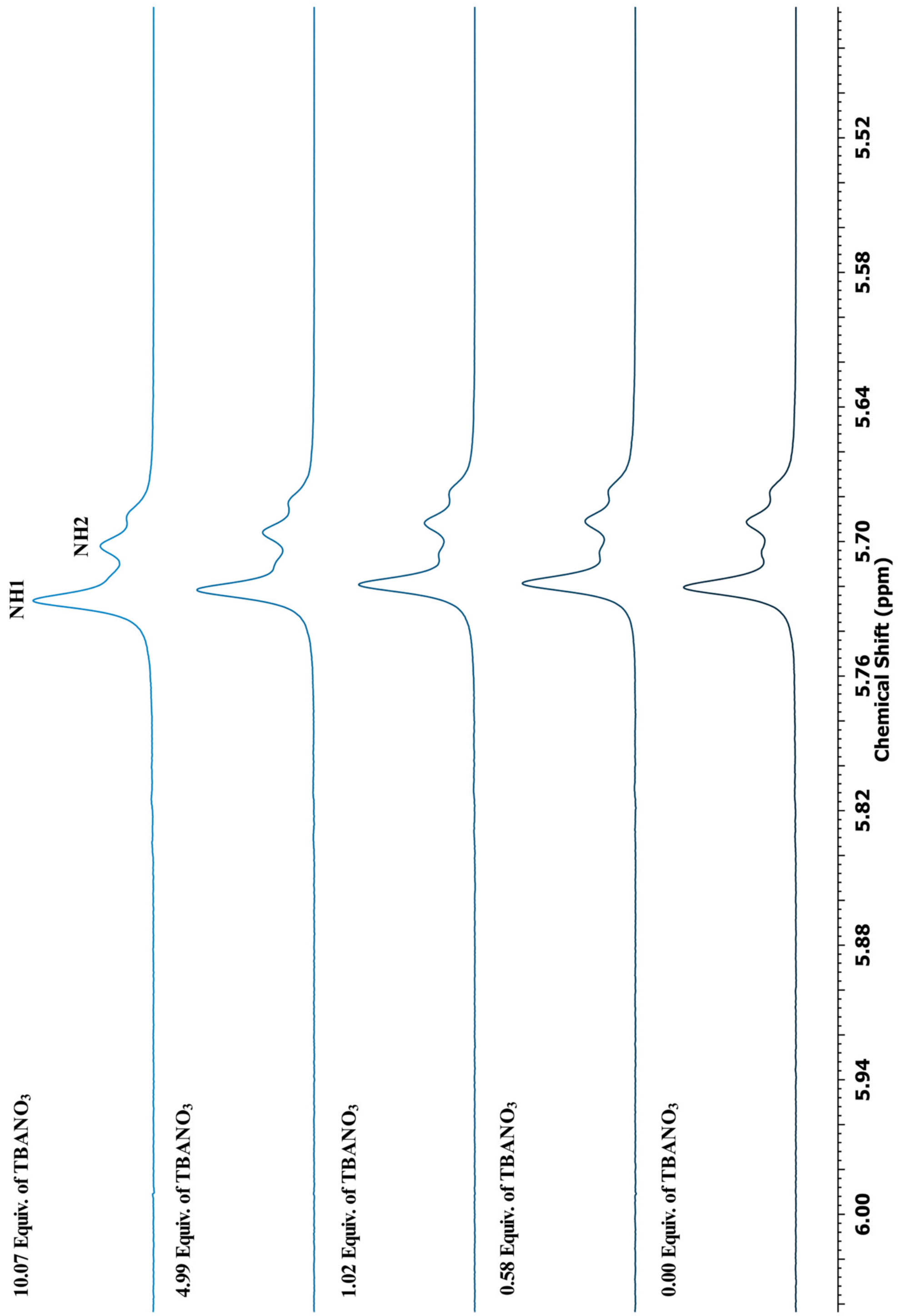

Figure S70: The ^{1}H-NMR stacked plot of tetra *t*-BuO (**4**) (5 mM) titrated with $TBANO_3$ in DMSO-d_6/0.5% H_2O at 298 K. There was no observed shifting from the protons so no binding isotherm graphs were made and no binding constants were found.

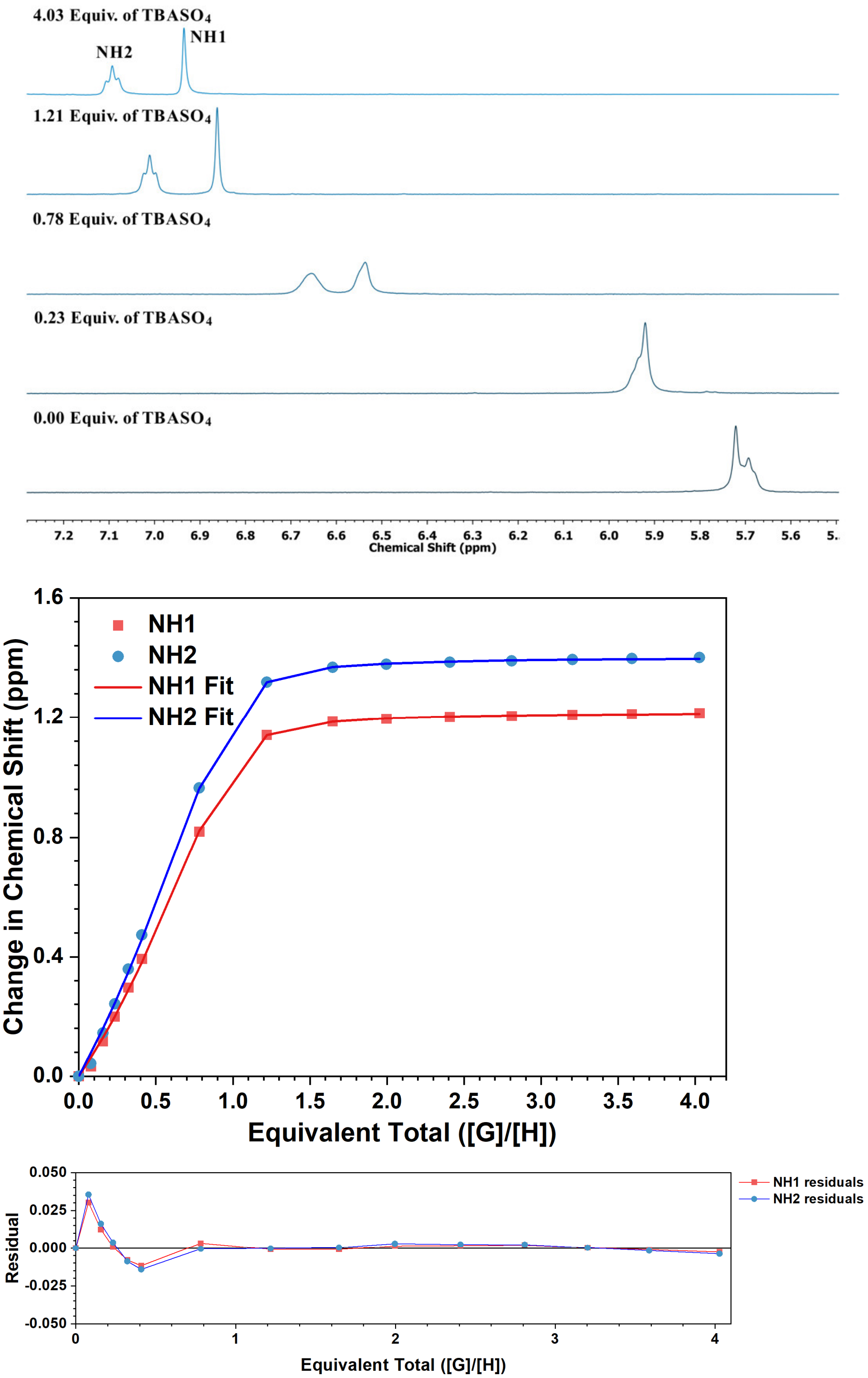

Figure S71: The ^{1}H-NMR stacked plot of tetra *t*-BuO (**4**) (5 mM) titrated with TBA_2SO_4 in DMSO-d_6/0.5% H_2O at 298 K (above). The fit plot of 2:1 receptor:anion binding and the residual error obtained from fitting to the 2:1 binding isotherm (below). No binding constants were obtained due to poor fitting, seen in the sigmoidal curve, with K_{11} error = 21.79% and K_{21} error = 11.85% (below).

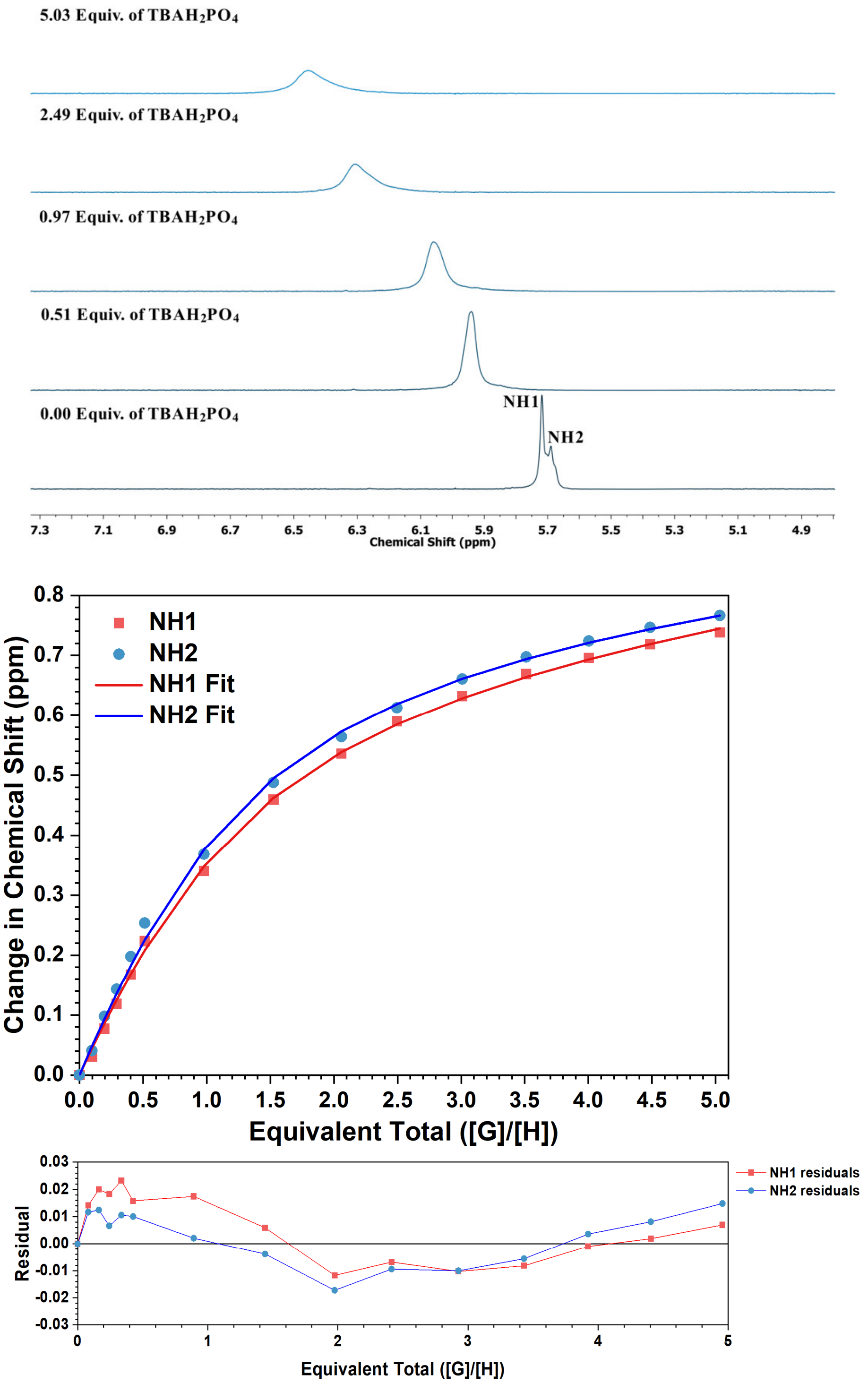

Figure S72: The ^{1}H-NMR stacked plot of tetra *t*-BuO (**4**) (5 mM) titrated with $TBAH_2PO_4$ in DMSO-d_6/0.5% H_2O at 298 K (above). The fit plot of 1:2 receptor:anion binding and the residual error obtained from fitting to the 1:2 binding isotherm (below). No binding constants were obtained due to poor fitting with K_{11} error = 6.28% and K_{12} error = 11.04% (below).

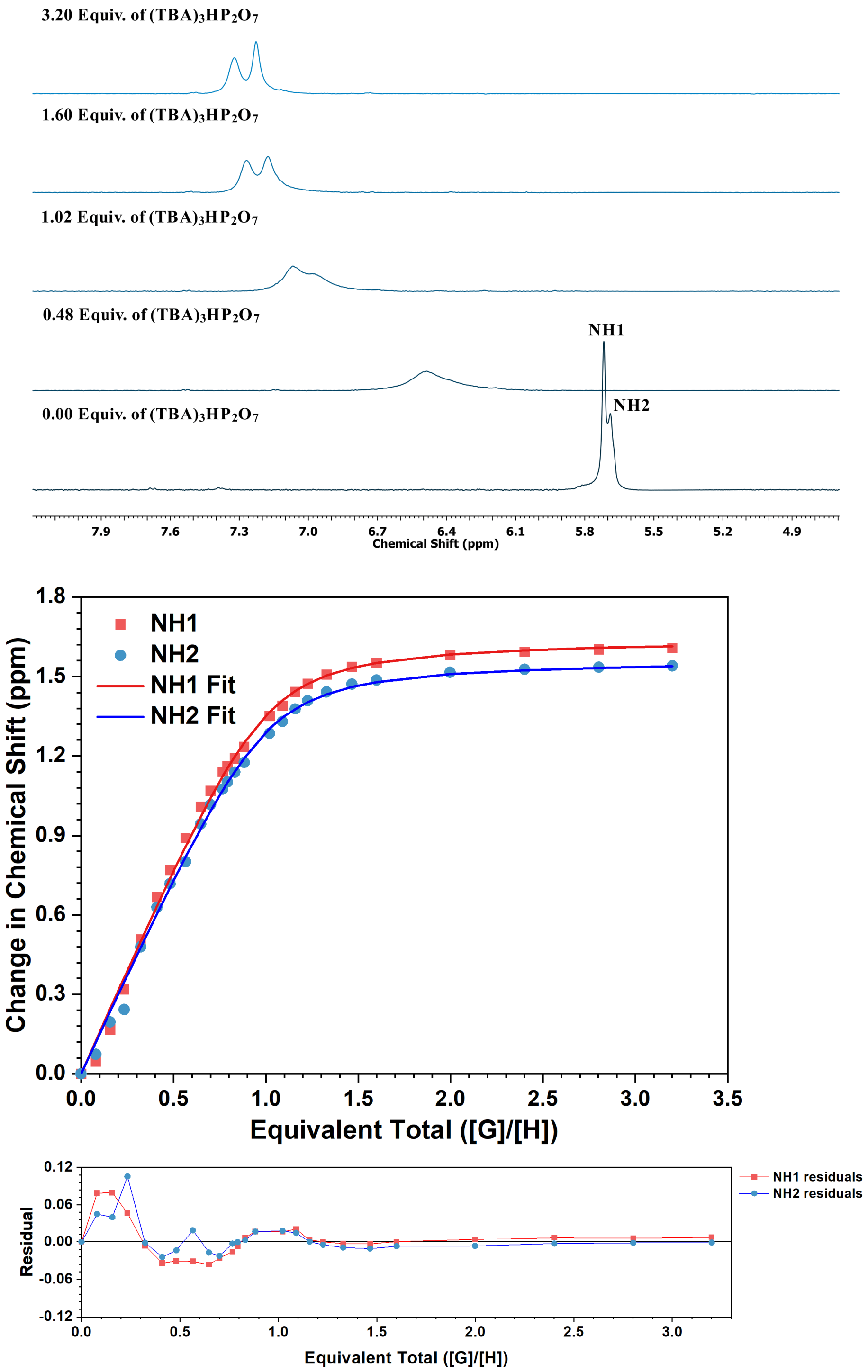

Figure S73: The ^{1}H-NMR stacked plot of tetra *t*-BuO (**4**) (5 mM) titrated with $(TBA)_3HP_2O_7$ in DMSO-d_6/0.5% H_2O at 298 K (above). The fit plot of 1:1 receptor:anion binding and the residual error obtained from fitting to the 1:1 binding isotherm $K_a = 4160$, with K_a error = 9.46% (below).

S5.2 ^{1}H-NMR Dilution Studies:

Calculation of the Dimerisation Constant:

After collecting and correcting the ^{1}H-NMR data to the DMSO-d_6 solvent peak (2.50 ppm) the NH1 and NH2 peak positions were recorded in an excel document alongside the respective concentration they were collected at. This document was processed through the online web-applet BindFit from http://supramolecular.org to produce the dimerisation constant (K_d) using the Nelder-Mead method of fitting [9].

This experiment resulted in an dimerisation constant of:

$$K_d = 10774.42\ M^{-1}\ (\pm\ 13.64\ \%).$$

Dilution Studies ^{1}H-NMR Stacked Plot, Fit Plot and Binding Isotherm:

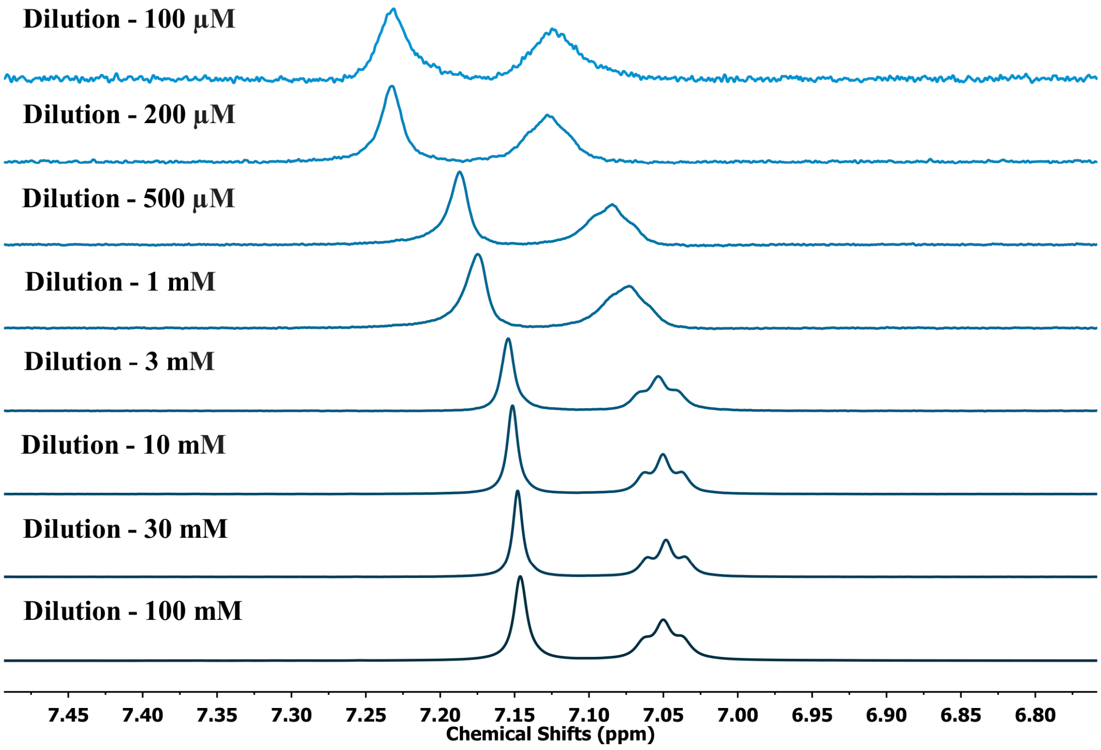

Figure S74: The ^{1}H-NMR stacked plot of tetra *t*-BuS (**3**) in DMSO-d_6 at the various concentrations (100mM, 30 mM, 10 mM, 3 mM, 1 mM, 500 µM, 200 µM and 100 µM).

Dilution Studies ^{1}H-NMR Stacked Plot, Fit Plot and Binding Isotherm (continued):

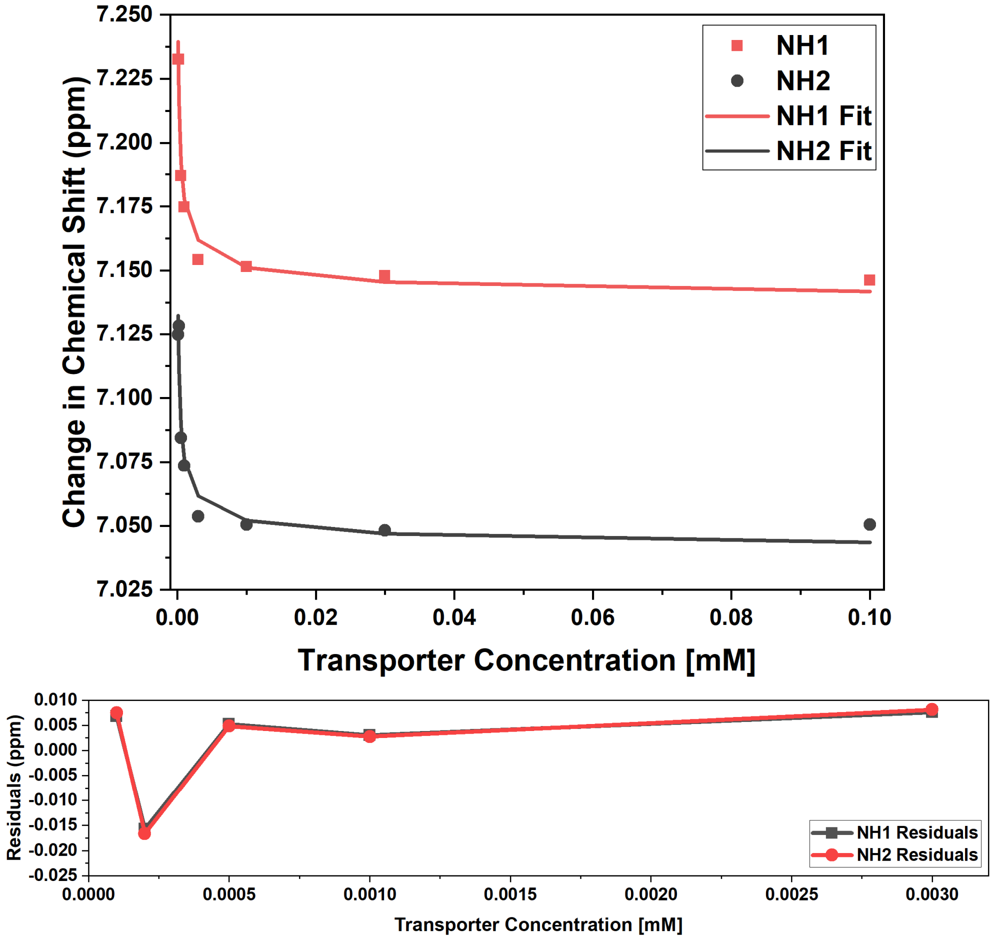

Figure S75: The Fit plot of tetra *t*-BuS (**3**) at the concentrations of 100 mM, 30 mM, 10 mM, 3 mM, 1mM, 500 µM, 200 µM and 100 µM in DMSO-d_6 at 298 K (above). The residual error obtained from fitting to the NMR Dimer Aggregation model and resulting binding isotherm K_d = 10774.42 M^{-1}, with K_d error = 13.64 % (below).

S6. Single crystal X-ray diffraction:

*Transporter **3**: Alert Level B: Low Bond Precision on C-C Bonds*

Due to small crystal sizes and needle morphology, pseudo-merohedral twinning was present after data collection. Here PLATON was used to generate an HKLF5 file for refinement. This reduces the size of the residuals, resulting in only a B-level alert for C-C bond uncertainties being too high, which is a consequence of data collection.

Table S10. Crystal and data refinement parameters for the X-ray studies of **3** and **4**.

Transporter	Tetra *t*-BuS (3)	Tetra *t*-BuO (4)
CCDC Deposition number	2033663	2033662
Formula	C30 H64 N10 S4	C30 H64 N10 O4
Formula weight	693.15	628.91
Temperature/K	100.15	100.15
Crystal system	Triclinic	Triclinic
Space group	*P*-1	*P*-1
a (Å)	6.4246(13)	9.3077(7)
b (Å)	10.1102(13)	14.3462(11)
c (Å)	15.777(2)	14.7537(13)
α(°)	73.562(12)	100.459(7)
β(°)	84.428(13)	90.934(7)
γ(°)	86.568(13)	91.316(6)
Volume (Å^3)	977.7(3)	1936.4(3)
Z	1	2
ρ_{calc} /g cm^{-3}	1.177	1.079
μ (mm^{-1})	2.490	0.587
F (000)	378	692
Reflections collected	3418	12919
Independent reflections	3418	7403
Data/restraints/parameters	3418/0/206	7403/0/409
GooF	1.113	1.028
Final R indexes [I>=2σ (I)]	R_1 = 0.1270, wR_2 = 0.3068	R_1 = 0.0846, wR_2 = 0.2123
Final R indexes [all data]	R_1 = 0.1854, wR_2 = 0.3480	R_1 = 0.1154, wR_2 = 0.2377
Largest diff. peak/hole / e Å^{-3}	1.098/−1.061	0.352/−0.323

Table S11. Hydrogen bonding parameters for transporter **3**.

Interaction	H···A (Å)	D···A (Å)	D–H···A (°)
**N(1)···S(2)#1**	2.53	3.370(11)	164.8
**N(2)···S(2)#1**	2.67	3.470(12)	154.6
**N(4)···S(1)#2**	2.76	3.552(12)	154.6
**N(5)···S(1)#2**	2.66	3.454(11)	153.4
**C(10)···S(1)#3**	3.13	4.091(16)	171.1

#1: 1-x, 1-y, 1-z; #2: 1-x, -y, 1-z; #3: 2-z, -y, 1-z.

Table S12. Hydrogen bonding parameters for transporter **4**.

Interaction	H···A (Å)	D···A (Å)	D–H···A (°)
**N(3)···O(2)#1**	2.02	2.827(4)	155.8
**N(4)···O(2)#1**	2.37	3.070(4)	138.7
**N(5)···O(1)#2**	2.28	3.006(3)	141.9
**N(6)···O(1)#2**	2.10	2.901(4)	155.5
**N(7)···O(4)#3**	2.15	2.928(3)	150.6
**N(8)···O(4)#3**	2.21	2.941(3)	143.3
**N(9)···O(3)#4**	2.36	3.075(3)	140.2
**N(10)···O(3)#4**	2.13	2.897(3)	147.7

#1: -x, 1-y, 1-z; #2: 1-x, 1-y, 1-z; #3: 2-x, 2-y, 1-z; #4: 1-x, 2-y, 1-z.

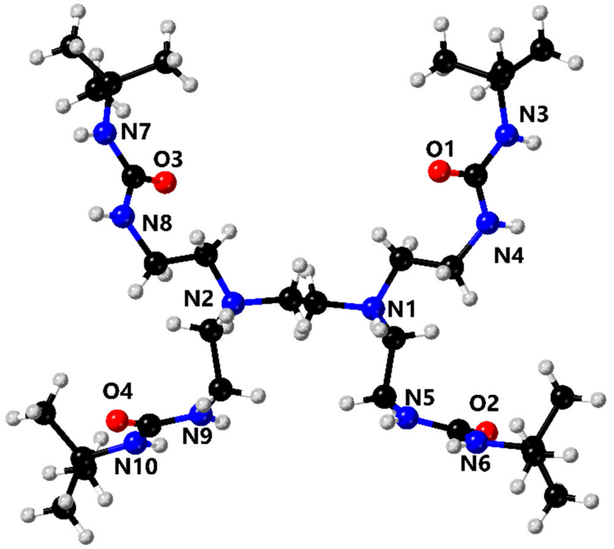

Figure S76: The X-ray crystal structure of tetra *t*-BuO (**4**).

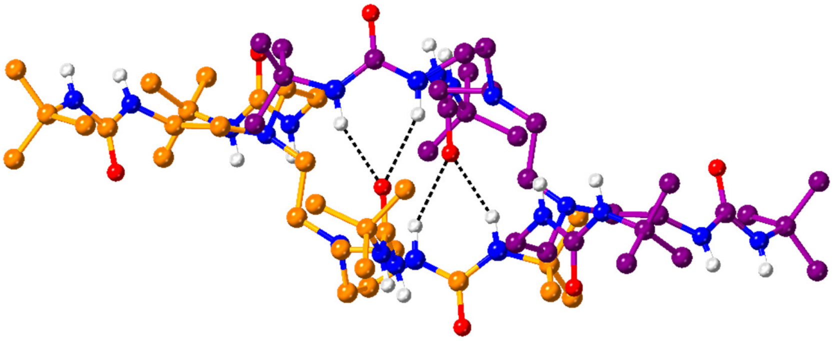

Figure S77: Intermolecular hydrogen bonding in the solid state in tetra *t*-BuO (**4**).

S7. References:

1. Avval, M.M.; Murthy, V.; Shashikanth, S. Synthesis and antimicrobial activity evaluation of poly ethylene imine (PEI) dendrimer modified with 1,3,4 oxadiazole derivatives. *Res. J. Pharm., Biol. Chem. Sci.* **2014**, *5*, 441-447.
2. Keypour, H.; Khanmohammadi, H.; Wainwright, K.P.; Taylor, M.R. Synthesis, crystal structures and ab initio studies of some heptaaza manganese (II) macrocyclic Schiff-base complexes with two 2-aminoethyl pendant arms. *Inorg. Chim. Acta* **2005**, *358*, 247-256.
3. Wu, X.; Gale, P.A. Small-molecule uncoupling protein mimics: synthetic anion receptors as fatty acid-activated proton transporters. *J. Am. Chem. Soc.* **2016**, *138*, 16508-16514.
4. Jowett, L.A.; Howe, E.N.; Wu, X.; Busschaert, N.; Gale, P.A. New Insights into the Anion Transport Selectivity and Mechanism of Tren‐based Tris‐(thio) ureas. *Chem. Eur. J.* **2018**, *24*, 10475-10487.
5. Jowett, L.A.; Ricci, A.; Wu, X.; Howe, E.N.; Gale, P.A. Investigating the influence of steric hindrance on selective anion transport. *Molecules* **2019**, *24*, 1278.
6. Tetko, I.V.; Gasteiger, J.; Todeschini, R.; Mauri, A.; Livingstone, D.; Ertl, P.; Palyulin, V.A.; Radchenko, E.V.; Zefirov, N.S.; Makarenko, A.S. Virtual computational chemistry laboratory–design and description. *J. Comput. Aid. Mol. Des.* **2005**, *19*, 453-463.
7. VCCLAB. Virtual Computational Chemistry Laboratory. Availabe online: https://www.vvclabs.org (accessed on 26/10/2020).
8. Thordarson, P. Determining association constants from titration experiments in supramolecular chemistry. *Chem. Soc. Rev.* **2011**, *40*, 1305-1323.
9. Bindfit - Fit data to 1:1, 1:2 and 2:1 Host-Guest equilibria. Availabe online: http://supramolecular.org (accessed on 28/10/20).
